# Small molecule purine and pseudopurine derivatives: synthesis, cytostatic evaluations and investigation of growth inhibitory effect in non-small cell lung cancer A549

**DOI:** 10.1080/14756366.2017.1414807

**Published:** 2017-12-22

**Authors:** Andrea Bistrović, Petra Grbčić, Anja Harej, Mirela Sedić, Sandra Kraljević-Pavelić, Sanja Koštrun, Janez Plavec, Damjan Makuc, Silvana Raić-Malić

**Affiliations:** a Department of Organic Chemistry, Faculty of Chemical Engineering and Technology, University of Zagreb, Zagreb, Croatia;; b Department of Biotechnology, Center for High-Throughput Technologies, University of Rijeka, Rijeka, Croatia;; c Chemistry Department, Fidelta Ltd., Zagreb, Croatia;; d Slovenian NMR Centre, National Institute of Chemistry, Ljubljana, Slovenia;; e En-FIST Centre of Excellence, Ljubljana, Slovenia;; f Faculty of Chemistry and Chemical Technology, University of Ljubljana, Ljubljana, Slovenia

**Keywords:** Purine, 1,2,3-triazole, purinomimetic, non-small cell lung cancer A549, p38 MAPK

## Abstract

Novel halogenated purines and pseudopurines with diverse aryl-substituted 1,2,3-triazoles were prepared. While *p*-(trifluoromethyl)-substituted 1,2,3-triazole in N-9 alkylated purine and 3-deazapurine was critical for strong albeit unselective activity on pancreatic adenocarcinoma cells CFPAC-1,1-(*p*-fluorophenyl)-1,2,3-triazole derivative of 7-deazapurine showed selective cytostatic effect on metastatic colon cancer cells SW620. Importantly, 1-(*p*-chlorophenyl)-1,2,3-triazole-tagged benzimidazole displayed the most pronounced and highly selective inhibitory effect in nM range on non-small cell lung cancer A549. This compound revealed to target molecular processes at the extracellular side and inside the plasma membrane regulated by GPLD1 and growth factor receptors PDGFR and IGF-1R leading to the inhibition of cell proliferation and induction of apoptosis mediated by p38 MAP kinase and NF-κB, respectively. Further optimisation of this compound as to reduce its toxicity in normal cells may lead to the development of novel agent effective against lung cancer.

## Graphical abstract

**Figure:**
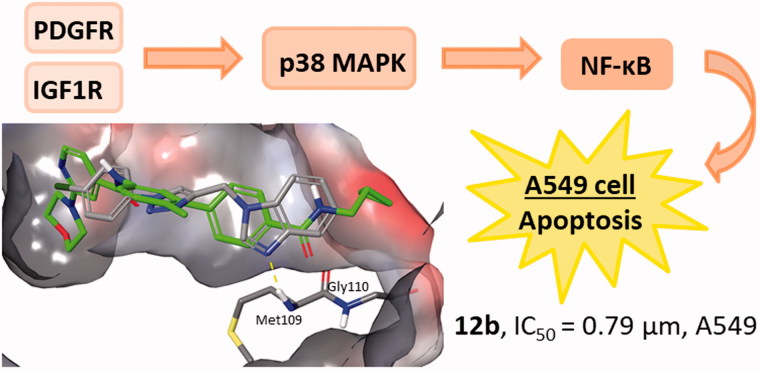

## Introduction

**Figure 1.**
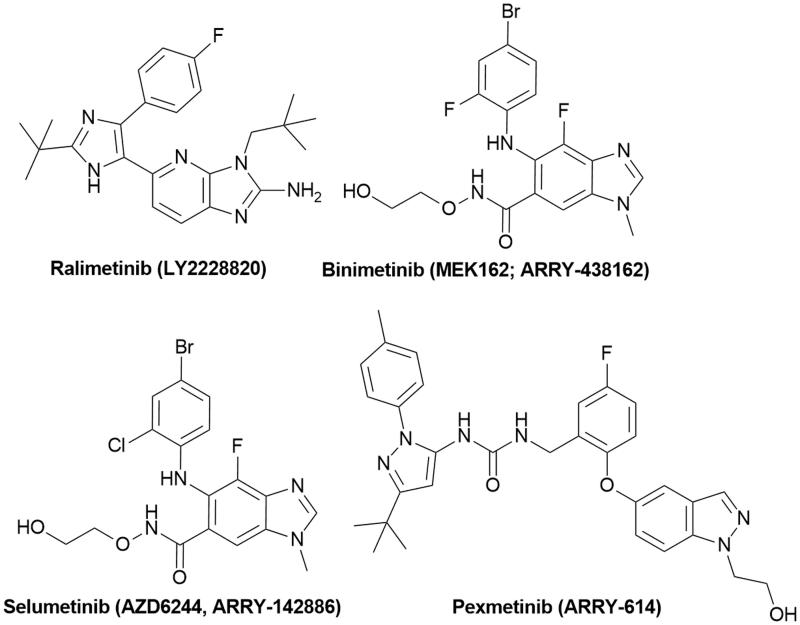
Roscivitine and purine isosteres as small-molecule inhibitors of CDK under clinical evaluations for the treatment of cancer.

Mitogen-activated protein kinases (MAPKs) are a family of kinases of different lineages that participate in intracellular signalling during proliferation, differentiation, cellular stress responses and apoptosis^1^ ^,^ ^2^. Over the recent years, a large body of evidence has revealed that overexpression and activation of MAPKs play a crucial role in the development and progression of cancer indicating the promising prospect of MAPK signalling as a valuable target in tumour therapy^3^. In search of MAPK pathway inhibitors, purine and purine-like scaffolds have been recognised as an important constituent of small molecules acting as highly potent MAPK inhibitors. Among them, ralimetinib (p38 MAPK inhibitor)^4^, binimetinib^5–7^, selumetinib (MEK1/2 inhibitors)^8^ ^,^ ^9^ and pexmetinib (Tie-2/p38 MAPK inhibitor)^10^ ^,^ ^11^ have reached the clinical trial stage for therapeutic application as anticancer drugs (Figure 1). Prevalence of halogenated drugs and the introduction of halogen atoms in pseudopurines presented in Figure 1 shows that halogenated compounds have been widely exploited in drug discovery indicating the importance of halogens in biological activity. In support of this finding, it was demonstrated that the formation of halogen bonds has been recognised to contribute to the stability of formed protein–ligand complexes^12–14^.

Over the recent years, p38 MAPK, as a member of the MAPK superfamily, has emerged as an important regulator of cancer progression^15^. Increased p38 MAPK phosphorylation has been reported in human lung tumours compared with normal tissue^16^, suggesting that this pathway contributes to lung tumour progression. In addition, p38 MAPK cascade has been demonstrated to be involved in the pathogenesis of other cancer types, such as hepatocellular carcinoma (HCC)^17^. More recently, antiproliferative effect of imidazoline and *N*-isopropylamidine benzimidazoles on non-small cell lung cancer cells A549 was ascribed to downregulation of p38 MAPK activity^18^. Considering the aforementioned and as a continuation of our research project on development of new cytostatic agents, herein we have reported the synthesis of *N*-alkylated halogenated purine and purine isosteres with 1,4-disubstituted 1,2,3-triazole unit. Different contribution of diverse purine and purinomimetics, such as 6-chloropurine, 6-chloro-3-deazapurine, 6-chloro-7-deazapurine, 6-chloro-7-bromo-7-deazapurine, 5-fluoroindole, indole and benzimidazole on cytostatic effect was anticipated. Furthermore, the influence of the type and position of halogen atoms at both heterocycle and phenyl moiety on their antiproliferative activities was investigated. Finally, apoptosis induction, *in silico* prediction and structural analysis of biological targets and the evaluation of p38 MAPK/NF-κB signalling pathways, as potential mediators of cytostatic effects in non-small cell lung cancer cells (A549) were carried out.

## Materials and methods

### General

All the solvents and chemicals including starting purinomimetics (6-chloropurine, **1a**, 4-chloro-1 H-imidazo[4,5-c]pyridine, **1b**, 6-chloro-7-deazapurine, 1c, 7-bromo-6-chloro-7-deazapurine, **1d**, 5-fluoroindole, 1e, indole, **1f** and benzimidazole, **1g)**, as well as halogen-substituted phenyl azides (1-azido-4-fluorobenzene, 1-azido-4-chlorobenzene, 1-azido-2-fluorobenzene and 1-azido-4-(trifluoromethyl)benzene) were purchased from Aldrich (St. Louis, MO), Acros (Geel, Belgium) and Ark Pharm Inc. (Arlington Heights, IL) thin layer chromatography was performed on pre-coated Merck silica gel 60F-254 plates, while glass column slurry-packed under gravity with silica gel (Fluka, 0.063–0.2 mm) was employed for column chromatography. Melting points of compounds were determined using Kofler micro hot-stage (Reichert, Wien). One- (1D) and two-dimensional (2D) homonuclear and heteronuclear NMR spectra were recorded on a Varian Gemini 300 (300 and 75 MHz) or Varian Gemini 600 (600 and 150 MHz) as well as Agilent Technologies DD2 NMR (300 and 600 MHz) spectrometers. All data were recorded in dimethyl sulfoxide (DMSO)-d_6_ at 298 K. NMR chemical shifts were referenced to the residual solvent signal of DMSO at б 2.50 ppm for ^1^H and б 39.50 ppm for ^13^C. Individual resonances were assigned on the basis of their chemical shifts, signal intensities, multiplicity of resonances, H–H coupling constants and with the use of a set of 2D experiments: correlation spectroscopy (^1^H–^1^H COSY), heteronuclear single-quantum coherence (^1^H–^13^C HSQC) and heteronuclear multiple-bond correlation (^1^H–^13^C HMBC). Microwave-assisted syntheses were performed in a Milestone start S microwave oven using quartz cuvettes.

### Experimental procedures for the synthesis of compounds

6-Chloro-9-(prop-2-yn-1-yl)-9*H*-purine (**2a**)^19^, 6-chloro-9-(prop-2-yn-1-yl)-9*H*-purine (**3a**)^19^, 4-chloro-7-(prop-2-yn-1-yl)-7*H*-pyrrolo[2,3-*d*]pyrimidine (**2c**)^20^, 5-fluoro-1-(prop-2-yn-1-yl)-1*H*-indole (**2e**)^21^, 1-(prop-2-yn-1-yl)-1*H*-indole (**2f**)^22^, 1-(prop-2-yn-1-yl)-1*H*-benzo[*d*]imidazole (**2g**)^21^, 1-{[1-(4-fluorophenyl)-1*H*-1,2,3-triazol-4-yl]met-hyl}-1*H*-benzo[*d*]imidazole (**12a**)^21^, 1-{[1-(4-chlorophenyl)-1*H*-1,2, 3-triazol-4-yl]methyl}-1*H*-benzo[*d*]imidazole (**12b**)^21^, 1-{[1–(4-(trifluoromethyl)phenyl)-1*H*-1,2,3-triazol-4-yl]methyl}-1*H*-benzo[*d*]imidazole (**12c)** ^21^, 1-{[1–(2-fluorophenyl)-1*H*-1,2,3-triazol-4-yl]methyl}-1*H*-benzo[*d*]imidazole (**12d**)^21^ and 4-{4-[((1*H*-benzo[*d*]imidazol-1-yl)methyl)-1*H*-1,2,3-triazol-1-yl]methyl}-7-hydroxy-4a,8a-dihydro-2*H*-chromen-2-on (**12e**)^21^ and 4-(azidomethyl)-7-hydroxy-2*H*-chromen-2-one^23^ were prepared according to known procedures.

### General procedure for N-alkylation of compounds 2b, 3b and 2d

To a solution of the corresponding heterocyclic base in dry dimethylformamide (DMF), NaH (1.2 eq) was added and stirred for 30 min under argon atmosphere. Propargyl bromide (1.2 eq) was added and the reaction mixture was heated at 60 °C and stirred overnight. Solvent was evaporated and the residue was purified by column chromatography (CH_2_Cl_2_:CH_3_OH =60:1).

### 4-Chloro-1-(prop-2-yn-1-yl)-1 H-imidazo[4,5-c]pyridine (2 b)

Compound **2b** was prepared using the above-mentioned procedure using 4-chloro-imidazo[4,5-*c*]pyridine (**1b**) (100 mg, 0.65 mmol) to obtain **2b** as white powder (44.3 mg, 67%, m.p. = 101–103 °C). ^1^H (300 MHz, DMSO-d_6_): *δ* 8.52 (1H, s, H8), 8.21 (1H, d, *J* = 5.6 Hz, H2), 7.76 (1H, d, *J =* 5.6 Hz, H3), 5.29 (2H, d, *J =* 2.5 Hz, CH_2_), 3.60 (1H, t, *J =* 2.5 Hz, CCH).^13 ^C NMR (75 MHz, DMSO-d_6_) δ 152.4 (C6), 145.9 (2), 141.3 (C8), 141.2 (C4), 139.8 (C5), 107.0 (C3), 77.4 (CCH), 77.1 (CCH), 34.7 (CH_2_).

### 5-Bromo-4-chloro-7-(prop-2-yn-1-yl)-7 H-pyrrolo[2,3-d]pyrimidine (2d)

Compound **2d** was prepared using the above-mentioned procedure using 5-bromo-4-chloro-7*H*-pyrrolo[2,3-*d*]pyrimidine (**1d**) (400 mg, 1.72 mmol) to obtain **2d** as white powder (348.9 mg, 70%, m.p. > 250 °C). ^1^H (300 MHz, DMSO-d_6_): *δ* 8.73 (1H, s, 1H, H2), 8.08 (1H, s, H8), 5.15 (2H, d, *J* = 2.5 Hz, CH_2_), 3.50 (1H, t, *J* = 2.5 Hz, CCH). ^13^C (151 MHz, DMSO-d_6_): *δ* 151.3 (C2), 150.7 (C6), 149.6 (C4), 130.6 (C8), 114.1 (C5), 86.3 (C7), 78.0 (CCH), 76.3 (CCH), 34.2 (CH_2_).

### 4-Chloro-3-(prop-2-yn-1-yl)-3 H-imidazo[4,5-c]pyridine (3b)

Compound was prepared using the above-mentioned procedure using 4-chloro-imidazo[4,5-*c*]pyridine (**1b**)(100 mg, 0.65 mmol) to obtain **3b** as crude oil (24.1 mg, 36%). ^1^H NMR (600 MHz, DMSO*-d_6_*) δ 8.62 (1H, s, H8), 8.18 (1H, d, *J* = 5.5 Hz, H2), 7.74 (1H, d, *J* = 5.5 Hz, H3), 5.40 (2H, d, *J* = 2.4 Hz, CH_2_), 3.58 (1H, t, *J* = 2.5 Hz, CCH). ^13^C (75 MHz, DMSO-d_6_): *δ* 151.1 (C6), 148.7 (C8), 141.3 (C2), 133.2 (C4), 127.5 (C5), 115.1 (C3), 78.8 (CCH), 77.4 (CH_2_), 36.2 (CH_2_).

### General procedure for the synthesis of N-1 substituted 1,2,3-triazolyl purinomimetics

The corresponding *N*-propargylated heterocyclic base (**2a–f**, **3a** or **3 b**) was dissolved in a mixture of *t*-BuOH: H_2_O = 1: 1 and DMF. 1 M CuSO_4_ (0.3 eq), Cu(0) (0.8 eq) and the corresponding terminal azide (1.2 eq) were added. The reaction mixture was stirred under microwave irradiation (300 W) at 80 °C for 45 min. The solvent was removed under reduced pressure and the residue was purified by column chromatography using CH_2_Cl_2_: CH_3_OH= 60: 1, as an eluent.

#### 6-Chloro-9-{[1–(4-fluorophenyl)-1 H-1,2,3-triazol-4-yl]methyl}-9 H-purine (4a)

Compound **4a** was prepared using the above-mentioned procedure using compound **2a** (50 mg, 0.28 mmol) and 1-azido-4-fluorobenzene to obtain **4a** as white powder (43.4 mg, 47%, m.p. = 170–171 C). ^1^H (600 MHz, DMSO-d_6_): *δ* 8.83 (1H, s, H5'), 8.80 (1H, s, H2), 8.79 (1H, s, H8), 7.91–7.88 (2H, m, Ph''), 7.43 (2H, t, *J* = 8.8 Hz, Ph''), 5.72 (2H, s, CH_2_). ^13^C (151 MHz, DMSO-d_6_): *δ* 162.6; 161.0 (d, *J_CF_* = 246.0 Hz, Ph-q''), 151.9 (C6), 151.8 (C2), 149.2 (C4), 143.1 (C4'), 133.05; 133.0 (d, *J*_CF_ = 2.8 Hz, Ph-q''), 130.9 (C5), 122.6; 122.6 (d, *J_CF_* = 8.8 Hz, Ph''), 122.3 (C5'), 116.9; 116.7 (d, *J_CF_* = 23.3 Hz, Ph''), 39.0 (CH_2_), 38.9 (CH_2_). Anal. calcd. for C_14_H_9_ClFN_7_: C, 51.00; H, 2.75; N, 29.74. Found: C, 51.23; H, 2.56; N, 29.68.

#### 6-Chloro-9-{[1-(4-(trifluoromethyl)phenyl)-1H-1,2,3-triazol-4-yl]methyl}-9H-purine (4c)

Compound **4c** was prepared using the above-mentioned procedure using compound **2a** (50 mg, 0.28 mmol) and 1-azido-4-(trifluoromethyl)benzene to obtain **4c** as colourless crystals (72.6 mg, 72%, m.p. = 174–177 °C). ^1^H (300 MHz, DMSO-d_6_): *δ* 8.95 (1H, s, H5'), 8.85 (1H, s, H2), 8.81 (1H, s, H8), 8.12 (2H, d, *J* = 8.5 Hz, Ph''), 7.97 (2H, d, *J* = 8.6 Hz, Ph''), 5.75 (2H, s, CH_2_). ^13^C (151 MHz, DMSO-d_6_): *δ* 151.9 (C6), 151.8 (C2), 149.2 (C4), 143.5 (C4'), 139.3 (Ph-q''), 130.9 (C5), 129.2; 129.0; 128.8; 128.6 (q, *J_CF_* = 32.4 Hz, CCF_3_), 127.3; 127.3; 127.3; 127.2 (q, *J_CF_* = 3.6 Hz, Ph''), 124.7; 122.4 (d, *J_CF_* = 272.4 Hz, CCF_3_), 122.4 (C5'), 120.7 (C8), 39.0 (CH_2_). Anal. calcd. for C_15_H_9_ClF_3_N_7_: C, 47.44; H, 2.39; N, 25.82. Found: C, 47.38; H, 2.14; N, 26.09.

#### 6-Chloro-9-{[1-(2-fluorophenyl)-1H-1,2,3-triazol-4-yl]methyl}-9H-purine (4d)

Compound **4d** was prepared using the above-mentioned procedure using compound **2a** (50 mg, 0.28 mmol) and 1-azido-2-fluorobenzene to obtain **4d** as white powder (36.1 mg, 39%, m.p. = 143–145 °C). ^1^H (600 MHz, DMSO-d_6_): *δ* 8.85 (1H, s, H5'), 8.81 (1H, s, H2), 8.67 (1H, d, *J* = 1.8 Hz, H8), 7.80 (1H, td*, J* = 7.8, 1.5 Hz, Ph''), 7.64–7.50 (2 H, m, Ph''), 7.42 (1H, td, *J* = 8.0, 0.9 Hz, Ph''), 5.75 (2H, s, CH_2_). ^13^C (151 MHz, DMSO-d_6_): *δ* 154.7; 153.1 (d, *J_CF_* = 250.6 Hz, Ph-q''), 151.9 (C6), 151.9 (C2), 149.3 (C4), 142.6 (C4'), 131.6; 130.9 (d, *J_CF_* = 7.9 Hz, Ph''), 130.9 (C5), 126.1 (C8), 125.7; 125,7 (d, *J_CF_* = 3.8 Hz, Ph''), 125.5; 125.4 (d, *J_CF_* = 4.3 Hz, C5'), 124.7; 124.4 (d, *J_CF_* = 10.7 Hz, Ph-q''), 117.3; 117.2 (d, *J_CF_* = 19.5 Hz, Ph''), 38.9 (CH_2_). Anal. calcd. for C_14_H_9_ClFN_7_: C, 51.00; H, 2.75; N, 29.74. Found: C, 51.17; H, 2.82; N, 29.71.

#### 4-{[4-((6-Chloro-9H-purin-9-yl)methyl)-1H-1,2,3-triazol-1-yl]methyl}-7-hydroxy-2H-chromen-2-one (4e)

Compound **4e** was prepared using the above-mentioned procedure using compound **2a** (50 mg, 0.28 mmol) and 4-(azidomethyl)-7-hydroxy-2*H*-chromen-2-one (73.8 mg, 0.34 mmol) to obtain **4e** as yellow powder (37.4 mg, 33%, m.p. = 178–77 °C). ^1^H (600 MHz, DMSO-d_6_): *δ* 10.69 (1H, bs, OH''), 8.81 (1H, s, H8), 8.77 (1H, s, H2), 8.31 (1H, s, H5'), 7.65 (1H, d, *J* = 8.8 Hz, H6''), 6.81 (1H, dd, *J* = 8.8, 2.2 Hz, C7''), 6.75 (1H, s, H-8''), 5.87 (2H, s, CH_2_), 5.66 (2H, s, CH_2_), 5.55 (1H, s, H3''). ^13^C (75 MHz, DMSO-d_6_): *δ* 161.8 (C7''), 160.1 (C2''), 155.2 (C8a''), 154.9 (C4), 151.9 (C5), 151.8 (C2), 150.5 (C6), 149.3 (C4''), 142.4 (C4'), 126.2 (C5''), 125.1 (C5'), 113.3 (C6''), 109.5 (C4a''), 109.4 (C3''), 102.6 (C8''), 49.4 (CH_2_), 38.9 (CH_2_). Anal. calcd. for C_18_H_12_ClN_7_O_3_: C, 52.76; H, 2.95; N, 23.93. Found: C, 52.99; H, 3.06; N, 24.25.

#### 4-Chloro-1-{[1-(4-(trifluoromethyl)phenyl)-1H-1,2,3-triazol-4-yl]methyl}-1H-imidazo[4,5-c]pyridine (5c)

Compound **5c** was prepared using the above-mentioned procedure using compound **2b** (25 mg, 0.13 mmol) and 1-azido-4-(trifluoromethyl)benzene (0.31 ml, 0.16 mmol) to obtain **5c** as white powder (39.5 mg, 80%, m.p. = 151–154 °C). ^1^H NMR (300 MHz, DMSO-d_6_) δ 8.99 (1H, s, H5'), 8.63 (1H, s, H8), 8.15 (3H, m, H2; Ph''), 7.97 (2H, d, *J* = 8.7 Hz, Ph''), 7.79 (1H, d, *J* = 5.6 Hz, H3), 5.78 (2H, s, CH_2_).^13^C (75 MHz, DMSO-d_6_): *δ* 146.3 (C8), 143.4 (C4'), 141.1 (C6), 140.9 (C2), 140.0 (C4), 139.2 (C5), 128.7 (m, Ph-q''), 127.2 (q, *J* = 3.5 Hz, Ph''), 122.4 (C5'), 122.1 (m, CF_3_), 120.6 (Ph''), 106.9 (CH-3), 40.0 (CH_2_). Anal. calcd. for C_16_H_10_ClF_3_N_6_: C, 50.74; H, 2.66; N, 22.19. Found: C, 50.58; H, 2.34; N, 22.37.

#### 4-Chloro-1-{[1–(2-fluorophenyl)-1H-1,2,3-triazol-4-yl]methyl}-1H-imidazo[4,5-c]pyridine (5d)

Compound **5d** was prepared using the above-mentioned procedure using compound **2b** (25 mg, 0.13 mmol) and 1-azido-2-fluorobenzene (0.31 ml, 0.16 mmol) to obtain **5d** as white crystals (30.9 mg, 72%, m.p. = 124–127 °C). ^1^H NMR (300 MHz, DMSO-d_6_) δ 8.72 (1H, d, *J* = 2.2 Hz, H5'), 8.63 (1H, s, H8), 8.17 (1H, d, *J* = 5.6 Hz, H2), 7.85–7.77 (2H, m, H3; Ph''), 7.65–7.50 (2H, m, Ph''), 7.46–7.38 (1H, m, Ph''), 5.77 (2H, s, CH_2_). ^13^C (75 MHz, DMSO-d_6_): *δ* 155.5; 152.1 (d, *J_CF_* = 250.4 Hz, Ph-q''), 146.4 (C8), 142.6 (C4'), 141.0 (C2), 140.1 (C6), 137.2 (C4), 136.3 (C5), 131.6; 131.5 (d, *J_CF_* = 8.0 Hz, Ph''), 126.0 (C5'), 125.7; 125.6 (d, *J_CF_* = 3.8 Hz, Ph''), 125.5; 125.5 (d, *J_CF_* = 4.7 Hz, Ph''), 117.6; 117.4 (d, *J_CF_* = 15.7 Hz, Ph-q''), 117.6 (d, *J* = 19.5 Hz, Ph''), 107.1 (C3), 38.9 (CH_2_). Anal. calcd. for C_15_H_10_ClFN_6_: C, 54.80; H, 3.07; N, 25.46. Found: C, 54.63; H, 3.28; N, 25.57.

#### 4-{[4-((4-Chloro-1H-imidazo[4,5-c]pyridin-1-yl)methyl-1H-1,2,3-triazol-1-yl]methyl}-7-hydroxy-2H-chromen-2-one (5e)

Compound **5e** was prepared using the above-mentioned procedure using compound **2b** (25 mg, 0.13 mmol) and 4-(azidomethyl)-7-hydroxy-2*H*-chromen-2-one (34.7 mg, 0.16 mmol) to obtain **5e** as yellow powder (53.1 mg, 60%, m.p. = 228–231 °C). ^1^H NMR (300 MHz, DMSO-*d_6_*) δ 8.58 (1H, s, H8), 8.31 (1H, s, H5'), 8.14 (1H, d, *J* = 5.6 Hz, H2), 7.70 (1H, d, *J* = 5.6 Hz, H3), 7.61 (1H, d, *J* = 8.7 Hz, H5''), 6.78 (1H, dd, *J* = 8.8, 1.8 Hz, H6''), 6.73 (1H, d, *J* = 1.6 Hz, H8''), 5.86 (2H, s, CH_2_), 5.69 (2H, s, CH_2_), 5.55 (1H, s, H3'')0.13 C NMR (151 MHz, DMSO-d_6_) δ 161.8 (C7''), 159.9 (C2''), 155.1 (C8a''), 150.2 (C4''), 146.3 (C8), 142.4 (C4'), 141.0 (C6), 140.8 (C2), 134.0 (C4), 137.1 (C5), 126.0 (C5''), 124.9 (5'), 113.2 (C6''), 109.4 (C3''), 109.2 (C4a''), 106.9 (C3), 102.5 (C8''), 49.3 (CH_2_), 40.1 (CH_2_). Anal. calcd. for C_15_H_10_ClFN_6_: C, 55.82; H, 3.20; N, 20.56. Found: C, 55.96; H, 3.22; N, 20.78.

#### 4-{[4-((6-Chloro-7H-purin-7-yl)methyl)-1H-1,2,3-triazol-1-yl]methyl}-7-hydroxy-2H-chromen-2-one (6e)

Compound **6e** was prepared using the above-mentioned procedure using compound **3a** (20 mg, 0.11 mmol) and 4-(azidomethyl)-7-hydroxy-2*H*-chromen-2-one (29.3 mg, 0.14 mmol) to obtain **6e** as white powder (14.9 mg, 33%, m.p. > 250 °C). ^1^H (600 MHz, DMSO-d_6_): 8.96 (1H, s, H8), 8.82 (1H, s, H2), 8.30 (1H, s, H5'), 7.63 (1H, d, *J* = 8.8 Hz, H5''), 6.80 (1H, dd, *J* = 8.8, 2.3 Hz, H6''), 6.76 (1H, d, *J* = 2.3 Hz, H8''), 5.87 (2H, s, CH_2_''), 5.85 (2H, s, CH_2_), 5.55 (1H, s, H3''). ^13^C (150 MHz, DMSO-d_6_): 161.8 (C7''), 161.6 (C4), 160.1 (C2''), 155.1 (C8a''), 151.8 (C2), 151.1 (C8), 150.3 (C4''), 143.3 (C4'), 126.2 (C5''), 124.4 (C5'), 122.1 (C5), 113.2 (C6''), 109.5 (C4a''), 109.2 (C3''), 102.6 (C8''), 49.3 (CH_2_''), 42.0 (CH_2_). Anal. calcd. for C_18_H_12_ClN_7_O_3_: C, 52.76; H, 2.95; N, 23.93. Found: C, 52.84; H, 3.75; N, 23.90.

#### 4-Chloro-3-{[1-(4-(trifluoromethyl)phenyl)-1H-1,2,3-triazol-4-yl]methyl}-3H-imidazo[4,5-c]pyridine (7c)

Compound **7c** was prepared using the above-mentioned procedure using compound **3b** (20 mg, 0.10 mmol) and 1-azido-4-(trifluoromethyl)benzene (0.24 ml, 0.12 mmol) to obtain **7c** as white powder (30.1 mg, 79%, m.p. = 183–185 °C). ^1^H (300 MHz, DMSO-d_6_): δ 8.98 (1H, s, H5'), 8.76 (s, 1H, H8), 8.16 (1H, d, 1H, *J* = 5.4 Hz, H2), 8.14 (2H, m, Ph''), 7.97 (2H, m, Ph), 7.76 (1H, d, *J* = 5.4 Hz, H3), 5.97 (2H, s, CH_2_). ^13^C (75 MHz, DMSO-d_6_): δ 151.0 (C4), 149.3 (C8), 144.8 (C4'), 140.7 (C2), 139.2 (Ph-q''), 132.9 (C6), 128.7 (q, *J_CF_* = 32.6 Hz, CCF_3'_'), 127.2 (C5), 127.1 (q, *J_CF_* = 3.8 Hz, Ph''), 122.0 (m, CCF_3_), 121.8 (C5'), 120.6 (Ph''), 114.9 (C3), 41.3 (CH_2_). Anal. calcd. for C_16_H_10_ClF_3_N_6_: C, 50.74; H, 2.66; N, 22.19. Found: C, 50.76; H, 2.58; N, 22.17.

#### 4-{[4-((4-Chloro-3H-imidazo[4,5-c]pyridin-3-yl)methyl)-1H-1,2,3-triazol-1-yl]methyl}-7-hydroxy-2H-chromen-2-one (7e)

Compound **7e** was prepared using the above-mentioned procedure using compound **3b** (15 mg, 0.08 mmol) and 4-(azidomethyl)-7-hydroxy-2 *H*-chromen-2-one (19.5 mg, 0.09 mmol) to obtain **7e** as white powder (23.8 mg, 74%, m.p. > 250 °C). ^1^H (300 MHz, DMSO-d_6_): 8.73 (1H, s, H8), 8.25 (1H, s, H5'), 8.15 (1H, d, *J* = 5.4 Hz, H2), 7.74 (1H, d, *J* = 5.4 Hz, H3), 7.63 (1H, d, *J* = 8.8 Hz, H5''), 6.79 (1H, dd, *J* = 8.8, 2.1 Hz, H6''), 6.74 (1H, d, *J* = 2.1 Hz, H8''), 5.88 (2H, s, CH_2_''), 5.86 (2H, s, CH_2_), 5.51 (1H, s, H3''). ^13^C (75 MHz, DMSO-d_6_): 161.7 (C7''), 159.9 (C2''), 155.1 (C8a''), 150.4 (C4), 150.2 (C4''), 143.8 (C4'), 140.7 (C2), 133.2 (C6), 127.5 (C5), 126.0 (C5''), 124.2 (C5'), 114.9 (C3), 113.2 (C6''), 109.1 (C4a''), 109.1 (C3''), 102.5 (C8''), 49.2 (CH_2_''), 41.5 (CH_2_). Anal. calcd. for C_15_H_10_ClFN_6_: C, 55.82; H, 3.20; N, 20.56. Found: C, 55.72; H, 3.16; N, 20.85.

#### 4-Chloro-7-{[1-(4-fluorophenyl)-1H-1,2,3-triazol-4-yl]methyl}-7H-pyrrolo[2,3-d]pyrimidine (8a)

Compound **8a** was prepared using the above-mentioned procedure using compound **2c** (100 mg, 0.56 mmol) and 1-azido-4-fluorobenzene (1.34 ml, 0.67 mmol) to obtain **8a** as white powder (131.6 mg, 74%, m.p. = 176–178 °C). ^1^H NMR (300 MHz, DMSO-d_6_) δ 8.74 (1H, s, H5'), 8.68 (1H, s, H2), 7.93–7.84 (3H, m, H6; Ph''), 7.43 (2H, t, *J* = 8.8 Hz, Ph''), 6.71 (1H, d, *J* = 3.6 Hz, H5), 5.68 (2H, s, CH_2_). ^13^C NMR (75 MHz, DMSO-*d_6_*) δ 163.4; 160.1 (d, *J_CF_* = 245.8 Hz, Ph-q'') 152.1 (C4), 150.8 (C7a), 150.6 (C2), 143.9 (C4'), 133.1; 133.1 (d, *J_CF_* = 2.8 Hz, Ph-q''), 131.4 (C6), 122.7; 122.6 (d, *J_CF_* = 8.9 Hz, Ph''), 122.2 (C5'), 117.0; 116.6 (d, *J_CF_* = 23.3 Hz, Ph''), 113.1 (C4a), 99.1 (C5), 39.6 (CH_2_). Anal. calcd. for C_15_H_10_ClFN_6_: C, 54.80; H, 3.07; N, 25.56. Found: C, 54.98; H, 2.94; N, 25.36.

#### 4-Chloro-7-{[1-(4-chlorophenyl)-1H-1,2,3-triazol-4-yl]methyl}-7H-pyrrolo[2,3-d]pyrimidine (8b)

Compound **8b** was prepared using the above-mentioned procedure using compound **2c** (100 mg, 0.56 mmol) and 1-azido-4-chlorobenzene (1.34 ml, 0.67 mmol) to obtain **8b** as white powder (107.8 mg, 56%, m.p. = 221–223 °C). ^1^H NMR (300 MHz, DMSO-d_6_) δ 8.79 (1H, s, H5'), 8.68 (1H, s, H2), 7.90 (2H, d, *J* = 8.9 Hz, Ph''), 7.86 (1H, d, *J* = 3.6 Hz, H6), 7.65 (2H, d, *J* = 8.9 Hz, Ph''), 6.71 (1H, d, *J* = 3.6 Hz, H5), 5.68 (2H, s, CH_2_). ^13^C (75 MHz, DMSO-d_6_): *δ* 150.7 (C4), 150.5 (C7a), 150.5 (C2), 144.0 (C4'), 135.3 (Ph-q''), 133.0 (Ph-q''), 131.3 (C6), 129.8 (Ph''), 121.9 (C5'), 121.8 (Ph''), 116.9 (C4a), 99.0 (C5), CH_2_ in DMSO. Anal. calcd. for C_15_H_10_Cl_2_N_6_: C, 52.19; H, 2.92; N, 24.35. Found: C, 52.12; H, 2.94; N, 24.29.

#### 4-Chloro-7-{[1-(4-(trifluoromethyl)phenyl)-1H-1,2,3-triazol-4-yl]methyl}-7H-pyrrolo[2,3-d]pyrimidine (8c)

Compound **8c** was prepared using the above-mentioned procedure using compound **2c** (50 mg, 0.28 mmol) and 1-azido-4-(trifluoromethyl)benzene (0.67 ml, 0.34 mmol) to obtain **8c** as white powder (84.2 mg, 80%, m.p. = 202–204 °C). ^1^H (300 MHz, DMSO-d_6_): *δ* 8.91 (1H, s, C5'), 8.68 (1H, s, H2), 8.13 (2H, d, *J* = 8.5 Hz, Ph''), 7.96 (2H, d, *J* = 8.6 Hz, Ph''), 7.87 (1H, d, *J* = 3.6 Hz, H6), 6.72 (1H, d, *J* = 3.6 Hz, H5), 5.71 (2H, s, CH_2_). ^13^C (75 MHz, DMSO-d_6_): *δ* 150.9 (C4), 150.7 (C7a), 150.7 (C2), 144.4 (C4'), 139.4 (Ph-q''), 131.5 (C6), 129.3; 128.8 (d, *J_CF_* = 32.3 Hz, CCF_3_), 127.4; 127.4; 127.3; 127.3 (q, *J_CF_* = 3.6 Hz, Ph''), 122.8; 122.1 (d, *J_CF_* = 272.3 Hz, CCF_3_), 122.3 (C5'), 120.9 (Ph''), 117.1 (C4a), 99.3 (C5), 39.6 (CH_2_). Anal. calcd. for C_16_H_10_ClF_3_N_6_: C, 54.74; H, 2.66; N, 22.19. Found: C, 54.54; H, 2.73; N, 22.00.

#### 4-Chloro-7-{[1-(2-fluorophenyl)-1H-1,2,3-triazol-4-yl]methyl}-7H-pyrrolo[2,3-d]pyrimidine (8d)

Compound **8d** was prepared using the above-mentioned procedure using compound **2c** (100 mg, 0.56 mmol) and 1-azido-2-fluorobenzene (1.34 ml, 0.67 mmol) to obtain **8d** as white crystals (53.3 mg, 29%, m.p. = 112–114 °C). ^1^H NMR (300 MHz, DMSO-d_6_) δ 8.68 (1H, s, H2), 8.61 (1H, d, *J* = 2.0 Hz, H5'), 7.88 (1H, d, *J* = 3.6 Hz, H6), 7.84–7.75 (1H, m, Ph''), 7.64–7.52 (2H, m, Ph''), 7.45–7.38 (1H, m, Ph''), 6.71 (1H, d, *J* = 3.6 Hz, H5), 5.70 (2H, s, CH_2_). ^13^C (75 MHz, DMSO-d_6_): *δ* 163.9; 160.3 (d, *J_CF_* = 246.0 Hz, Ph-q''), 152.1 (C4), 150.4 (C2), 150.1 (C7a), 143.2 (C4'), 132.1; 131.9 (d, *J_CF_* = 19.5 Hz, Ph-q''), 131.4 (Ph''), 131.3 (C6), 127.6 (C5'), 125.9 (Ph''), 125.5; 125.5 (d, *J_CF_* = 3.8 Hz, Ph''), 117.2; 116.9 (d, *J_CF_* = 19.6 Hz, Ph''), 117.0 (C4a), 99.0 (C5), 39.3 (CH_2_). Anal. calcd. for C_15_H_10_ClFN_6_: C, 54.80; H, 3.07; N, 25.56. Found: C, 54.81; H, 3.25; N, 25.69.

#### 4-{[4-((4-Chloro-7H-pyrrolo[2,3-d]pyrimidin-7-yl)methyl)-1H-1,2,3-triazol-1-yl]methyl}-7-hydroxy-2H-chromen-2-one (8e)

Compound **8e** was prepared using the above-mentioned procedure using compound **2c** (50 mg, 0.28 mmol) and 4-(azidomethyl)-7-hydroxy-2*H*-chromen-2-one (274.0 mg, 0.34 mmol) in DMF (0.5 ml) to obtain **8e** as white powder (15.1 mg, 40%, m.p. = 202–204 °C). ^1^H NMR (300 MHz, DMSO-d_6_) δ 8.65 (1H, s, H2), 8.22 (1H, s, H5'), 7.84 (1H, d, *J* = 3.6 Hz, H6), 7.63 (1H, d, *J* = 8.8 Hz, H5''), 6.78 (1H, dd, *J* = 9.0, 1.7 Hz, H6''), 6.71 (1H, d, *J* = 1.8 Hz, H8''), 6.69 (1H, d, *J* = 3.6 Hz, H5), 5.84 (2H, s, CH_2_), 5.62 (2H, s, CH_2_), 5.49 (1H, s, H3''). ^13^C NMR (75 MHz, DMSO-d_6_) δ 161.8 (C7''), 160.2 (C2''), 156.6 (C4), 155.2 (C8a''), 150.7 (C2), 150.6 (C4''), 145.0 (C7a), 143.4 (C4'), 131.6 (C6), 126.2 (C5''), 124.9 (C5'), 117.0 (C4a), 113.4 (C6''), 109.5 (C4a''), 109.4 (C3''), 102.7 (C8''), 99.1 (C5), 49.4 (CH_2_). Anal. calcd. for C_19_H_13_ClN_6_O_3_: C, 55.82; H, 3.20; N, 25.56. Found: C, 55.53; H, 3.16; N, 25.70.

#### 5-Bromo-4-chloro-7-{[1-(4-(trifluoromethyl)phenyl)-1H-1,2,3-triazol-4-yl]methyl}-7H-pyrrolo[2,3-d]pyrimidine (9c)

Compound **9c** was prepared using the above-mentioned procedure using compound **2d** (50 mg, 0.18 mmol) and 1-azido-4-(trifluoromethyl)benzene (0.43 ml, 0.22 mmol) to obtain **9c** as white powder (21.2 mg, 26%, m.p. = 220–221 °C). ^1^H NMR (300 MHz, DMSO-d_6_) δ 8.91 (1H, s, H5'), 8.72 (1H, s, H2), 8.16–8.10 (3H, m, H6; Ph''), 7.97 (2H, d, *J* = 8.7 Hz, Ph''), 5.69 (2H, s, CH_2_). ^13^C (75 MHz, DMSO-d_6_): *δ* 151.3 (C2), 150.1 (C7a), 147.6 (C4), 144.0 (C4'), 139.4 (Ph-q''), 131.3 (C6), 128.8 (Ph-q''), 127.4; 127.4; 127.3; 127.3 (q, *J_CF_* = 3.8 Hz, Ph''), 125.7; 122.2 (d, *J_CF_* = 281.9 Hz, CCF_3_), 122.4 (C5'), 120.8 (Ph''), 119.1 (C4a), 86.3 (C5), 38.9 (CH_2_). Anal. calcd. for C_16_H_9_BrClF_3_N_6_: C, 41.99; H, 1.98; N, 18.36. Found: C, 42.11; H, 1.92; N, 18.07.

##### 5-Bromo-4-chloro-7-{[1–(2-fluorophenyl)-1 H-1,2,3-triazol-4-yl]methyl}-7 H-pyrrolo[2,3-d]pyrimidine (9d)

Compound **9d** was prepared using the above-mentioned procedure using compound **2d** (50 mg, 0.18 mmol) and 1-azido-2-fluorobenzene (0.43 ml, 0.22 mmol) to obtain **9d** as white powder (18.4 mg, 25%, m.p. = 145–147 °C). ^1^H NMR (600 MHz, DMSO-*d_6_*) δ 8.72 (1 H, s, H2), 8.62 (1 H, d, *J* = 1.4 Hz, H5'), 8.12 (1 H, s, H6), 7.82–7.78 (1 H, m, Ph''), 7.63–7.53 (2 H, m, Ph''), 7.42 (1 H, t, *J* = 7.6 Hz, Ph''), 5.69 (2 H, s, CH_2_). ^13 ^C NMR (151 MHz, DMSO-*d_6_*) δ 162.2; 160.4 (d, *J_CF_* = 265.4 Hz, Ph-q''), 151.3 (C2), 150.7 (C4), 150.0 (C7a), 143.0 (C4'), 131.6; 131.6 (d, *J_CF_* = 8.0 Hz, Ph''), 131.3 (C6), 126.1 (C5'), 125.7; 125.7 (d, *J_CF_* = 3.6 Hz, Ph''), 125.5; 125.4 (d, *J_CF_* = 4.4 Hz, Ph''), 124.7; 124.6 (d, *J_CF_* = 10.9 Hz, Ph-q''), 117.3; 117.2 (d, *J_CF_* = 19.6 Hz, Ph''), 114.2 (C4a), 86.3 (C5), 40.0 (CH_2_). Anal. calcd. for C_15_H_9_BrClFN_6_: C, 44.20; H, 2.22; N, 20.62. Found: C, 43.99; H, 2.25; N, 20.48.

#### 4-{[4-((5-Bromo-4-chloro-7H-pyrrolo[2,3-d]pyrimidin-7-yl)methyl)-1H-1,2,3-triazol-1-yl]methyl}-7-hydroxy-2H-chromen-2-one (9e)

Compound **9e** was prepared using the above-mentioned procedure using compound **2d** (50 mg, 0.18 mmol) and 4-(azidomethyl)-7-hydroxy-2*H*-chromen-2-one (47.7 mg, 0.22 mmol) to obtain **9e** as yellow powder (30.7 mg, 35%, m.p. > 250 °C). ^1^H NMR (300 MHz, DMSO-d_6_) δ 8.69 (1H, s, H2), 8.25 (1H, s, H5'), 8.09 (1H, s, H6), 7.65 (1H, d, *J* = 8.7 Hz, H5''), 6.81 (1H, dd, *J* = 8.7, 2.4 Hz, H6''), 6.75 (1H, d, *J* = 2.3 Hz, H8''), 5.85 (2H, s, CH_2_), 5.60 (2H, s, CH_2_), 5.55 (1H, s, H3''). ^13^C NMR (75 MHz, DMSO-d_6_) δ 161.7 (C7''), 160.0 (C2''), 155.1 (C8a''), 150.4 (C4''), 149.2 (C2), 146.1 (C4), 144.1 (C7a), 142.7 (C4'), 131.3 (C6), 126.1 (C5''), 124.9 (C5'), 114.1 (C4a), 113.3 (C6''), 109.6 (C4a''), 109.4 (C3''), 102.6 (C8''), 93.7 (C5), 49.3 (CH_2_), 40.0 (CH_2_). Anal. calcd. for C_19_H_12_BrClN_6_O_3_: C, 46.79; H, 2.48; N, 17.23. Found: C, 46.79; H, 2.57; N, 17.05.

#### 1-{[1-(4-Fluorophenyl)-1H-1,2,3-triazol-4-yl]methyl}-5-fluoro-1H-indole (10a)

Compound **10a** was prepared using the above-mentioned procedure using **2e** (50 mg, 0.29 mmol) and 1-azido-4-fluorobenzene (0.70 ml, 0.35 mmol) to obtain **10a** as yellow oil (19.1 mg, 21%). ^1^H NMR (300 MHz, DMSO-*d_6_*) δ 8.77 (1H, s, H5'), 7.94–7.85 (2H, m, Ph), 7.65–7.60 (1H, m, H7), 7.56 (1H, d, *J* = 3.2 Hz, H2), 7.43 (2H, t, *J* = 8.8 Hz, Ph''), 7.31 (1H, dd, *J* = 9.9, 2.5 Hz, H4), 6.99 (1H, td, *J* = 9.3, 2.5 Hz, H6), 6.46 (1H, d, *J* = 3.1 Hz, H3), 5.55 (2H, s, CH_2_). ^13^C NMR (75 MHz, DMSO-d_6_) δ 163.4; 160.1 (d, *J_CF_* = 246.0 Hz, Ph-q''), 155.7 (C5), 144.6 (C4'), 135.5 (Ph-q''), 132.4 (C7a), 130.6 (C2), 124.3 (C3a), 122.7; 122.5 (d, *J_CF_* = 8.8 Hz, Ph''), 122.0 (C5'), 116.9, 116.6 (d, *J_CF_* = 23.3 Hz, Ph''), 111.3; 111.1 (d, *J_CF_* = 9.8 Hz, C7), 109.5; 109.2 (d, *J_CF_* = 26.2 Hz, C6), 105.2; 104.9 (d, *J_CF_* = 23.2 Hz, C4), 101.3; 101.3 (d, *J_CF_* = 4.5 Hz, C3), 41.0 (CH_2_). Anal. calcd. for C_17_H_12_F_2_N_4_: C, 65.80; H, 3.90; N, 18.05. Found: C, 65.78; H, 4.26; N, 17.86.

#### 1-{[(1–(4-Chlorophenyl)-1 H-1,2,3-triazol-4-yl]methyl}-5-fluoro-1 H-indole (10b)

Compound **10b** was prepared using the above-mentioned procedure using **2e** (50 mg, 0.29 mmol) to obtain **10b** as white powder (33.3 mg, 35%, m.p. = 117–120 °C). ^1^ H NMR (300 MHz, DMSO-d_6_) δ 8.82 (1H, s, H5'), 7.90 (2H, d, *J* = 9.0 Hz, Ph''), 7.65 (2H, d, *J* = 9.0 Hz, Ph''), 7.61 (1H, d, *J* = 4.6 Hz, H7), 7.56 (1H, d, *J* = 3.1 Hz, H2), 7.31 (1H, dd, *J* = 9.9, 2.5 Hz, H4), 6.99 (1H, td, *J* = 9.3, 2.5 Hz, H6), 6.46 (1H, dd, *J* = 3.1, 0.7 Hz, H3), 5.55 (2H, s, CH_2_). ^13^C NMR (75 MHz, DMSO-d_6_) δ 158.7 (C5), 144.6 (C4'), 135.3 (Ph-q''), 133.0 (C7a), 132.3 (Ph-q''), 130.6 (C2), 129.9 (C5'), 129.8 (Ph''), 126.2 (C3a), 121.8 (Ph''), 111.2; 111.1 (d, *J_CF_* = 9.9 Hz, C7), 109.5; 109.1 (d, *J_CF_* = 26.1 Hz, C6), 105.2; 104.9 (d, *J_CF_* = 23.3 Hz, C4), 101.2; 101.2 (d, *J_CF_* = 4.7 Hz, C3), 40.9 (CH_2_). Anal. calcd. for C_17_H_12_ClFN_4_: C, 62.48; H, 3.70; N, 17.15. Found: C, 62.33; H, 3.47; N, 17.01.

#### 5-Fluoro-1-{[1–(4-(trifluoromethyl)phenyl)-1 H-1,2,3-triazol-4-yl]methyl}-1 H-indole (10c)

Compound **10c** was prepared using the above-mentioned procedure using **2e** (50 mg, 0.29 mmol) and 1-azido-4-(trifluoromethyl)benzene (0.70 ml, 0.35 mmol) to obtain **10c** as white powder (73.2 mg, 70%, m.p. = 151–154 °C). ^1^H NMR (300 MHz, DMSO-d_6_) δ 8.95 (1H, s, H5'), 8.12 (2H, d, *J* = 8.4 Hz, Ph''), 7.96 (2H, d, *J* = 8.6 Hz, Ph''), 7.63 (1H, dd, *J* = 8.9, 4.6 Hz, H7), 7.57 (1H, d, *J* = 3.1 Hz, H2), 7.31 (1H, dd, *J* = 9.9, 2.5 Hz, H4), 6.99 (1H, td, *J* = 9.2, 2.5 Hz, H6), 6.47 (1H, d, *J* = 3.1 Hz, H3), 5.58 (2H, s, CH_2_). ^13^C NMR (75 MHz, DMSO-d_6_) δ 158.7 (C5), 144.9 (C4'), 139.3 (Ph-q''), 132.3 (C7a), 130.6 (C2), 128.9; 128.5 (d, *J_CF_* = 32.5 Hz, CCF_3_), 128.6 (C3a) 127.3; 127.2; 127.2; 127.1 (q, *J_CF_* = 3.7 Hz, Ph''), 125.3; 121.6 (d, *J_CF_* = 285.4 Hz, CCF_3_), 122.0 (C5'), 120.6 (Ph''), 111.2; 111.1 (d, *J_CF_* = 9.8 Hz, C7), 109.5; 109.1 (d, *J_CF_* = 26.1 Hz, C6), 105.2; 104.9 (d, *J_CF_* = 23.2 Hz, C4), 101.3; 101.2 (d, *J_CF_* = 4.7 Hz, C3), 40.9 (CH_2_). Anal. calcd. for C_18_H_12_F_4_N_4_: C, 60.00; H, 3.36; N, 15.55. Found: C, 59.83; H, 3.26; N, 15.78.

#### 5-Fluoro-1-{[1-(2-fluorophenyl)-1H-1,2,3-triazol-4-yl]methyl}-1H-indole (10d)

Compound **10d** was prepared using the above-mentioned procedure using **2e** (50 mg, 0.29 mmol) and 1-azido-2-fluorobenzene (0.70 ml, 0.35 mmol) in DMF (0.5 ml) and *t*-BuOH: H_2_O = 1:1 (4 ml) to obtain **10d** as yellow oil (19 mg, 21%). ^1^H NMR (300 MHz, DMSO-d_6_) δ 8.61 (1H, d, *J* = 2.2 Hz, H5'), 7.84–7.77 (1H, m, Ph''), 7.66 (1H, dd, *J* = 9.0, 4.4 Hz, H7), 7.58 (2H, d, *J* = 3.1 Hz, H2; Ph''), 7.40 (1H, dd, *J* = 6.9, 2.5 Hz, Ph''), 7.31 (1H, dd, *J* = 10.0, 2.5 Hz, H4), 7.03–6.94 (2H, m, Ph''; H6), 6.46 (1H, d, *J* = 3.1 Hz, H3), 5.57 (2H, s, CH_2_). ^13^C NMR (75 MHz, DMSO-d_6_) δ 158.3 (C5), 155.6; 152.0 (d, *J_CF_* = 271.7 Hz, Ph-q''), 146.7 (C4'), 132.3 (C7a), 131.3; 131.2 (d, *J_CF_* = 8.0 Hz, Ph''), 130.5 (C2), 125.8 (C5'), 125.5; 125.5 (d, *J_CF_* = 3.7 Hz, Ph''), 125.1 (Ph''), 124.5; 124.5 (d, *J_CF_* = 4.7 Hz, Ph-q''), 117.2; 116.9 (d, *J_CF_* = 19.4 Hz, Ph''), 111.2; 111.1 (d, *J_CF_* = 8.8 Hz, C7), 109.4; 109.1 (d, *J_CF_* = 26.4 Hz, C6), 105.1; 104.8 (d, *J_CF_* = 23.3 Hz, C4), 101.2; 101.1 (d, *J_CF_* = 4.5 Hz, C3), 40.7 (CH_2_). Anal. calcd. for C_17_H_12_F_2_N_4_: C, 65.80; H, 3.90; N, 18.05. Found: C, 65.75; H, 3.93; N, 18.16.

#### 4-{[4-((5-Fluoro-1H-indol-1-yl)methyl)-1H-1,2,3-triazol-1-yl]methyl}-7-hydroxy-2H-chromen-2-one (10e)

Compound **10e** was prepared using the above-mentioned procedure using compound **2e** (50 mg, 0.29 mmol) and 4-(azidomethyl)-7-hydroxy-2*H*-chromen-2-one (74.3 mg, 0.35 mmol) to obtain **10e** as white powder (67.3 mg, 59%, m.p. = 181–184 °C). ^1^H NMR (300 MHz, DMSO-d_6_) δ 10.69 (1H, s, OH''), 8.17 (1H, s, H5'), 7.64 (1H, d, *J* = 8.7 Hz, H5''), 7.60–7.54 (1H, m, H7), 7.53 (1H, d, *J* = 3.2 Hz, H2), 7.31 (1H, dd, *J* = 9.9, 2.5 Hz, H4), 6.97 (1H, td, *J* = 9.2, 2.5 Hz, H6), 6.81 (1H, dd, *J* = 8.7, 2.4 Hz, H6''), 6.76 (1H, d, *J* = 2.3 Hz, H8''), 6.45 (1H, d, *J* = 2.5 Hz, H3), 5.86 (2 H, s, CH_2_), 5.54 (2H, s, CH_2_), 5.51 (1H, s, H3''). ^13^C NMR (75 MHz, DMSO-d_6_) δ 161.6 (C7''), 159.9 (C2''), 158.7; 155.6 (d, *J_CF_* = 231.7 Hz, C5), 155.1 (C8a''), 150.5 (C4''), 144.1 (C4'), 132.3 (C7a), 130.6 (C2), 128.6; 128.4 (d, *J_CF_* = 10.2 Hz, C3a), 126.0 (C5''), 124.4 (C5'), 113.2 (C6''), 111.2; 111.0 (d, *J_CF_* = 10.0 Hz, C7), 109.4; 109.1 (d, *J_CF_* = 25.5 Hz, C6), 109.3 (C4a''), 109.3 (C3''), 105.2; 104.9 (d, *J_CF_* = 23.2 Hz, C4), 102.5 (C8''), 101.1; 101.1 (d, *J_CF_* = 4.5 Hz, C3), 49.1 (CH_2_), 41.0 (CH_2_). Anal. calcd. for C_21_H_15_FN_4_O_3_: C, 64.61; H, 3.87; N, 14.35. Found: C, 64.43; H, 3.83; N, 14.54.

#### 1-{[1-(4-Fluorophenyl)-1H-1,2,3-triazol-4-yl]methyl}-1H-indole (11a)

Compound **11a** was prepared using the above-mentioned procedure using **2f** (70 mg, 0.45 mmol) and 1-azido-4-fluorobenzene (0.81 ml, 0.66 mmol) to obtain **11a** as white powder (88.5 mg, 67%, m.p. = 141–144 °C). ^1^H NMR (600 MHz, DMSO-d_6_) δ 8.77 (1H, s, H5'), 7.90 (2H, dd, *J* = 9.1, 4.7 Hz, Ph''), 7.62 (1H, d, *J* = 8.0 Hz, H7), 7.54 (1H, d, *J* = 7.9 Hz, H4), 7.48 (1H, d, *J* = 3.1 Hz, H2), 7.42 (2H, t, *J* = 8.8 Hz, Ph''), 7.16–7.12 (1H, m, H5), 7.040–7.01 (1H, m, H6), 6.47 (1H, dd, *J* = 3.3, 0.5 Hz, H3), 5.54 (2H, s, CH_2_). ^13^C NMR (75 MHz, DMSO-d_6_) δ 163.2; 160.0 (d, *J*_CF_ = 245.7 Hz, Ph-q''), 144.6 (C4'), 135.5 (C7a), 133.1; 133.0 (d*, J*_CF_ = 2.9 Hz, Ph-q''), 128.6 (C2), 128.2 (C3a), 122.5; 122.4 (d, *J*_CF_ = 8.8 Hz, Ph''), 121.9 (C5'), 121.1 (C6), 120.4 (C4), 119.2 (C5), 116.8; 116.5 (d, *J_CF_* = 23.2 Hz. Ph''), 110.1 (C7), 101.1 (C3), 40.6 (CH_2_). Anal. calcd. for C_17_H_13_FN_4_: C, 69.85; H, 4.48; N, 19.17. Found: C, 70.13; H, 4.39; N, 19.11.

#### 1-{[1-(4-Chlorophenyl)-1H-1,2,3-triazol-4-yl]methyl}-1H-indole (11b)

Compound **11b** was prepared using the above-mentioned procedure using **2f** (70 mg, 0.45 mmol) and 1-azido-4-chlorobenzene to obtain **11b** as white powder (83.2 mg, 60%, m.p. = 168–171 °C). ^1^H NMR (300 MHz, DMSO-d_6_) δ 8.83 (1H, s, H5'), 7.90 (2H, d, *J* = 8.9 Hz, Ph''), 7.71–7.59 (3H, m, Ph''; H7), 7.54 (1H, d, *J* = 7.8 Hz, H4), 7.48 (1H, d, *J* = 3.1 Hz, H2), 7.14 (1H, t, *J* = 7.5 Hz, H5), 7.02 (1H, t, *J* = 7.4 Hz, H6), 6.47 (1H, d, *J* = 3.0 Hz, H3), 5.55 (2H, s, CH_2_). ^13^C NMR (151 MHz, DMSO-d_6_) δ 144.8 (C4'), 135.5 (C7a), 135.3 (Ph-q''), 132.9 (Ph-q''), 129.8 (Ph''), 128.6 (C2), 128.2 (C3a), 121.8 (Ph''), 121.7 (C5'), 121.1 (C6), 120.4 (C4), 119.1 (C5), 110.0 (C7), 101.1 (C3), 40.6 (CH_2_). Anal. calcd. for C_17_H_13_ClN_4_: C, 66.13; H, 4.24; N, 18.14. Found: C, 66.32; H, 4.32; N, 18.31.

#### 1-{[1-(4-(Trifluoromethyl)phenyl)-1H-1,2,3-triazol-4-yl]methyl}-1H-indole (11c)

Compound **11c** was prepared using the above-mentioned procedure using **2f** (70 mg, 0.45 mmol) and 1-azido-4-(trifluoromethyl)benzene (0.81 ml, 0.66 mmol) to obtain **11c** as white powder (110.9 mg, 72%, m.p. = 185–187 °C). ^1^H NMR (600 MHz, DMSO-d_6_) δ 8.95 (1H, s, H5'), 8.12 (2H, d, *J* = 8.5 Hz, Ph''), 7.96 (2H, d, *J* = 8.6 Hz, Ph''), 7.63 (1H, d, *J* = 8.4 Hz, H7), 7.55 (1H, d, *J* = 7.9 Hz, H4), 7.49 (1H, d, *J* = 3.2 Hz, H2), 7.17–7.12 (1H, m, H5), 7.04–7.01 (1H, m, H6), 6.47 (1H, dd, *J* = 3.1, 0.6 Hz, H3), 5.57 (2H, s, CH_2_). ^13^C NMR (75 MHz, DMSO-d_6_) δ 145.0 (C4'), 139.3 (Ph-q''), 135.5 (C7a), 129.2; 128.9; 128.4; 128.0 (q, *J_CF_* = 32.4 Hz, CCF_3_) 128.7 (C2), 128.3 (C3a), 127.2; 127.2; 127.1; 127.1 (q, *J_CF_* = 3.8 Hz, Ph''), 125.6; 122.0 (q, *J_CF_* = 272.0 Hz, CCF_3_), 121.9 (C5'), 121.2 (C6), 120.5 (Ph''), 120.4 (C4), 119.2 (C5), 110.0 (C7), 101.1 (C3), 40.6 (CH_2_). Anal. calcd. for C_18_H_13_F_3_N_4_: C, 63.16; H, 3.83; N, 16.37. Found: C, 62.96; H, 4.00; N, 16.30.

#### 1-{[1-(2-Fluorophenyl)-1H-1,2,3-triazol-4-yl]methyl}-1H-indole (11d)

Compound **11d** was prepared using the above-mentioned procedure using **2f** (70 mg, 0.45 mmol) and 1-azido-2-fluorobenzene (0.81 ml, 0.66 mmol) to obtain **11d** as yellow oil (37.4 mg, 28%). ^1^H NMR (600 MHz, DMSO-d_6_) δ 8.60 (1H, d, *J* = 1.8 Hz, H5'), 7.80 (1H, td, *J* = 7.8, 1.4 Hz, Ph''), 7.65 (1H, d, *J* = 8.3 Hz, H7), 7.61–7.57 (1 H, m, Ph''), 7.56–7.52 (2H, m, Ph''; H4), 7.50 (1H, d, *J* = 3.1 Hz, H2), 7.41 (1H, t, *J* = 7.7 Hz, Ph''), 7.14 (1H, t, *J* = 7.6 Hz, H5), 7.03 (1H, dt, *J* = 7.8, 3.9 Hz, H6), 6.46 (1H, d, *J* = 3.1 Hz, H3), 5.57 (2H, s, CH_2_). ^13^C NMR (151 MHz, DMSO-d_6_) δ 154.5; 152.8 (d, *J_CF_* = 250.2 Hz, Ph-q''), 144.1 (C4'), 135.5 (C7a), 131.3; 131.2 (d, *J_CF_* = 8.0 Hz, Ph-q''), 128.6 (C2), 128.2 (C3a), 125.8 (C5'), 125.5; 125.5 (d, *J_CF_* = 3.7 Hz, Ph''), 124.9; 124.8 (d, *J_CF_* = 4.6 Hz, Ph''), 124.7; 124.6 (d, *J_CF_* = 10.9 Hz, Ph''), 121.1 (C6), 120.4 (C4), 119.2 (C5), 117.1; 117.0 (d, *J_CF_* = 19.5 Hz, Ph''), 110.1 (C7), 101.1 (C3), 40.5 (CH_2_). Anal. calcd. for C_17_H_13_FN_4_: C, 69.85; H, 4.48; N, 19.17. Found: C, 69.92; H, 4.55; N, 19.17.

#### 4-{[4-((1H-Indol-1-yl)methyl)-1H-1,2,3-triazol-1-yl]methyl}-7-hydroxy-2 H-chromen-2-one (11e)

Compound **11e** was prepared using the above-mentioned procedure using compound **2e** (70 mg, 0.45 mmol) and 4-(azidomethyl)-7-hydroxy-2*H*-chromen-2-one (87.9 mg, 0.66 mmol) to obtain **11e** as yellow powder (78.8 mg, 47%, m.p. = 155–158 °C). ^1^H NMR (300 MHz, DMSO-d_6_) δ 10.69 (1H, s, OH''), 8.15 (1H, s, H7), 7.63 (1H, d, *J* = 8.7 Hz, H5''), 7.53 (2H, d, *J* = 8.9 Hz, H4; H5'), 7.44 (1H, d, *J* = 3.2 Hz, H2), 7.15–7.08 (1H, m, H5), 7.04–6.97 (1H, m, H6), 6.80 (1H, dd, *J* = 8.7, 2.3 Hz, H6''), 6.75 (1H, d, *J* = 2.3 Hz, H8''), 6.44 (1H, d, *J* = 3.1 Hz, H3), 5.84 (2H, s, CH_2_), 5.53 (1H, s, H3''), 5.50 (2H, s, CH_2_). ^13^C NMR (151 MHz, DMSO-d_6_) δ 161.6 (C7''), 159.8 (C2''), 155.1 (C8a''), 150.4 (C4''), 144.2 (C4'), 135.5 (C7a), 128.6 (C2), 128.2 (C3a), 126.0 (C5''), 124.3 (C5'), 121.1 (C6), 120.4 (C4), 119.1 (C5), 113.1 (C6''), 110.0 (C7), 109.3 (C4a''), 109.2 (C3''), 102.5 (C8''), 101.0 (C3), 49.1 (CH_2_), 40.8 (CH_2_). Anal. calcd. for C_21_H_16_N_4_O_3_: C, 67.73; H, 4.33; N, 15.04. Found: C, 67.62; H, 4.21; N, 14.92.

### Cell culturing

Human carcinoma cell lines A549 (lung carcinoma), HeLa (cervical carcinoma), SW620 (colorectal adenocarcinoma, metastatic), and CFPAC-1 (pancreatic cancer, derived from metastatic: liver), normal human lung (WI38) and foreskin (HFF-1) fibroblasts were obtained from the American Type Culture Collection (ATCC). Cells were cultured in humidified atmosphere at 37 °C with 5% CO_2_. As growth medium, Dulbecco’s modified Eagle medium (DMEM) was used with the addition of foetal bovine serum (10%), L-glutamine (2 mM) and antibiotics: streptomycin (100 µg/ml) and penicillin (100 U/ml).

### Proliferation assay

Cells were seeded onto 96-well microtiter plates at a seeding density of 3000 cells/well for carcinoma cell lines, and 5000 cells/well for normal human fibroblasts. The next day, cells were treated with test agents in five different concentrations (0.01–100 µM) and further incubated for 72 h. DMSO (solvent) was tested for potential cytotoxic effect but it did not exceed 0.1%. Following 72 h incubation, the MTT assay was performed and measured absorbances were transformed into percentage of cell growth as described previously^24^. Results were obtained from three independent experiments. IC_50_ values were calculated using linear regression analysis and results were statistically analysed by ANOVA, Tukey post-hoc test (*p* < .05).

### Apoptosis detection

Cells were seeded into 8-well chambers (Lab-tek II Chmaber Slides) in concentration of 2 × 10^4^ cells per well and treated with 2 × IC_50_ concentrations of selected compounds for 48 h. Staining of the cells was performed by Annexin-V-FITC Staining kit (Santa Cruz Biotechnology, Dallas, TX) according to the manufacturer’s instructions. Cells were visualised by fluorescent microscope (Olympus) at magnification of 40×.

### Western blot analysis

Cells were seeded in 6-well plates in the concentration depending on tested cell line varying from 1 × 10^5^ to 2 × 10^5^ cells/well. Cells were treated for 48 h with 2 × IC_50_ concentrations of selected compounds. Following treatment, cells were lysed with RIPA buffer supplemented with protease and phosphatase inhibitors (Roche). Total proteins (50 µg) were resolved on 10 or 12% polyacrilamide gels, depending on protein size, and transferred onto PVDF membranes that were blocked for 1 h with either 4% BSA or 5% non-fat milk prepared in tris-buffered saline (TBST). Membranes were probed with primary antibodies against GPLD1 (Abcam), PDGFRβ, p-IGF-1Rβ, p-p38 MAPK, and p-NF-κB-p65 from cell signalling technology at 4 °C overnight. The next day, membranes were washed in TBST and probed with horseradish peroxidase-conjugated secondary antibodies goat anti-mouse (Santa Cruz Biotechnology) or goat anti-rabbit (Santa Cruz Biotechnology). Protein bands were visualised using chemiluminescence substrate and ImageQuant LAS 500 (GE Healthcare, Chicago, IL). Following visualisation, protein band density was analysed by Quantity One 1-D Analysis Software (Bio-Rad, Hercules, CA).

### Computational methods

Calculated values of log*P*, *n*-octanol/water partition coefficients, for synthesised purine and purinomimetics were obtained by ChemAxon algorithm available within MarvinView Ver. 5.2.6. Predictions of biological targets of compound **12b** (Supplementary Table S1) were made by web-service PASS (http://www.pharmaexpert.ru/passonline/index.php), which is based on the identification of substructure features typical for active molecules^25^. Available crystal structures of apo- and co-crystallised p38-α kinase with various inhibitors were downloaded from protein data bank^26^ ^,^ ^27^. Representative ligands from the following DFG-in X-ray structure were used: 2GTN and 3GFE, as well as representative ligand from the following DFG-out X-ray structures: 3D83, whereby 2GTN is a Gly flip structure. IC_50_ inhibition values were collected from BindingDB^28^. The ligand docking studies were carried out using Glide docking protocol^29–32^ within Schrodinger suite of software^33^ with extra precision (XP). Docking was performed using unconstrained docking. Binding poses were refined and binding energy was estimated using MM-GBSA^34–36^ protocol and OPLS3 force-field with flexible residues distance being 5 Å.

## Results and discussion

### Chemistry

**Scheme 1.**
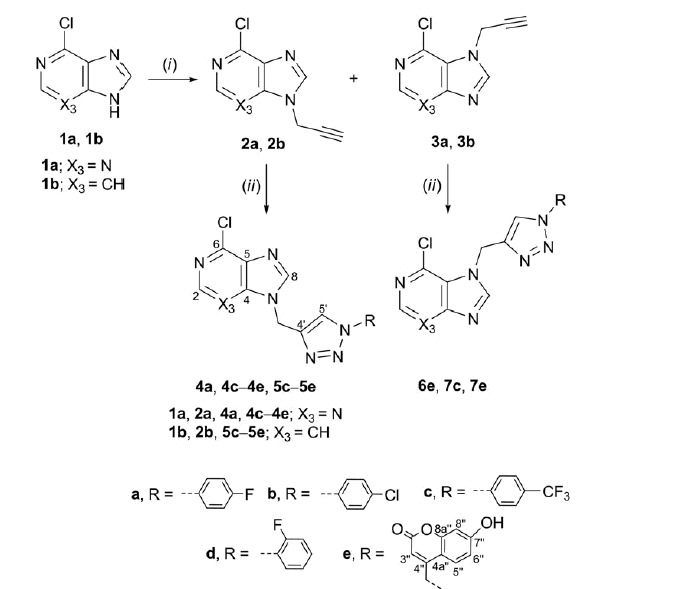
Synthesis of novel purine and 3-deazapurine derivatives with N-1 substituted 1,2,3-triazole. Reagents and conditions: (i) propargyl bromide, NaH, DMF, Ar atmosphere, 60 °C, 24 h; (ii) corresponding azide, Cu, 1 M CuSO_4_ solution, *tert*-butanol: H_2_O = 1: 1, MW 300 W, 80 °C, 45 min.

**Scheme 2.**
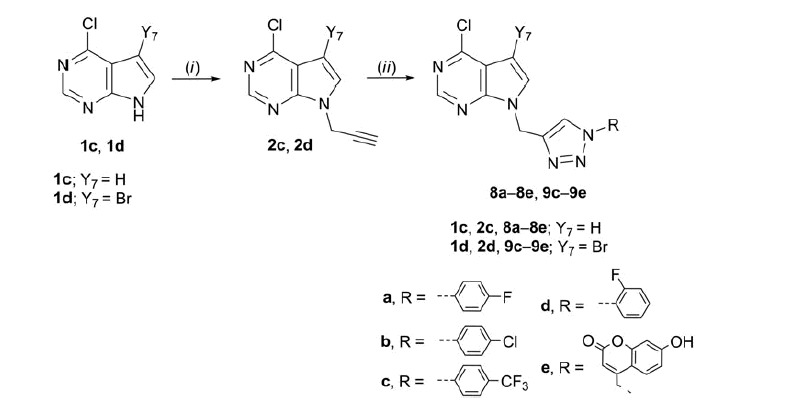
Synthesis of novel 7-deazapurine with *N*-1 substituted 1,2,3-triazole. Reagents and conditions: (i) propargyl bromide, K_2_CO_3_/NaH DMF, Ar atmosphere, 60 °C, 24 h; (ii) corresponding azide, Cu, 1 M CuSO_4_ solution, *tert*-butanol: H_2_O = 1: 1, MW 300 W, 80 °C, 45 min.

**Scheme 3.**
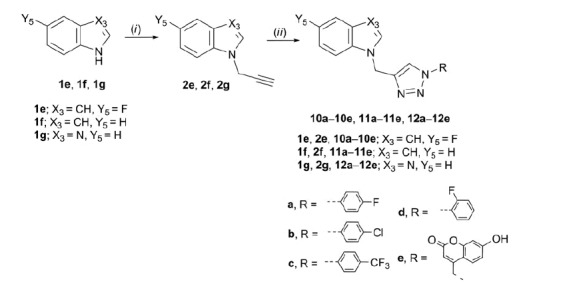
Synthesis of novel benzimidazole and indole derivatives with *N*-1 substituted 1,2,3-triazole. Reagents and conditions: (i) propargyl bromide, NaH, DMF, Ar atmosphere, 60 °C, 24 h; (ii) corresponding azide, Cu, 1 M CuSO_4_ solution, *tert*-butanol: H_2_O = 1: 1, MW 300 W, 80 °C, 45 min.

A focussed library of 33 purine and purine isosteres containing an aromatic 1-substituted 1,2,3-triazole moiety (**4a**, **4c**–**e**, **5c**–**e**, **6e**, **7c**, **7e, 8a**–**e**, **9c**–**e**, **10a**–**e**, **11a**–**e**, and **12a**–**e**) attached to varied heterocyclic bases: purine, imidazo[4,5-*c*]pyridine (3-deazapurine), pyrrolo[2,3-*d*]pyrimidine (7-deazapurine), benzimidazole, and indole were synthesised as shown in Schemes 1–3. *N*-Alkylation of the corresponding heterocyclic base with propargyl bromide in the presence of NaH, as a base, afforded the N-9- (**2a**–**d**) (Schemes 1 and 2) and N-7- (**3a** and **3b**) (Scheme 1) as well as N-1-propargylated (**2e**–**g**) (Scheme 3) heterocycles, as key intermediates.

Target regioselective 1,4-disubstituted 1,2,3-triazoles (**4a**, **4c**–**e**, **5c**–**e, 6e, 7c, 7e, 8a**–**e, 9c**–**e, 10a**–**e**, **11a**–**e**, and **12a**–**e**) were subsequently prepared by copper(I)-catalysed Huisgen 1,3-dipolar cyclo-addition of alkynyl derivatives of purine and purine isosteres with diverse halogen-substituted phenyl azides under microwave irradiation using copper(II) sulphate and metallic copper. Inspired by the recent discovery of coumarin–1,2,3-triazole–2-methylbenzimidazole hybrid that exerted strong cytotoxicity against HCC HepG2 cells^37^, the structural diversity of target compounds was further expanded to the synthesis of heterocyclic bases linked through 1,2,3-triazole to 7-hydroxycoumarin **4e**–**12e**. Therefore, click chemistry of 4-azidomethylcoumarin derivative and corresponding alkynes in the presence of Cu(I) afforded compounds **4e**–**12e**.

Generally, from the halogen-substituted phenyl azides, we found that strong electron-withdrawing trifluoromethyl substituent on the phenyl ring improved the yields in the click reaction of the target *p*-(trifluoromethyl)phenyl-1,2,3-triazole-tagged purine and purine isosteres **4c**–**12c**. Although reactions of N-7 regioisomers of alkynyl purine **3a** and 3-deazapurine **3 b** were performed with chosen phenyl azides only corresponding coumarin-1,2,3-triazolyl derivatives of N-7-(6-chloropurine) **6e** and N-7-(3-deazapurine) **7e**, and 4-(trifluoromethyl)phenyl-1,2,3-triazolyl derivative of N-7-(3-deazapurine) **7c** were successfully isolated indicating lower reactivity of N-7 regioisomers **3a** and **3 b** with respect to their N-9 counterparts. The synthesis of aryl-1,2,3-triazolyl N-1-benzimidazole **12a**–**e** was described in our previous reports^21^. In this study, we extended our investigations of these compounds towards evaluation of their cytostatic activities.

### NMR structure determination of regioisomeric purine and purine bioisosteres

The chemical structures of novel purine isosteres were confirmed by 1D and 2D NMR techniques. ^1^H and ^13^C NMR data are included in Supplementary Material. H2 and H3 protons of purine and 1*H*-imidazo[4,5-*c*]pyridine moiety in regioisomers **5c**, **5e**, **7c**, and **7e** showed characteristic chemical shifts between at *δ*_H_ 8.14–8.17 and 7.70–7.79 ppm, respectively. In addition, N7 regioisomers **7c** and **7e** exhibited more deshielded chemical shifts of H8 (*δ*_H_ 8.73–8.76 ppm) in comparison to N9 regioisomers **5c** and **5e** (*δ*_H_ 8.59–8.63 ppm). N7 and N9 regioisomers were further unambiguously distinguished by ^13^C NMR chemical shifts and their unique long-range correlation signals observed in ^1^H–^13^C HMBC spectra. N7 regioisomers showed ^13^C chemical shifts of C4 atoms at *δ*_C_ 150–151 ppm, whereas N9 regioisomers exhibited ^13^C chemical shifts at *δ*_C_ 140 ppm. In addition, C5 atoms were observed at *δ*_C_ 127–128 ppm and 137 ppm for N7 and N9 regioisomers, respectively. N7 regioisomers were confirmed by long-range correlation signals between methylene group (*δ*_H_ 5.7–6.0 ppm) and C5 atom, whereas methylene group of N9 regioisomers showed correlation signal with C4 atom.

### Biological evaluations

#### Antiproliferative evaluations

Results of antiproliferative evaluations of compounds **4a**, **4c**–**e**, **5c**–**e, 6e, 7c, 7e, 8a**–**e, 9c**–**e, 10a**–**e**, **11a**–**e**, and **12a**–**e** on human tumour cell lines including lung adenocarcinoma (A549), ductal pancreatic adenocarcinoma (CFPAC-1), cervical carcinoma (HeLa), and colorectal adenocarcinoma, metastatic (SW620), as well as on normal human lung (WI38) and foreskin (HFF-1) fibroblasts are presented in Table 1.

**Table 1.** The growth-inhibition effects *in vitro* of synthesised compounds on selected tumour and normal cell lines.


			IC_50_^a^ (µM)					
Compounds	Purine or pseudopurine	R	A549	CFPAC-1	HeLa	SW620	WI38/HFF-1^b^	ClogP^c^
**4a**			53.22	33.39	34.21	38.94	5.21	2.68
**4c**			15.91	7.90	12.30	14.69	0.75	3.44
**4d**			54.65	31.10	33.21	41.75	23.88	2.68
**4e**			88.95	>100	57.96	92.50	6.58	1.68
**5c**			9.43	7.39	22.52	11.77	5.21	3.29
**5d**			73.86	>100	>100	90.25	23.89	2.53
**5e**			>100	>100	>100	>100	60.49	1.53
**6e**			>100	>100	>100	>100	73.63	2.14
**7c**			>100	89.24	36.80	68.95	19.43	3.29
**7e**			>100	>100	8.77	>100	7.62	1.53
**8a**			85.36	48.37	39.41	8.50	32.18	3.44
**8b**			64.52	47.69	65.46	44.64	21.02	3.84
**8c**			46.91	46.55	38.83	37.05	4.62	4.20
**8d**			69.02	35.19	83.32	65.45	22.53	3.44
**8e**			69.19	36.27	33.59	73.48	39.93	2.44
**9c**			66.36	62.77	32.89	55.74	57.54	5.03
**9d**			86.36	65.10	20.03	65.90	48.47	4.27
**9e**			45.45	47.30	24.50	45.09	10.78	3.27
**10a**			>100	>100	>100	>100	>100	3.89
**10b**			40.16	>100	31.01	45.84	58.07	4.29
**10c**			71.57	64.71	53.01	54.25	24.03	4.65
**10d**			>100	>100	82.92	>100	>100	3.89
**10e**			51.93	77.08	47.63	57.55	45.70	2.89
**11a**			44.01	>100	>100	43.29	>100^b^	3.95
**11b**			>100	>100	>100	>100	>100^b^	4.41
**11c**			>100	>100	>100	>100	>100^b^	4.68
**11d**			51.27	85.56	71.15	44.83	40.42^b^	3.95
**11e**			75.38	>100	83.01	68.49	40.00^b^	3.17
**12a**			76.26	86.52	71.70	99.92	91.91	3.14
**12b**			**0.79**	52.55	42.17	91.23	38.10	3.60
**12c**			14.18	56.43	30.61	>100	>100	3.87
**12d**			36.25	34.68	21.94	>100	30.63	3.14
**12e**			>100	>100	>100	>100	>100	2.35

a 50% inhibitory concentration or compound concentration required inhibiting tumour cell proliferation by 50%.

b Compounds **11a–e** were tested in HFF-1 cell line.

c Values of *n*-octanol/water partition coefficients log*P* were calculated using ChemAxon algorithm (MarvinView Ver. 6.2.2.).

It can be noted that among purine-1,2,3-triazole hybrids (**4a**–**e**, **6e**), purine analogue **4c** with *p*-(trifluoromethyl)phenyl-substituted 1,2,3-triazole exhibited the highest antitumor activity, particularly in CFPAC-1 cells (IC_50_ = 7.90 µM). Besides rather non-selective inhibitory effects against all tested tumour cell lines, this compound was also toxic to normal fibroblasts WI38. Similarly, among the 1,2,3-triazole-tagged 3-deazapurines (**5c**–**e**, **7c**, **7e**), 1-*p*-(trifluoromethyl)phenyl-1,2,3-triazole in **5c** contributed to strong cytostatic potency on CFPAC-1 (IC_50_ = 7.39 µM) along with the inhibitory effect on normal fibroblasts WI38. N-7 regioisomer **7c** showed marked reduction in activity (IC_50_ = 89.24 µM) compared to its N-9 counterpart. In contrast, N-7 regioisomer of 3-deazapurine **7e** with coumarin attached to 1,2,3-triazole displayed strong antiproliferative activity (IC_50_ = 8.77 µM) on HeLa cells, whereas its N-9 analogue **5e** was devoid of any antitumor effects. From 7-deazapurine series (**8a**–**e**), compound **8a** bearing *p*-fluorophenyl-substituted 1,2,3-triazole showed the highest inhibitory effect on SW620 (IC_50_ = 8.50 µM). Introduction of bromine at C-7 of 7-deazapurine in **9c**–**e** did not have a considerable effect on cytostatic activity with respect to the corresponding congeners **8c**–**e**. Of the 5-fluoroindole (**10a**–**e**) and indole (**11a**–**e**) 1,2,3-triazole conjugates, some compounds exhibited only marginal activities. Among 1,2,3-triazole-tagged benzimidazoles (**12a**–**e**), benzimidazole **12 b** with *p*-chlorophenyl-substituted 1,2,3-triazole displayed significant and selective antiproliferative effect (IC_50_ = 0.79 µM) on A549 cells. Correlation between lipophilicity and antiproliferative effect showed that compounds **4c**, **5c**, **8a**, and **12 b** with marked cytostatic effects (IC_50_ < 10 µM) had C log*P* values in the range of 3.3–3.6. The only exception was 3-deazapurine–7-hydroxycoumarin hybrid **7e** that exhibited lower Clog *P* value of 1.5.

#### Apoptosis detection

**Figure 2.**
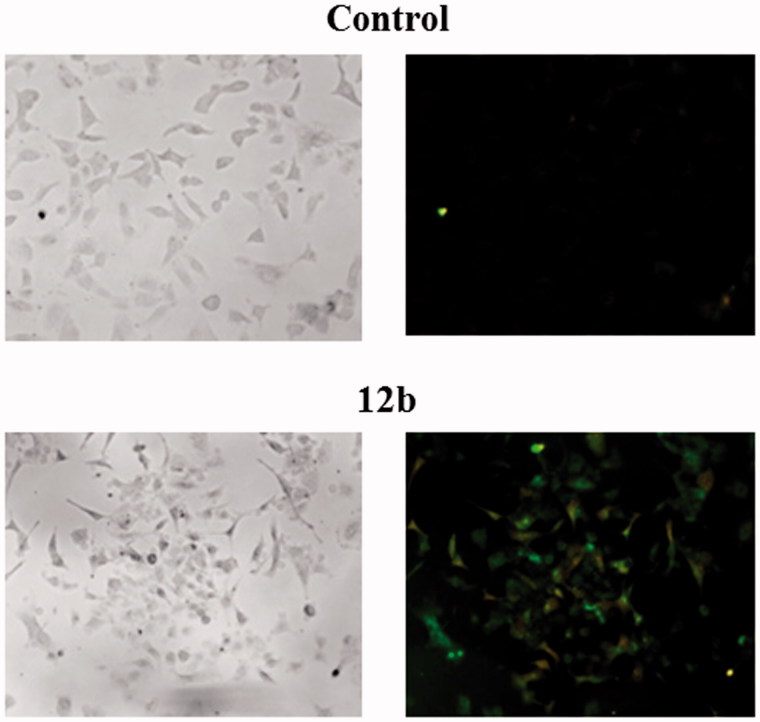
Detection of apoptosis induced by compound **12b** in non-small cell lung cancer cell line A549 using Annexin V-FITC assay. Cells were visualised by fluorescence microscope at 40× magnification before and after treatment with the concentration of 2 × IC_50_ for 48 h. PI staining was used as a nuclear marker. Shown here are bright-field images (left micrographs) and late apoptotic/primary necrotic cells (right micrographs).

Further biological evaluations of compound **12b** which was identified as a candidate were performed in order to investigate whether its antiproliferative effect in non-small cell lung cancer (A549) could be associated with induction of apoptosis. Therefore, annexin V assay was performed as previously described^38^ and obtained data are presented in Figure 2 and Table 2.

**Table 2.** Annexin V assay for apoptosis detection.

A549	Control (%)	**12b** (%)
Secondary necrotic cells	0	2.39
Early apoptotic cells	0.52	31.14
Viable cells	99.48	46.71
Late apoptotic/Primary necrotic cells	0	19.76

a The percentages of viable cells (PI−/Ann V−), early apoptotic cells (PI−/Ann V+), late apoptotic/primary necrotic cells (PI+/Ann V+) and secondary necrotic cells (PI+) after 48 h treatment with compound **12b** at 2 × IC_50_ value are shown.

Treatment with compound **12b** led to a significant reduction in the viable cell population by 55.72% concomitant with a marked increase in both, early apoptotic and late apoptotic/necrotic cell population by 30.62 and 19.76%, respectively. These findings clearly showed that compound **12b** induced apoptosis in A549 cells.

#### Western blot analysis

In addition, the Prediction of Activity Spectra for Substances (PASS)^25^ analysis was performed to reveal the probable biological activities and protein targets of **12b**. The PASS analysis predicted glycosylphosphatidylinositol specific phospholipase D1 (GPLD1) and p38 mitogen-activated protein kinase (p38 MAPK) as potential targets of **12b** (Supplementary Table S1), which was further confirmed *in vitro* by Western blot method.

**Figure 3.**
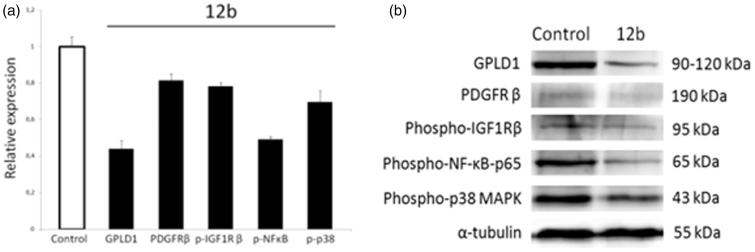
Western blot analysis of predicted protein targets of compound **12b**. Representative Western blots are shown detecting the cellular levels of selected proteins before and after treatment of A549 cells with indicated compound at 2 × IC_50_ value for 48 h. Approximate molecular weights (kDa) are indicated. Relative protein expressions, as determined by densitometric analysis of protein bands and normalised to the alpha-tubulin loading control. Two independent experiments were performed with similar results. Data are presented as mean values ± SD. Statistically significant (*p* < .05) differences in the expression levels were marked by an asterisk.

Glycosylphosphatidylinositol-specific phospholipase D1 (GPLD1) is a secreted mammalian enzyme that specifically cleaves the inositol phosphate linkage in proteins anchored by phosphatidylinositol glycans, thereby releasing the attached protein from the plasma membrane. Previous *in vitro* study has suggested that GPLD1 expression could be associated with tumour progression and malignancy^39^, and that some GPLD1 inhibitors exert growth-inhibitory activity in cancer cells at concentrations similar to those required to inhibit the enzyme^40^. Our results revealed that compound **12b** dramatically reduced the expression levels of GPLD1 protein in human non-small cell lung cancer A549, which confirmed the PASS prediction of GPLD1 protein as a potential target of **12b** (Figure 3).

**Figure 4.**
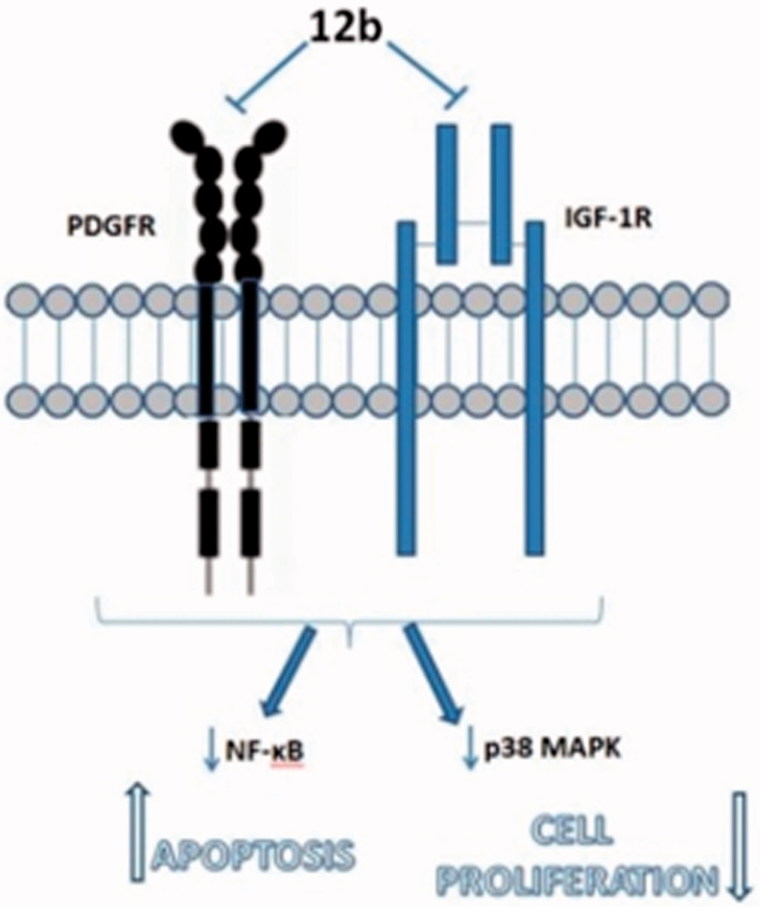
Observed antiproliferative and antiapoptotic effects of **12b** in non-small cell lung cancer cells A549 are associated with inhibition of specific plasma membrane receptors resulting in the blockade of downstream signalling propagated by p38 MAPK and NF-κb.

Importantly, this finding indicates that compound **12b** targets cell surface and suggests that **12b** could potentially modulate plasma membrane signalling to initiate a cascade of events inside the cell leading to growth inhibition and induction of apoptosis. To further substantiate this hypothesis, we examined the expression levels of two plasma membrane receptors known to promote non-small cell lung cancer cell proliferation and survival^41^ ^,^ ^42^, namely, PDGFR and IGF-1 R. We found that compound **12b** downregulated the expression of PDGFR and p-IGF-1Rβ, which supports the involvement of plasma membrane signalling in the response of A549 cells to compound **12b**. Impairment in the signalling mediated by these two receptors was further substantiated by a significant reduction in the p-NF-κB-p65 subunit expression level indicative of suppression of the activity of anti-apoptotic transcription factor NF-κB, a downstream target of both PDGFR and IGF-1 R signaling^43^ ^,^ ^44^, which could account for observed induction of apoptosis revealed by Annexin V assay. In addition, we detected a marked decline in the expression level of p-p38 MAP kinase indicative of inhibition of its activity in cells treated with **12b**. This kinase transduces signals generated by growth factors to drive cell proliferation, and its activity was previously found to be increased in human non-small cell lung cancer^45^. Thus, anti-proliferative effects of **12b** revealed by the MTT assay could be attributed to the inhibition of p38 MAPK activity, which was also in the agreement with *in silico* prediction of p38 MAPK as a putative target of **12b**. Having in mind that stimulation of PDGF receptor as well as IGF-1R both could lead to activation of p38 MAP kinase^46^ ^,^ ^47^, we may presume that inhibition of p38 kinase activity resulted from abrogation of PDGFR and IGF-1 R signalling (Figure 4).

#### Structural analysis of possible interactions of compound 12b with p38 MAPK

Based on the Western blot analysis revealing strong reduction in the expression level of p38 MAP kinase induced by compound **12b** (Figure 3), p38 MAPK was selected for further *in silico* molecular binding study. Results are supported by structural similarity to purinomimetic inhibitor, ralimetinib (LY2228820)^4^, as a selective inhibitor of p38 MAPK that has been evaluated in clinical trials in patients with advanced cancer.

The possible interactions with the p38 MAPK active site have been investigated in several steps to elucidate the most probable binding mode as described in the ‘Materials and methods’ section. The ligand docking studies were carried out using the p38 complexed with purine based inhibitor (pdb: 2GTN) and pyrazolopyridinone inhibitor (pdb: 3GFE) for DFG-in binding mode and p38 in complex with a biphenyl amide inhibitor (pdb: 3D83) to explore DFG-out binding mode. In the 2GTN structure Gyl flip was observed which might be relevant for the binding of **12b**. Binding poses of **12b** collected from different docking experiments were further refined using Embrace protocol and binding energies were estimated using MM-GBSA protocol as described in the ‘Materials and methods’ section.

**Figure 5.**
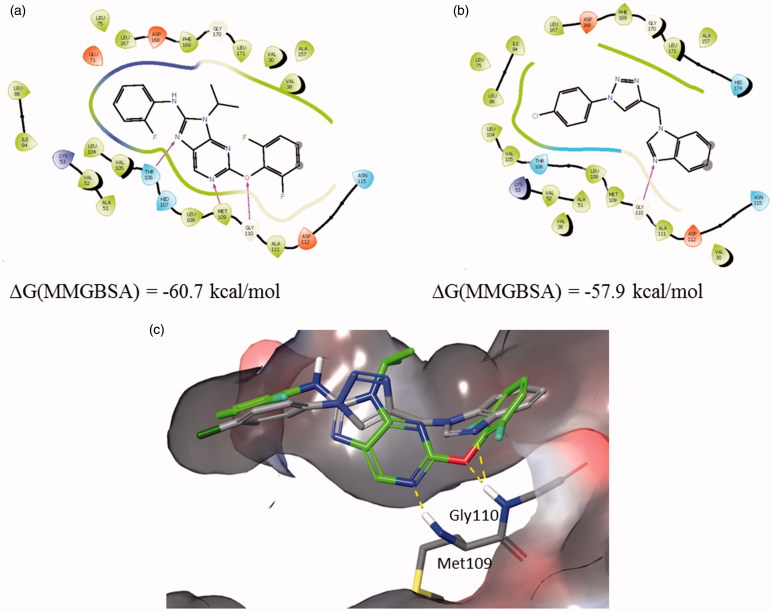
Binding interactions of (a) 2GTN X-ray structure with purine like ligand, (b) **12b** docked into 2GTN protein structure, and (c) structures from a and b superimposed within the active site pocket (green-2GTN ligand).

Complexes of **12b** with 2GTN and 3D83 are similarly stable, while the interactions with 3GFE structure were somewhat weaker. Best predicted binding pose of compound **12b** in comparison with 2GTN ligand is shown in Figure 5.

**Figure 6.**
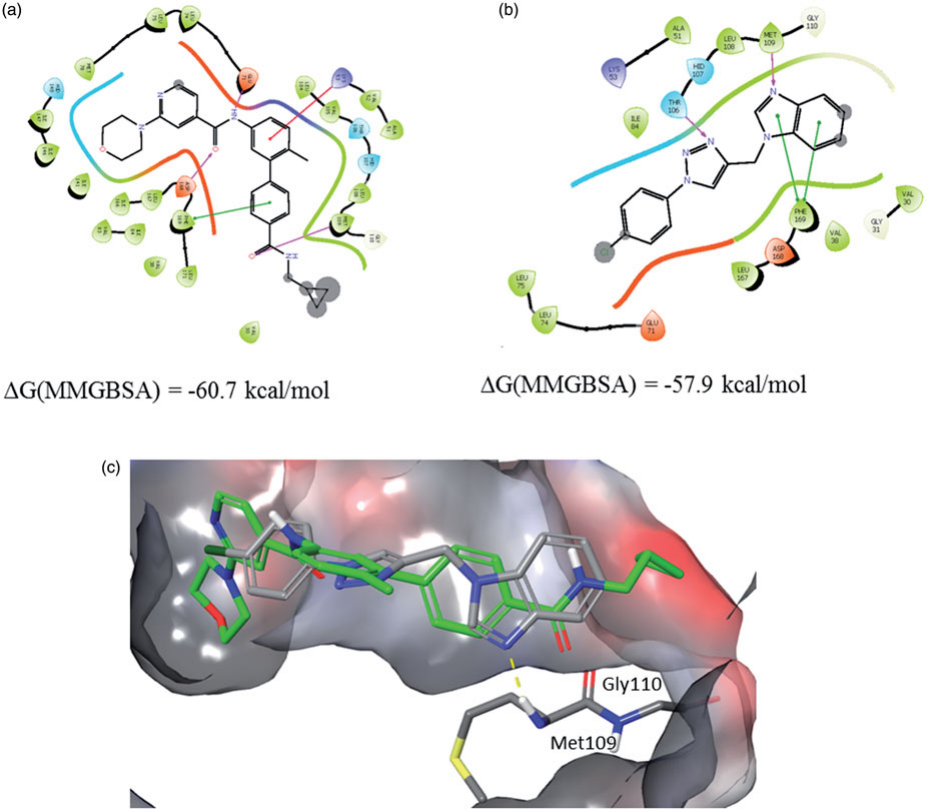
Binding interactions of (a) 3D83 X-ray structure, (b) **12b** docked into 3D83 protein structure, and (c) structures from a and b superimposed within the active site pocket (green-3D83 ligand).

The benzimidazole moiety forms an H-bond with the backbone of Gly 110 in the hinge region, while the *p*-Cl-phenyl moiety linked to the triazole is placed in the hydrophobic environment. Best predicted binding pose of compound **12b** in comparison with 3D83 ligand is shown in Figure 6. The benzimidazole moiety forms an H-bond with Met109 in the hinge region and π–π stacking with the Phe169, while the triazole moiety forms an H-bond with Thr106. Using established correlation of calculated ΔG energies and experimental binding activities^18^ ^,^ ^48^ activity range of **12b** is predicted to be below 100 nM.

## Conclusion

In summary, target halogenated purine and pseudopurines with regioselective 1,4-disubstituted 1,2,3-triazoles (**4a**, **4c**–**e**, **5c**–**e, 6e, 7c, 7e, 8a**–**e, 9c**–**e, 10a**–**e**, **11a**–**e**, and **12a**–**e**) were prepared by environmentally friendly click reactions under microwave irradiation using Cu(I) catalyst. Results of antiproliferative evaluations showed that *p*-(trifluoromethyl)-substituted 1,2,3-triazole in N-9 alkylated purine **4c** and 3-deazapurine **5c** was critical for strong albeit unselective activity in CFPAC-1 cells, whereas 1-(*p*-fluorophenyl)-1,2,3-triazole derivative of 7-deazapurine **8a** showed cytostatic effect on SW620 cells. Among purinomimetics with coumarin-substituted 1,2,3-triazoles, only N-7 regioisomer of 3-deazapurine **7e** exhibited strong cytostatic activity on HeLa cells. Notably, 1-(4-chlorophenyl)-1,2,3-triazole-tagged benzimidazole **12b** showed the most pronounced and selective inhibitory effect on A549 cells. Further *in silico* and Western blot analyses revealed that **12b** targets molecular processes at the extracellular side and inside the plasma membrane regulated by GPLD1 and growth factor receptors PDGFR and IGF-1R leading to the inhibition of cell proliferation and induction of apoptosis mediated by p38 MAP kinase and NF-κB, respectively. Based on these findings, we propose benzimidazole scaffold with aryl-substituted 1,2,3-triazole ring as new and promising chemical entity with potent cytostatic activity against A549 cells. Further structural optimisation of this compound may lead to reduction of its toxicity in normal cells without compromising its antitumor effects to develop a novel agent for treatment of non-small lung cancer cells.

## Supplementary Material

IENZ_1414807_Supplementary_Material.pdf
