# Supplementary material for: Small molecule purine and pseudopurine derivatives: synthesis, cytostatic evaluations and investigation of growth inhibitory effect in non-small cell lung cancer A549
Source: J Enzyme Inhib Med Chem. 2017 Dec 22;33(1):271–85. doi: 10.1080/14756366.2017.1414807 (PMC6009932; doi:10.1080/14756366.2017.1414807)

Small molecule purine and pseudopurine derivatives: synthesis, cytostatic evaluations and investigation of growth inhibitory effect in non-small cell lung cancer A549

Andrea Bistrović[a#], Petra Grbčić[b#], Anja Harej[b], Mirela Sedić[b]*, Sandra Kraljević-Pavelić[b], Sanja Koštrun[c], Janez Plavec[d,e,f], Damjan Makuc[d,e,] and Silvana Raić-Malić[a]*

[a]University of Zagreb, Department of Organic Chemistry, Faculty of Chemical Engineering and Technology, Marulićev trg 20, HR-10000 Zagreb, Croatia; [b]University of Rijeka, Department of Biotechnology, Center for High-Throughput Technologies, Ulica Radmile Matejčić 2, HR-51000 Rijeka, Croatia, [c]Chemistry Department, Fidelta Ltd., Prilaz baruna Filipovića 29, HR-10000 Zagreb, Croatia, [d]Slovenian NMR Centre, National Institute of Chemistry, Hajdrihova 19, SI-1000 Ljubljana, Slovenia; [e]En-FIST Centre if Excellence, Trg Osvobodilne fronte 13, SI-1000 Ljubljana, Slovenia; [f]University of Ljubljana, Faculty of Chemistry and Chemical Technology, Večna pot 113, SI-1000 Ljubljana, Slovenia

*Corresponding Authors:

Tel.: 00385-1-4597-213. Fax.: 00385-1-4597-224. E-mail: sraic@fkit.hr;

Tel.: 00385-51-584-574. Fax: 00385-51-584-599. E-mail: msedic@biotech.uniri.hr

#Equally contributed

Content	Pages
1. *In silico* analysis of biological targets by PASS for compound **12b**	S2
4. NMR spectra of novel compounds	S3-S33

1. *In silico* analysis of biological targets for compound 12b

Table S1. Selected biological targets as predicted by PASS[a] for compound **12b**

Biological targets	Pa	Pi
Glycosylphosphatidylinositol phospholipase D inhibitor	0.615	0.060
MAP kinase kinase inhibitor	0.331	0.003

[a]Activity spectrum predicted by PASS is presented by the list of activities with the probabilities "to be active" (Pa) and "to be inactive" (Pi) calculated for each activity. Increased Pa and decreased Pi, the more probable is predicted activity. The list is arranged in descending order of Pa-Pi; therefore, more probable activities are at the top of the list.

2. NMR spectra of novel compounds

Fig. S2 a) ^{1}H NMR and b) ^{13}C NMR of compd. **2b**

a)

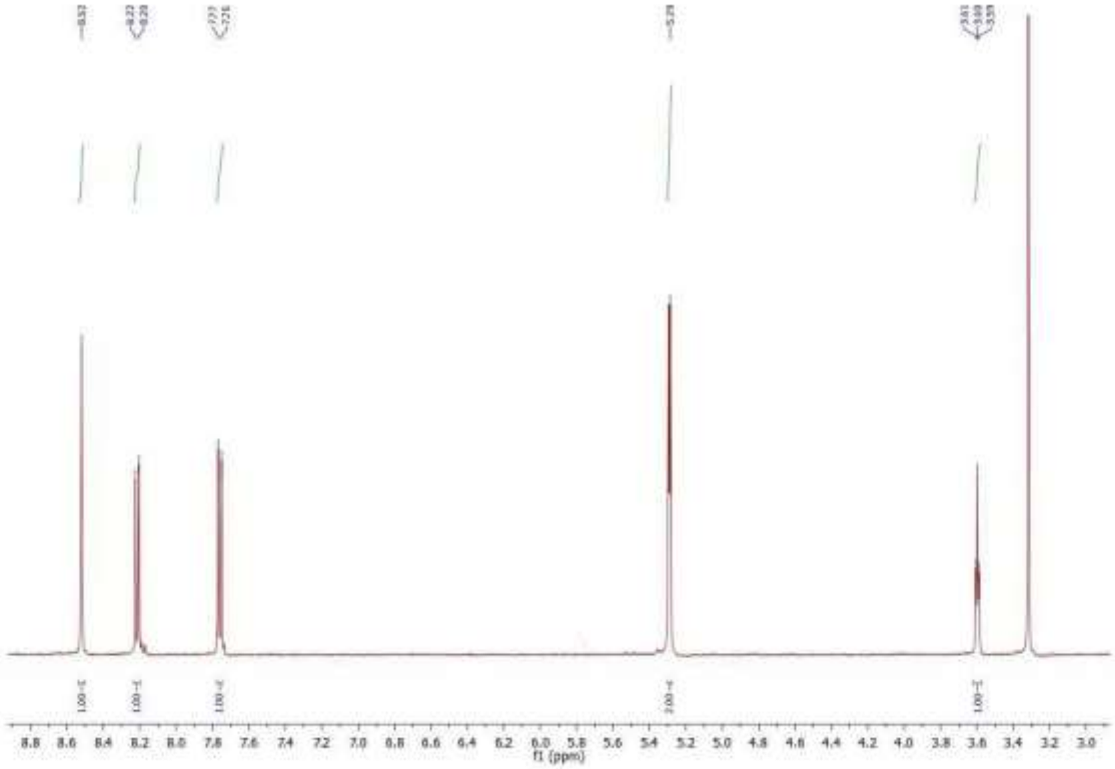

b)

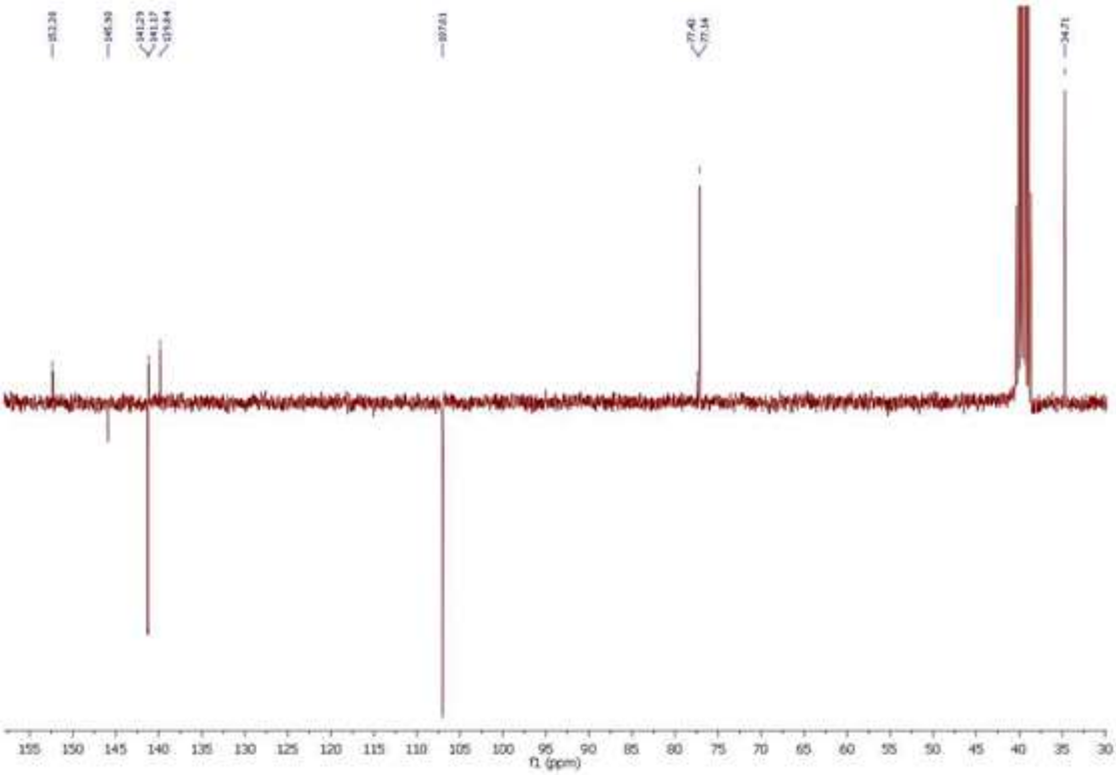

Fig. S3 a) ^{1}H NMR and b) ^{13}C NMR of compd. **2d**

a)

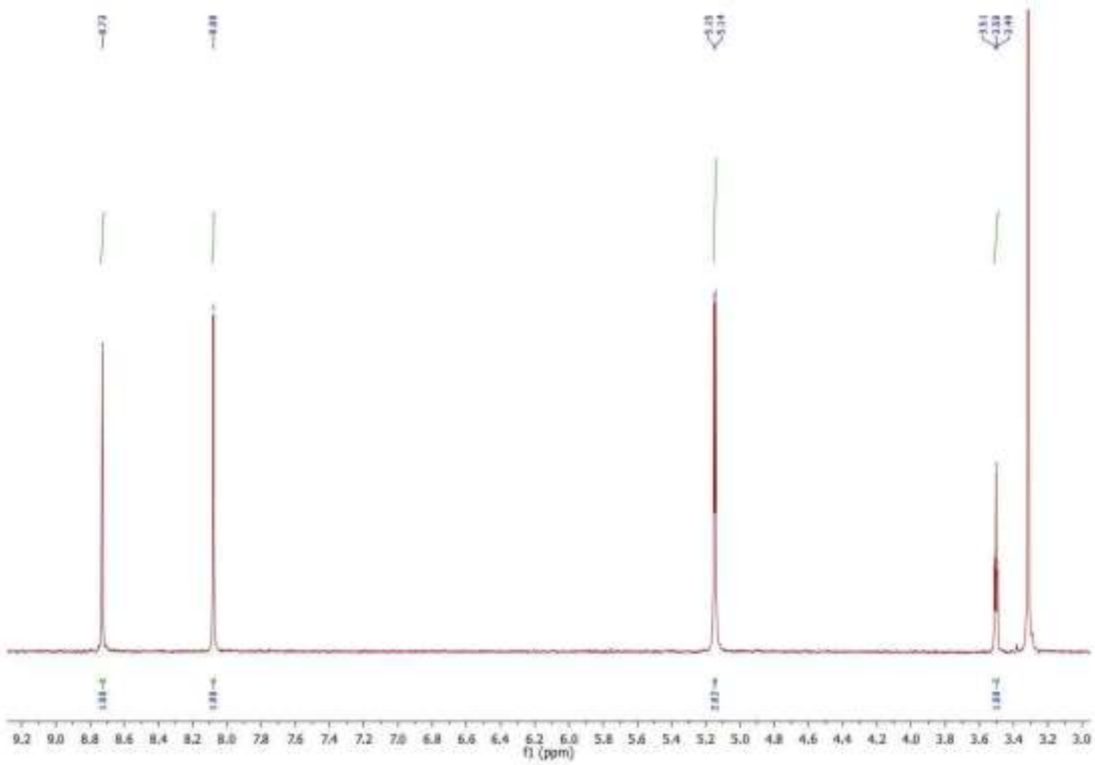

b)

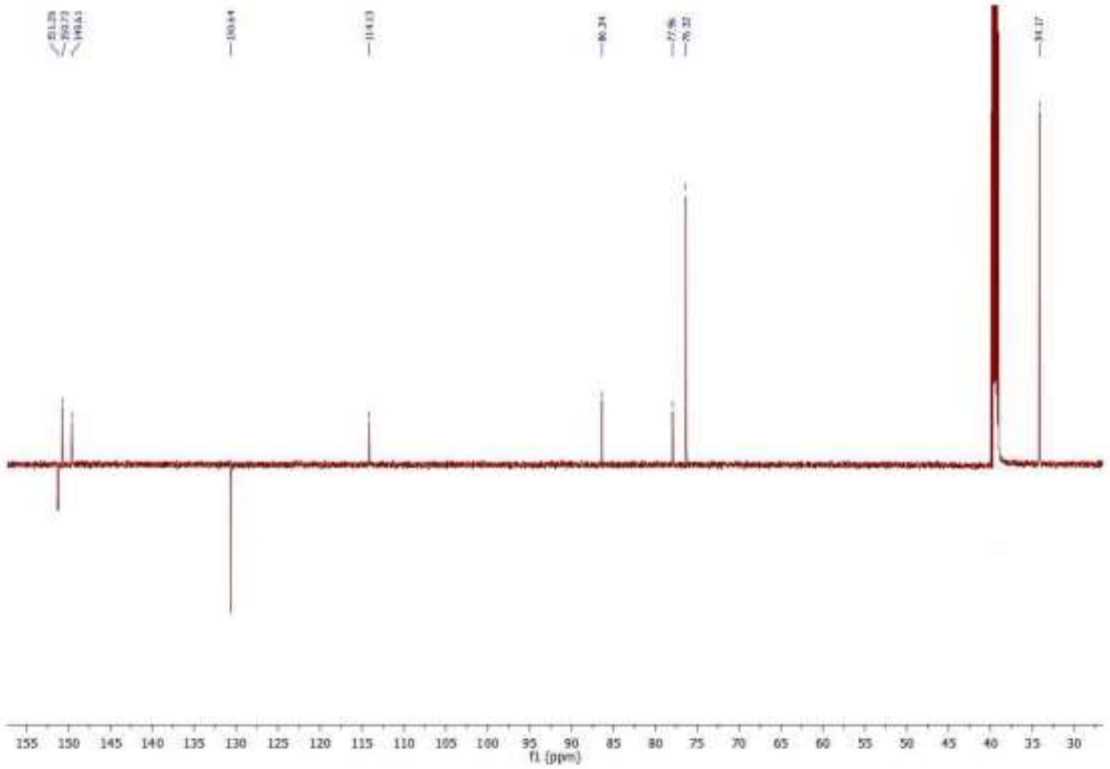

Fig. S4 a) ^{1}H NMR and b) ^{13}C NMR of compd. **3b**

a)

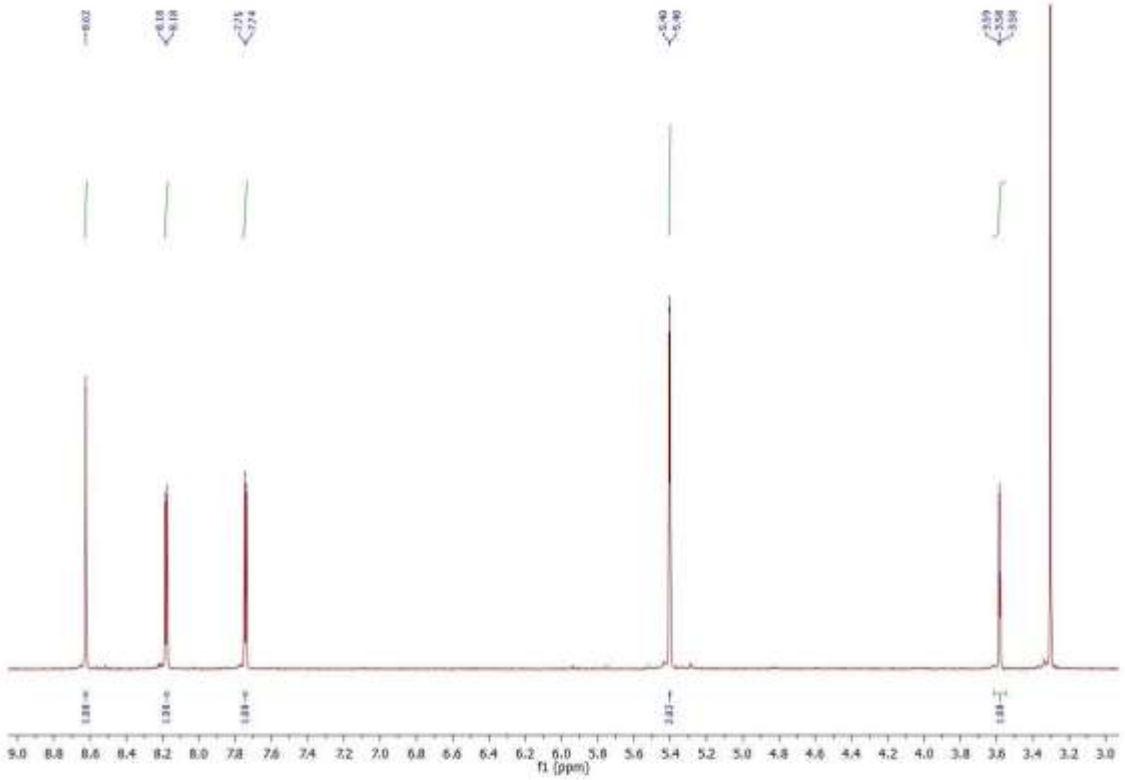

b)

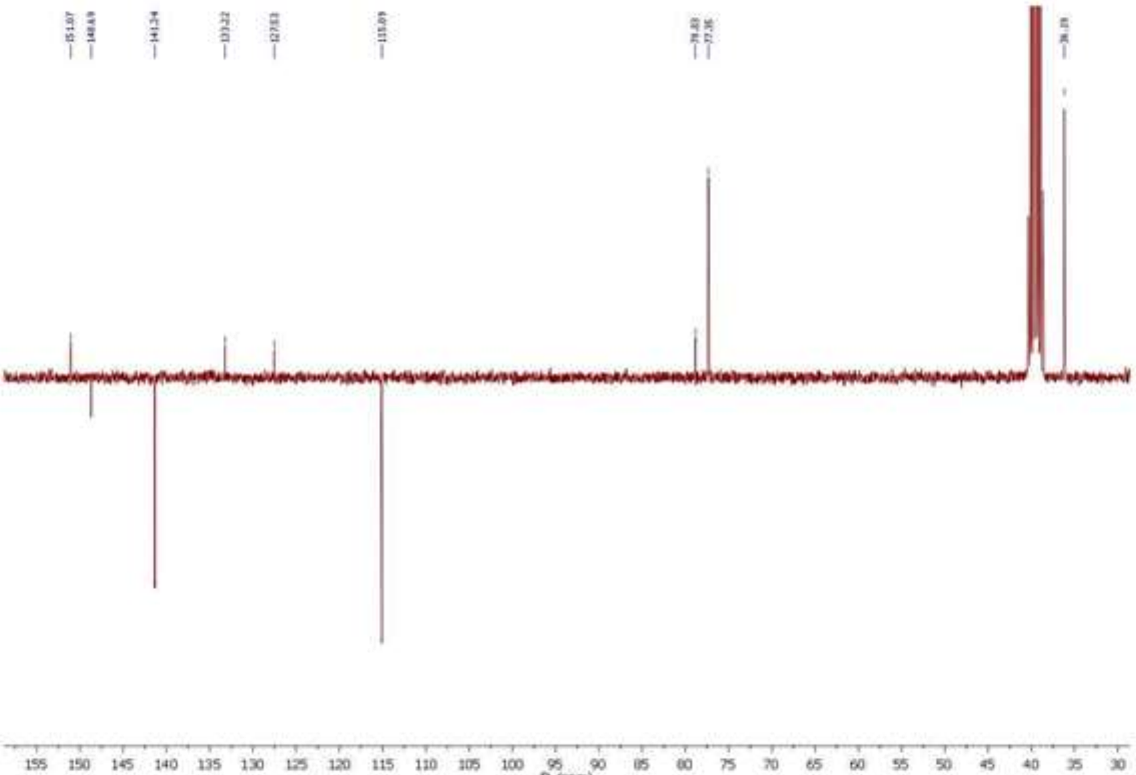

Fig. S5 a) ^{1}H NMR and b) ^{13}C NMR of compd. **4a**

a)

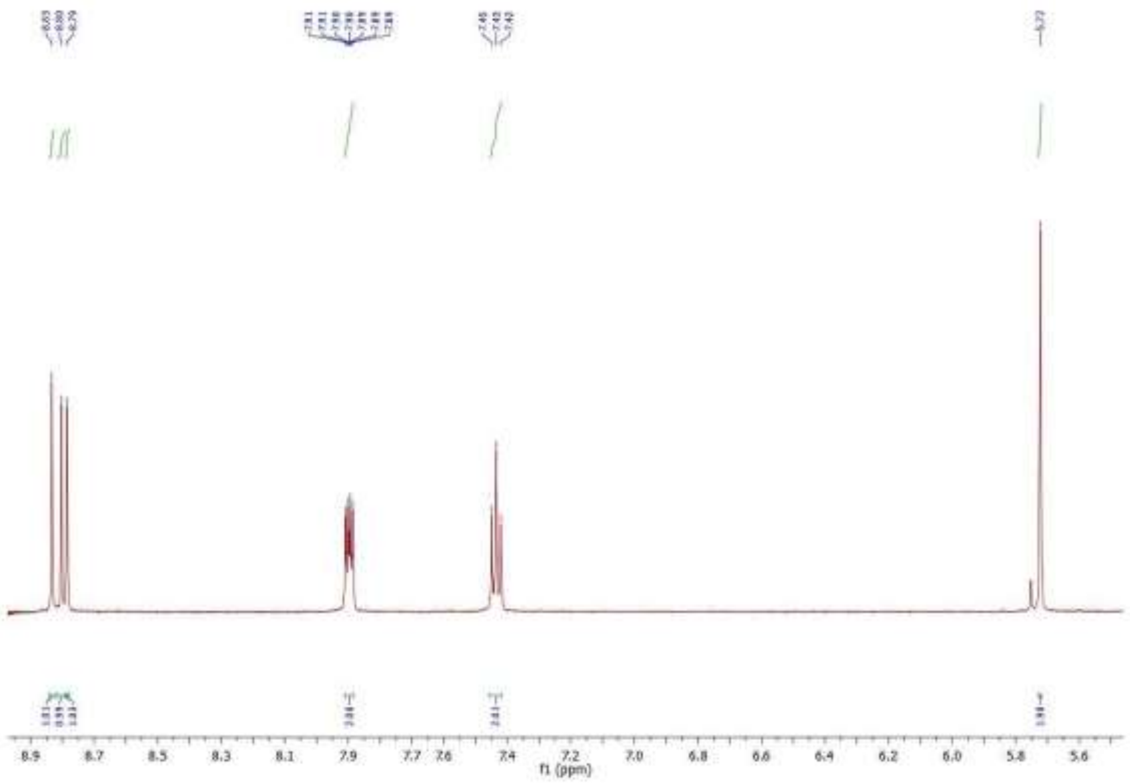

b)

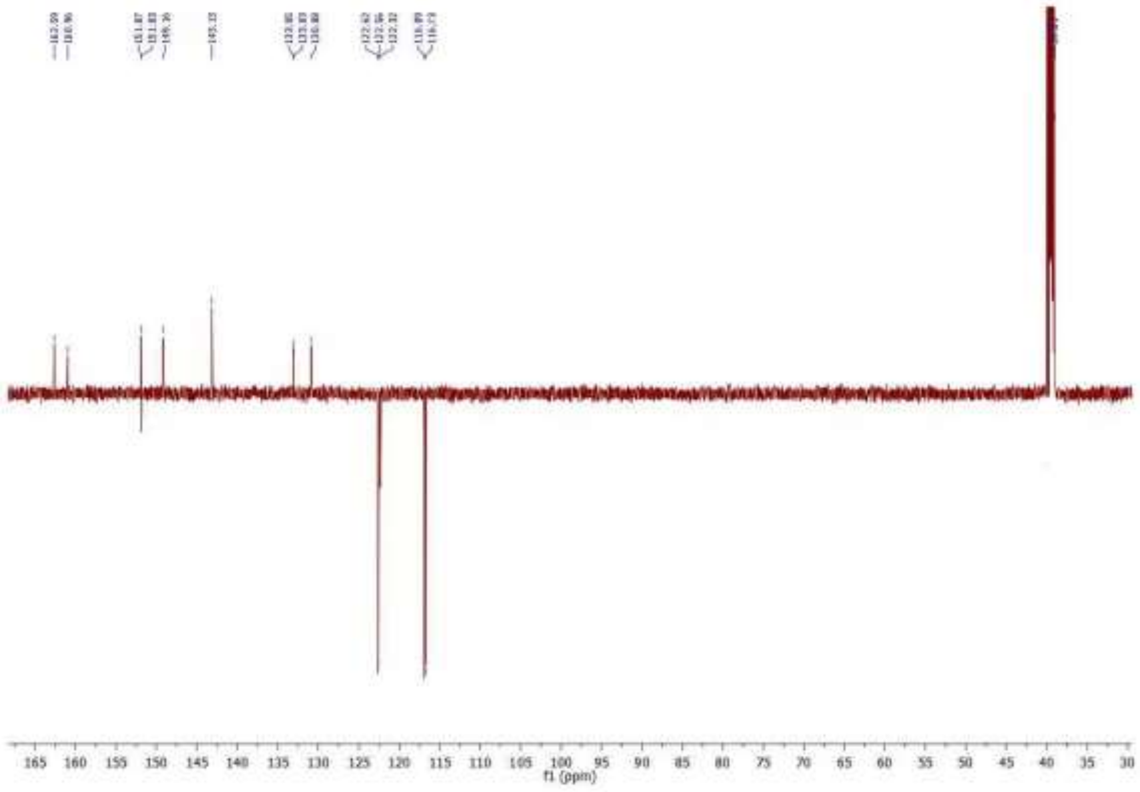

Fig. S6 a) ^{1}H NMR and b) ^{13}C NMR of compd. **4c**

a)

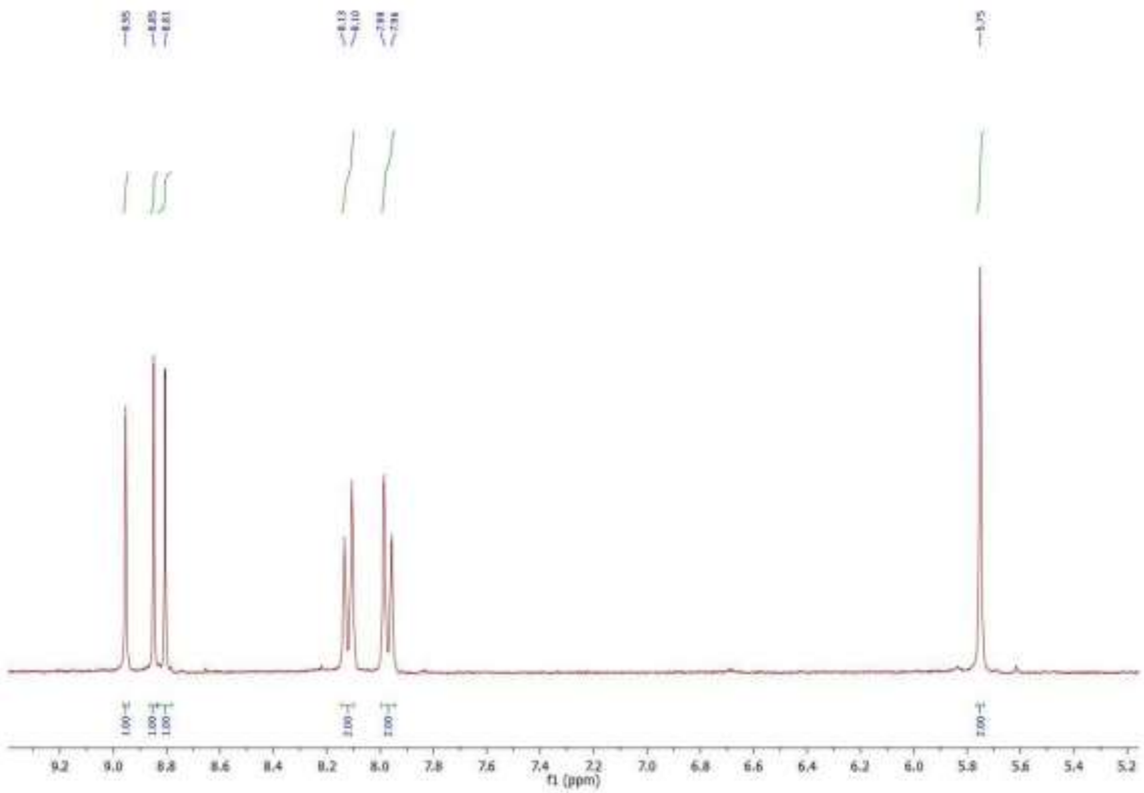

b)

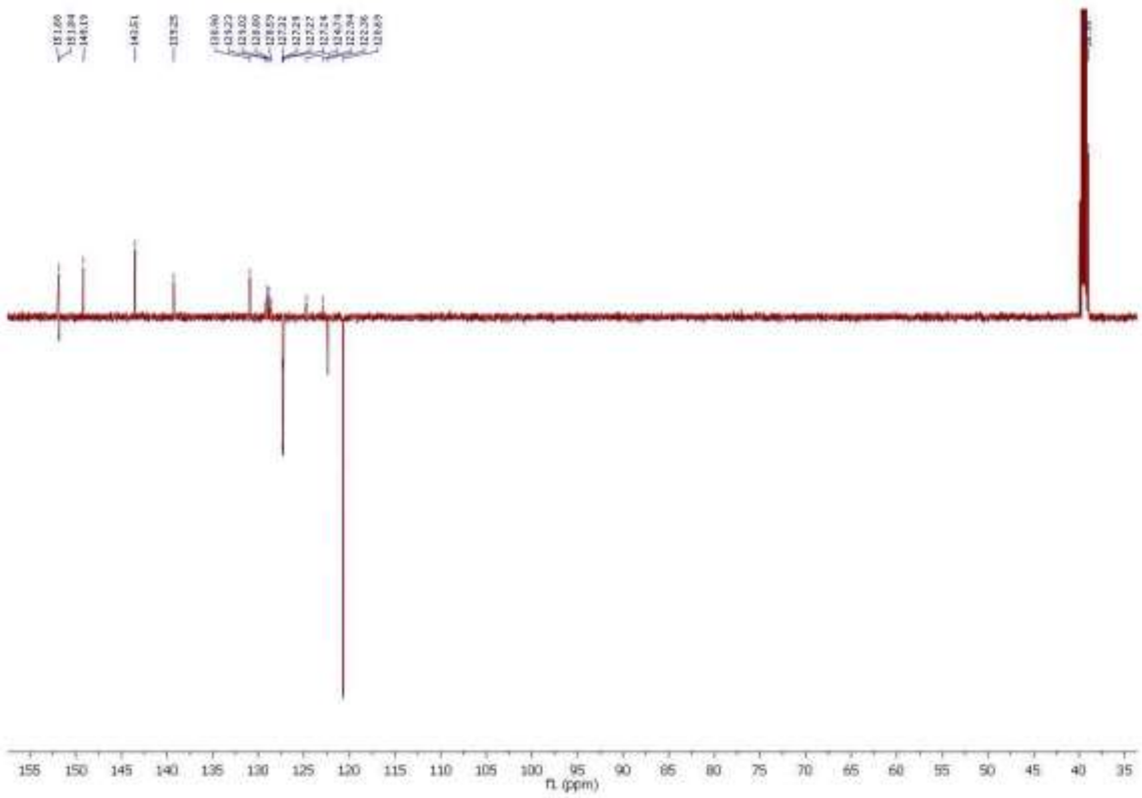

Fig. S7 a) ^{1}H NMR and b) ^{13}C NMR of compd. **4d**

a)

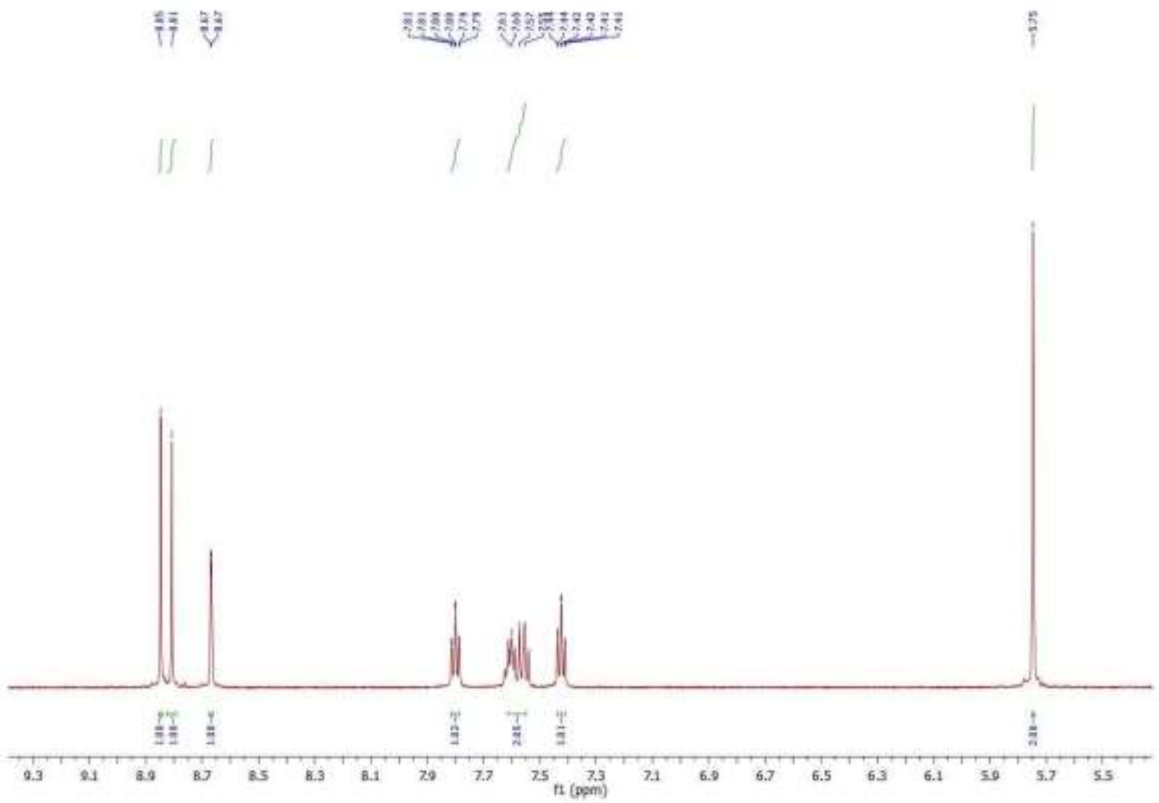

b)

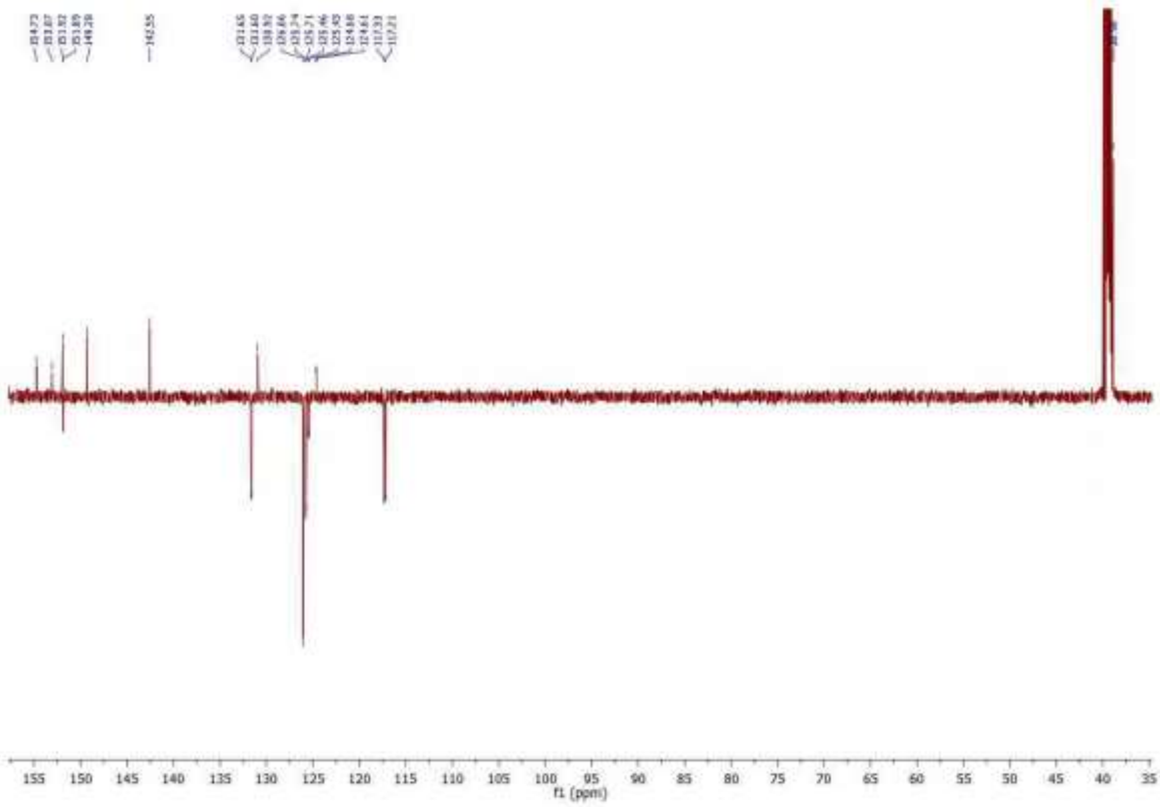

Fig. S8 a) ^{1}H NMR and b) ^{13}C NMR of compd. **4e**

a)

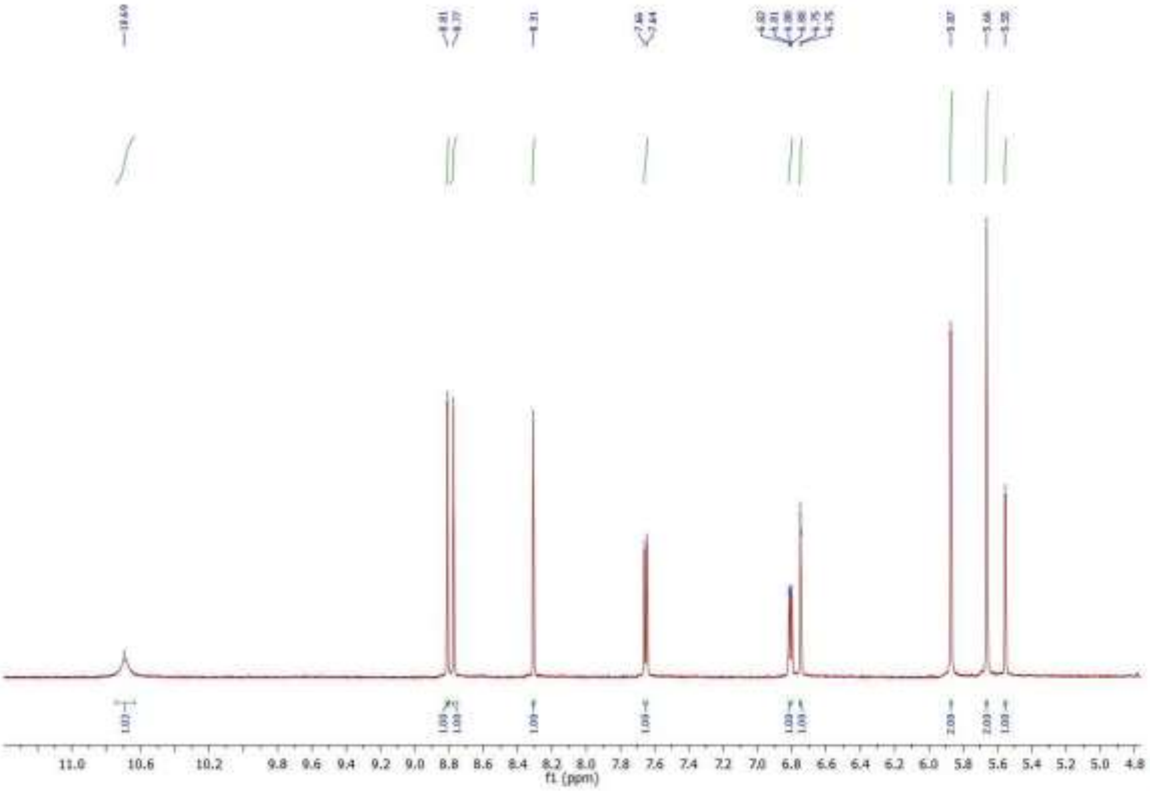

b)

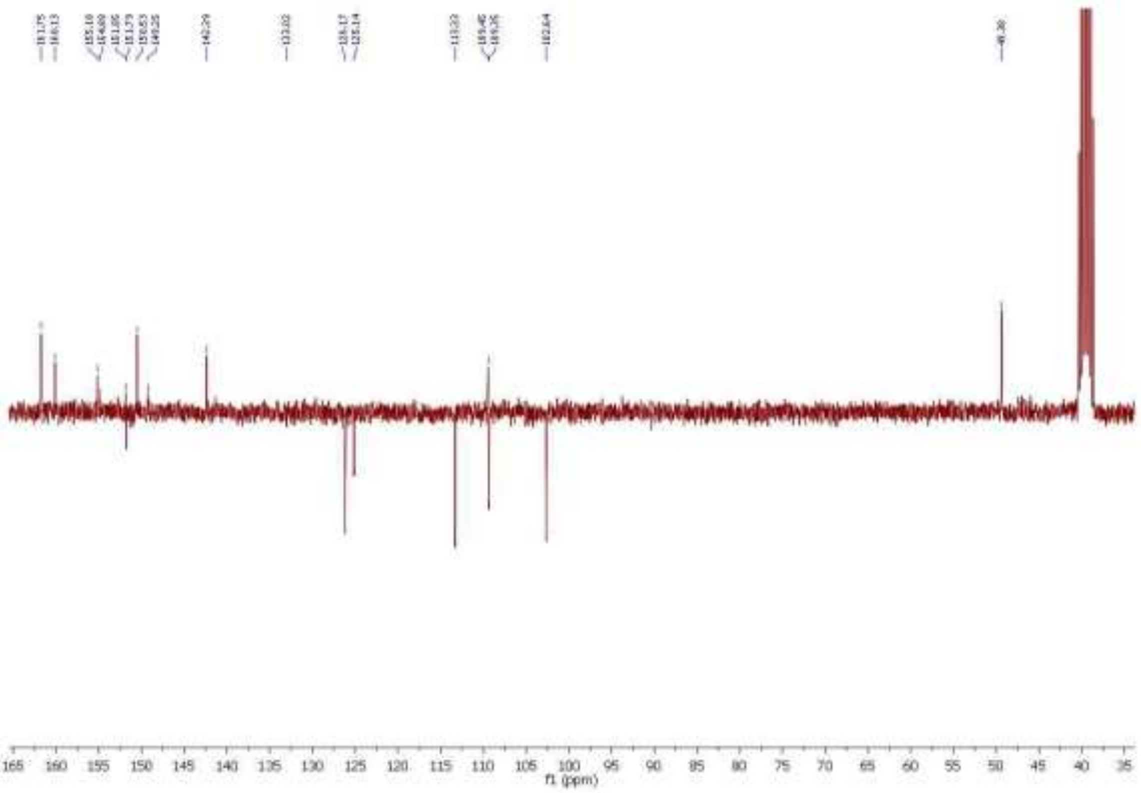

Fig. S9 a) ^{1}H NMR and b) ^{13}C NMR of compd. **5c**

a)

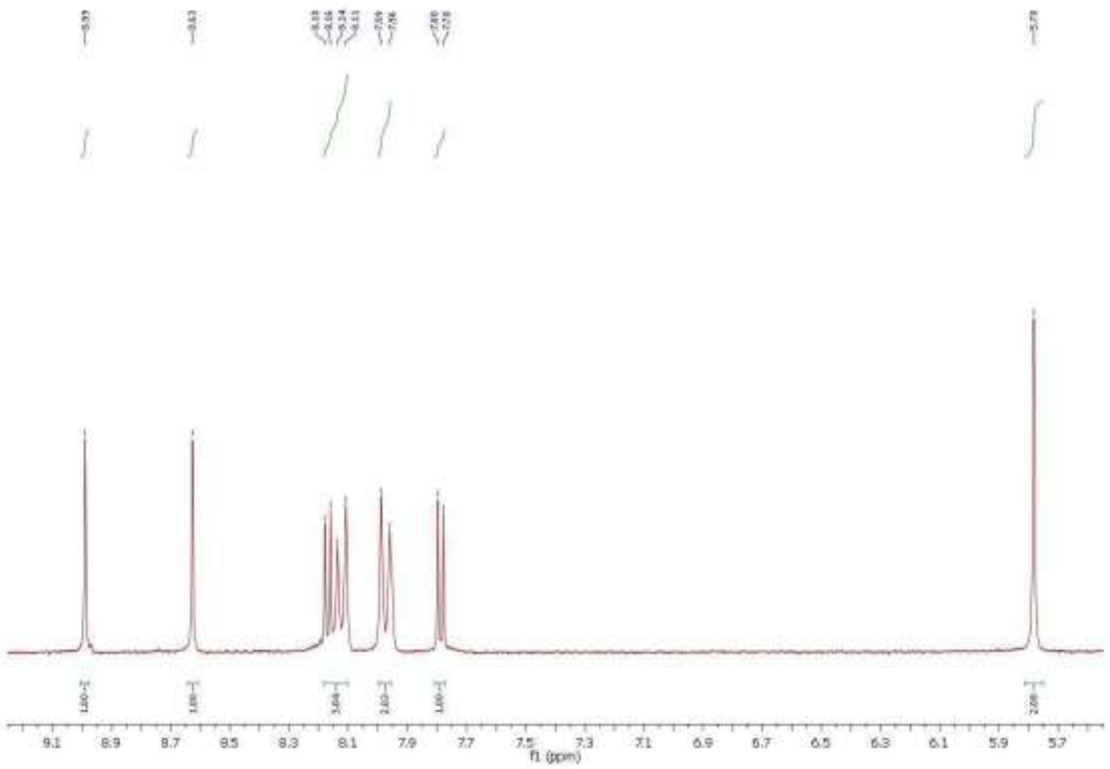

b)

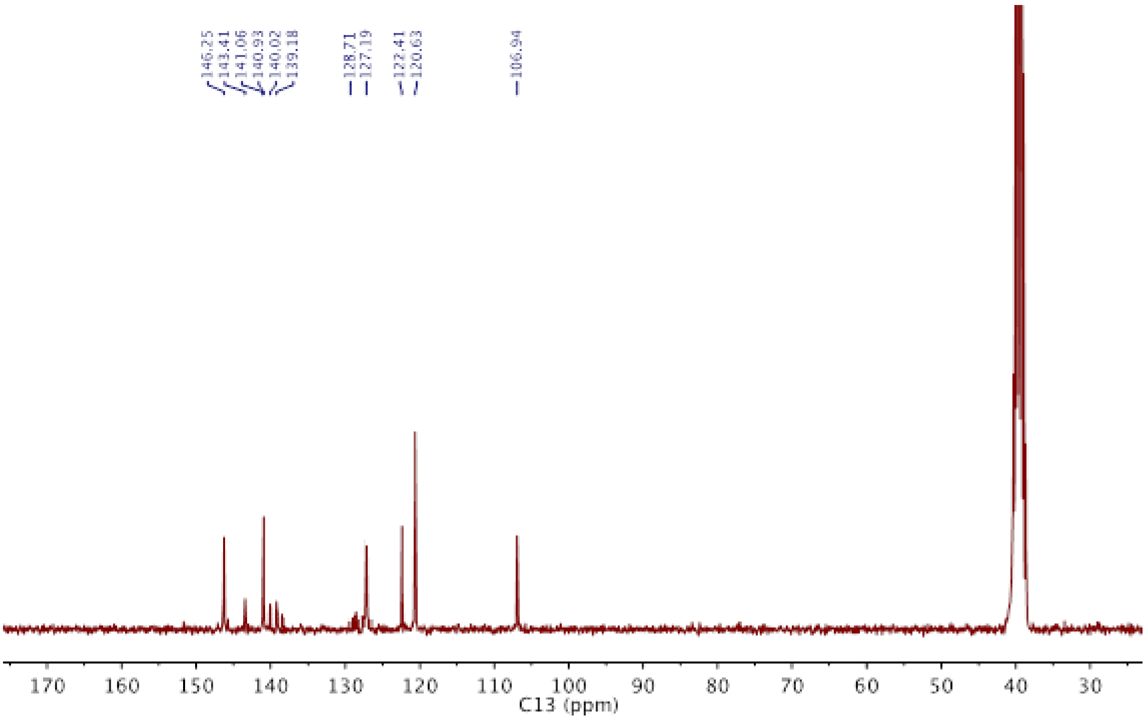

Fig. S10 a) ^{1}H NMR and b) ^{13}C NMR of compd. **5d**

a)

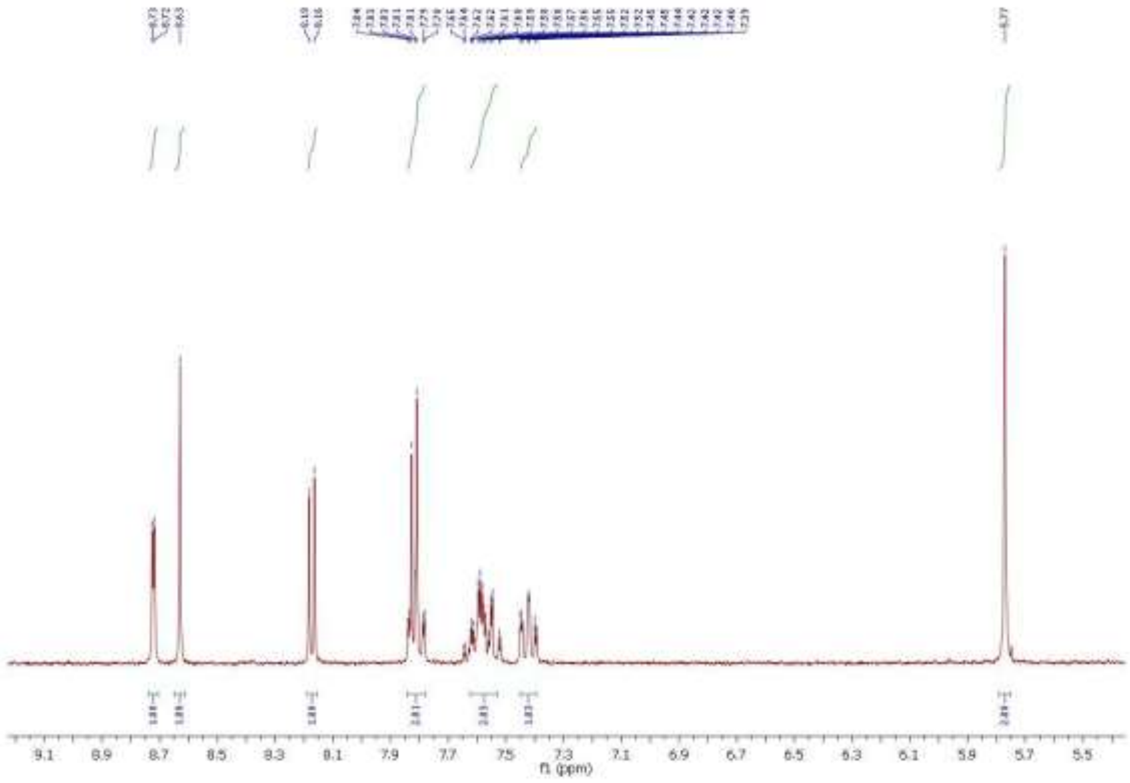

b)

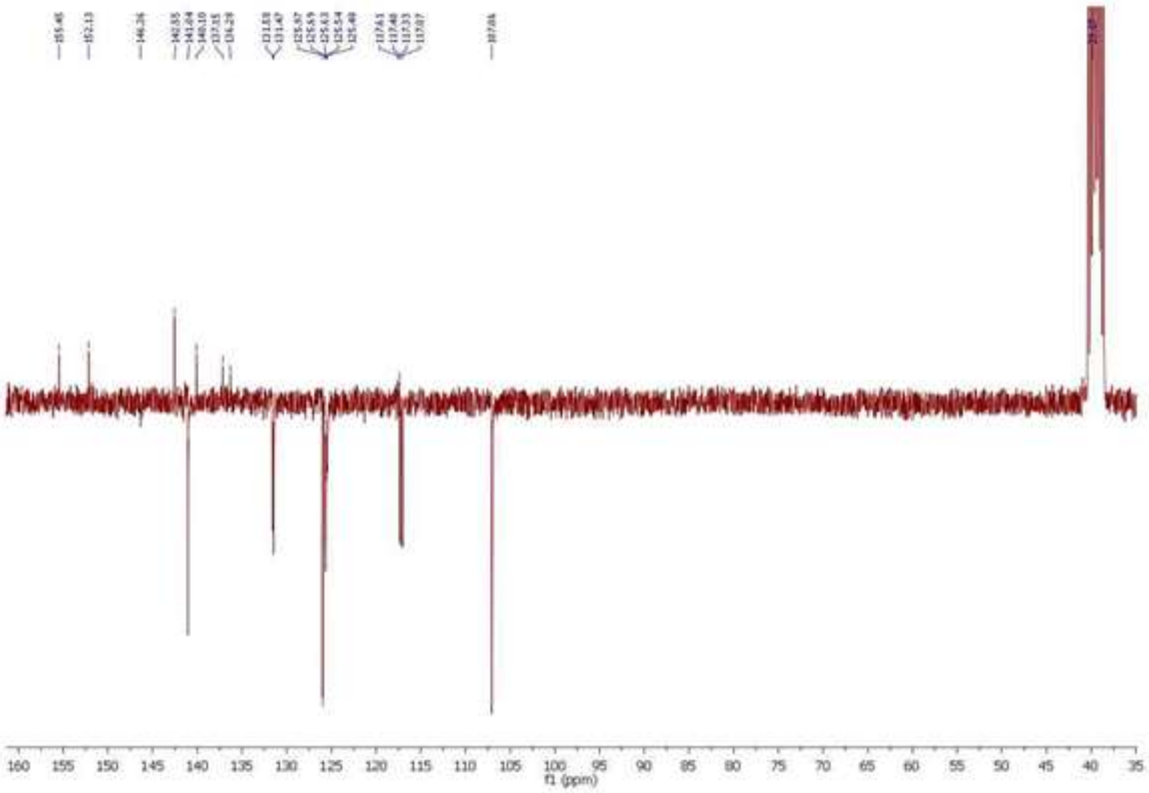

Fig. S11 a) ^{1}H NMR and b) ^{13}C NMR of compd. **5e**

a)

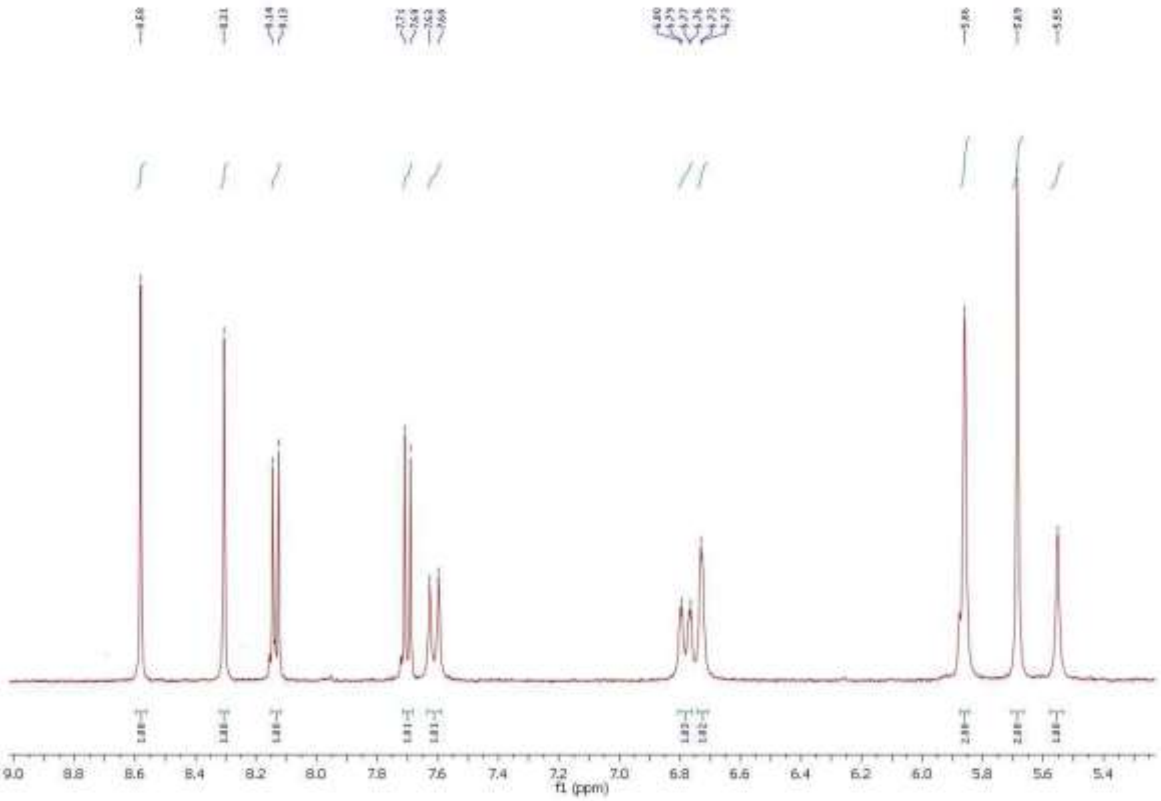

b)

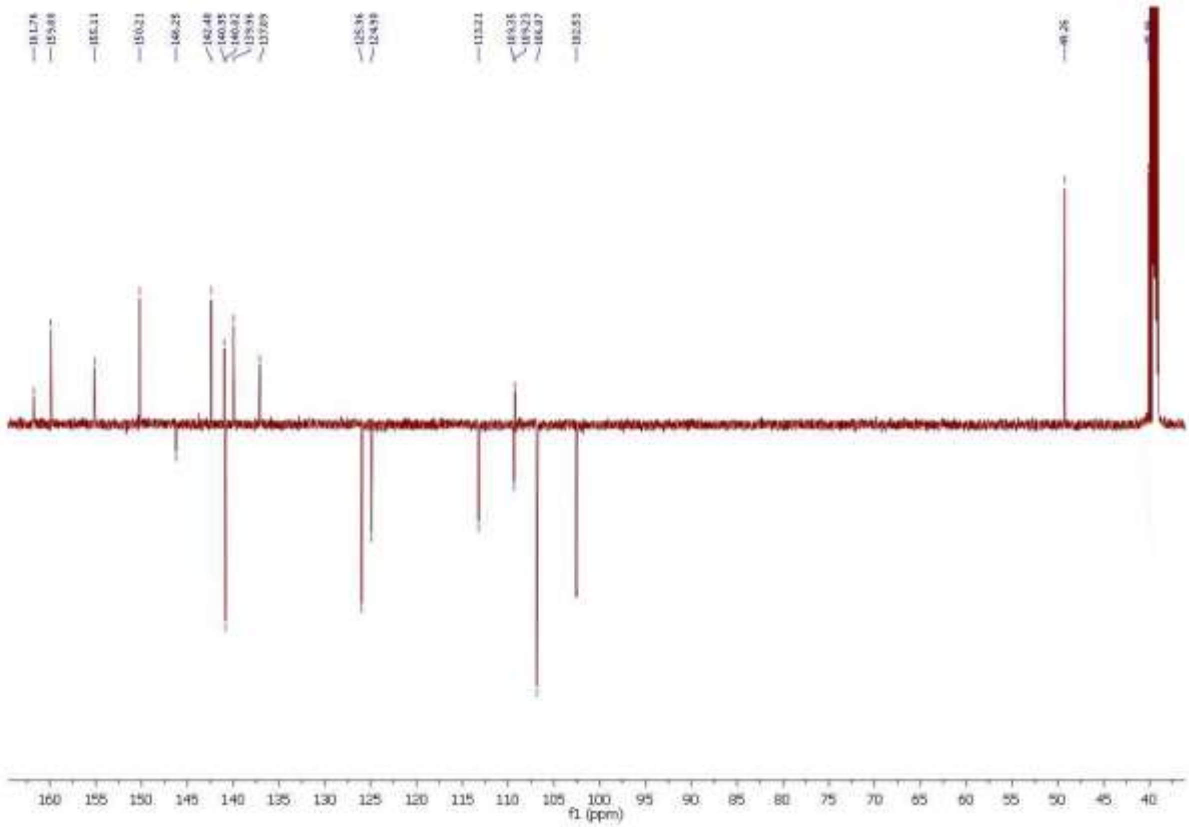

Fig. S12 a) ^{1}H-^{13}C HSQC NMR and b) ^{1}H-^{13}C HMBC NMR of compd. **6e**

a)

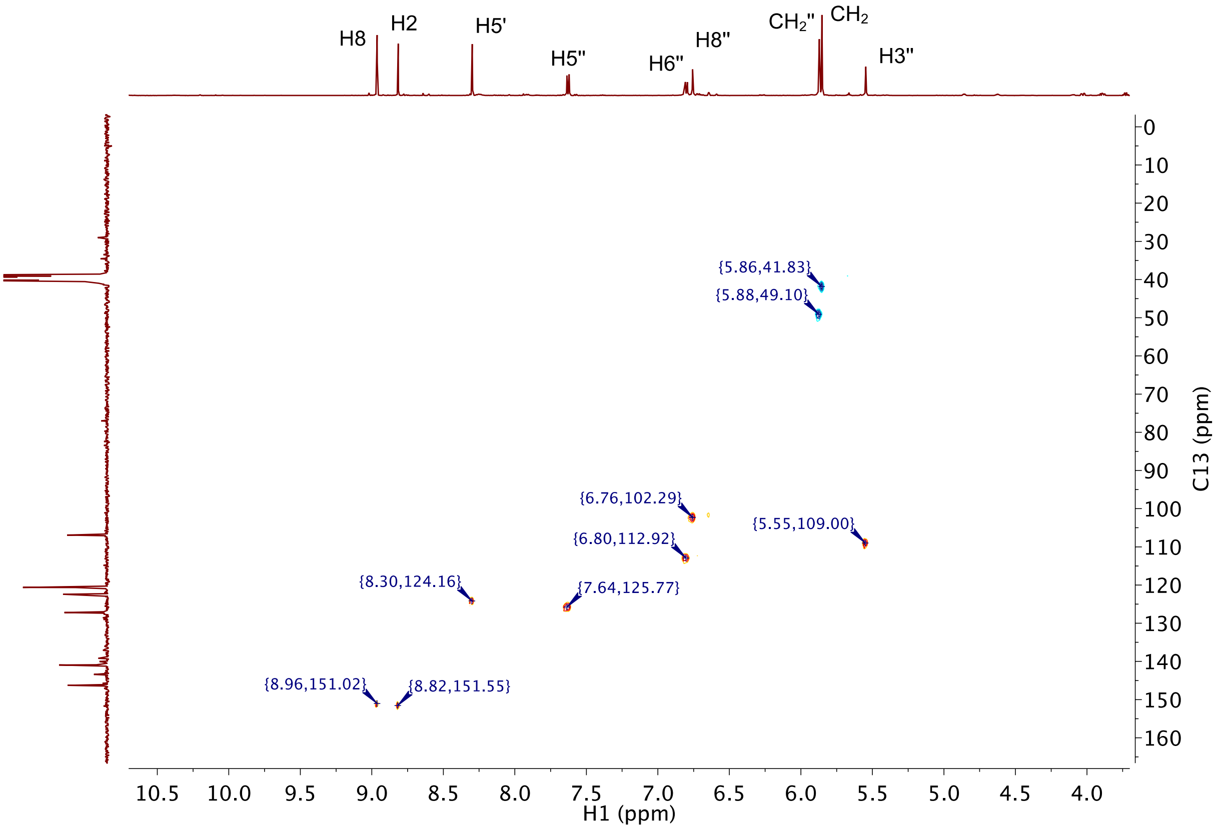

b)

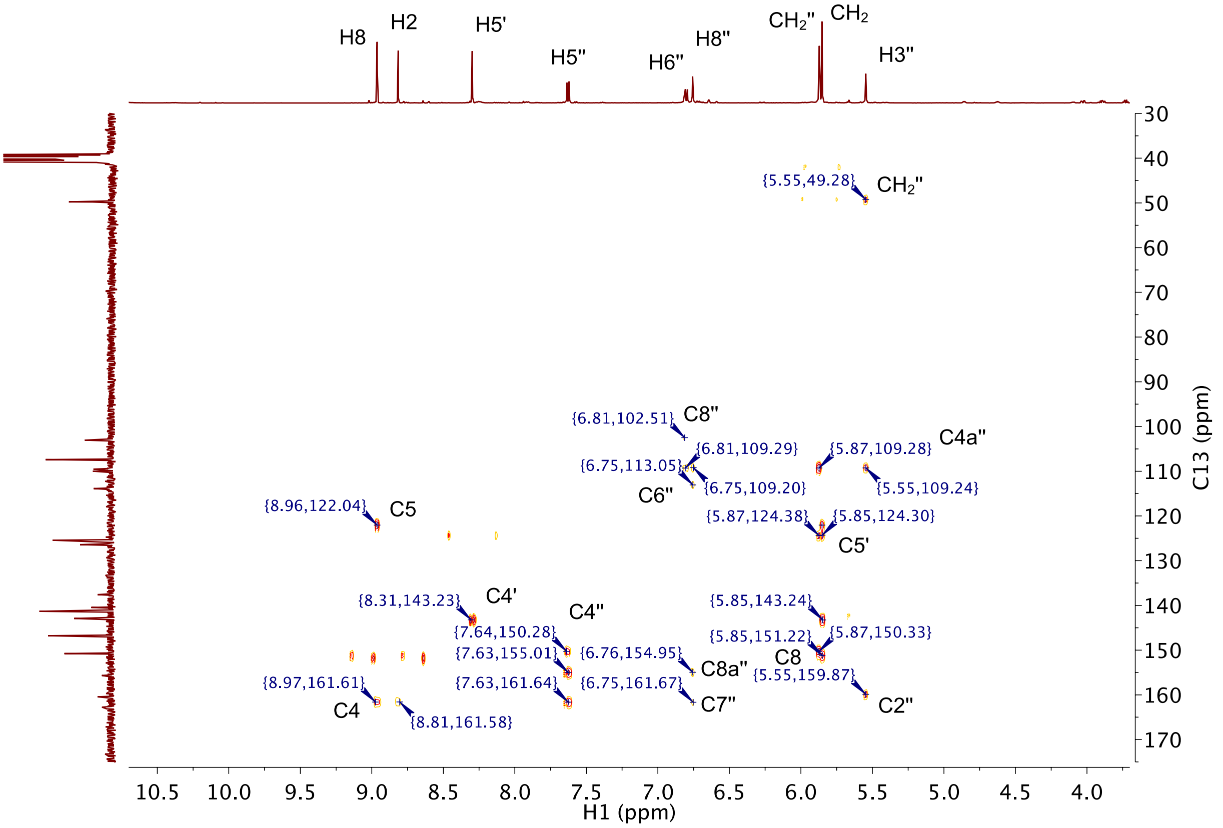

Fig. S13 a) ^{1}H NMR and b) ^{13}C NMR of compd. **7c**

a)

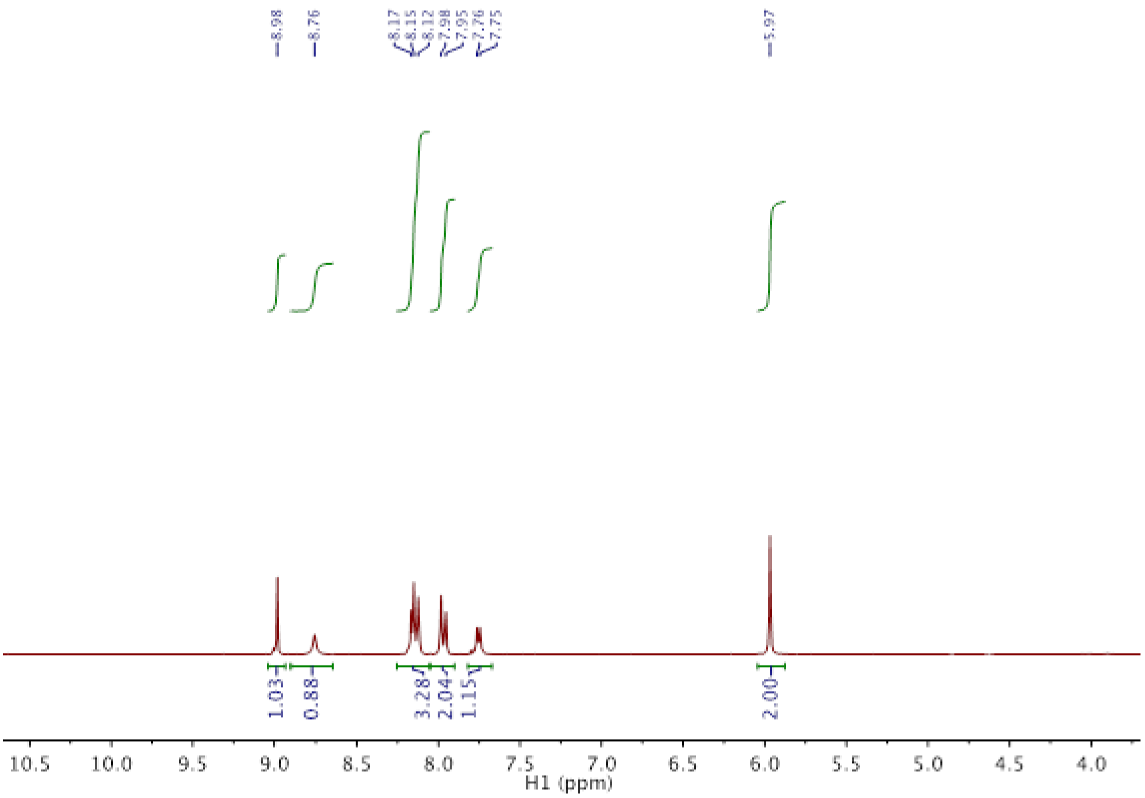

b)

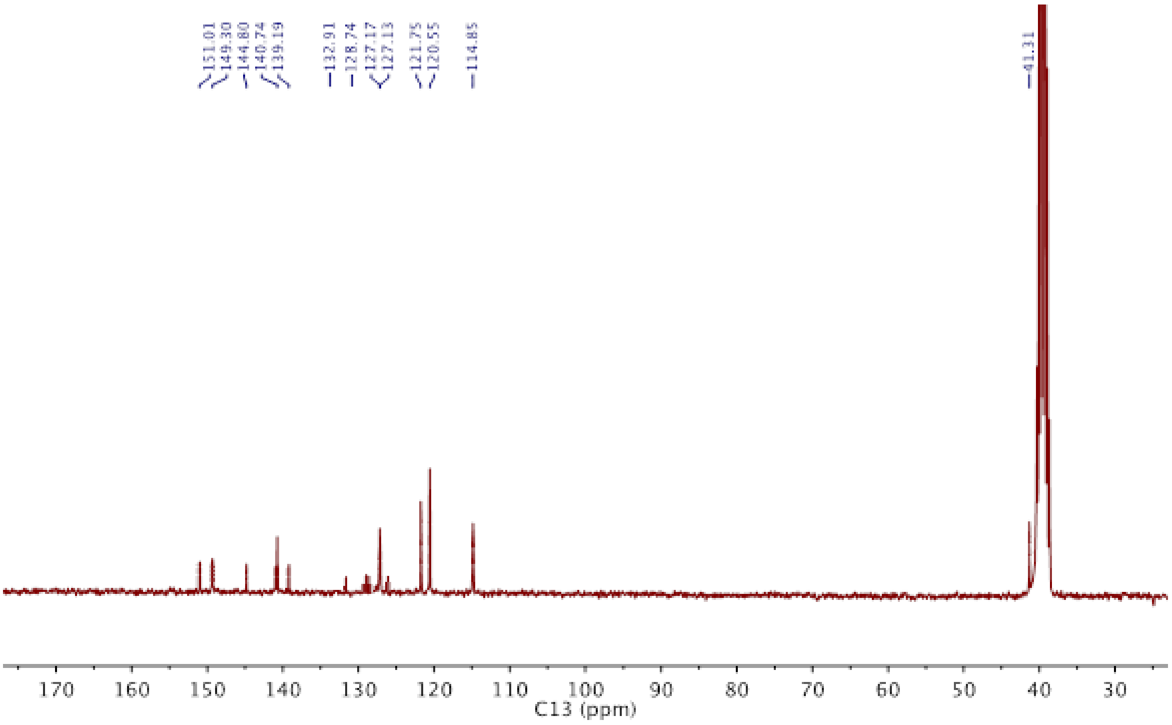

Fig. S14 a) ^{1}H NMR and b) ^{13}C NMR of compd. **7e**

a)

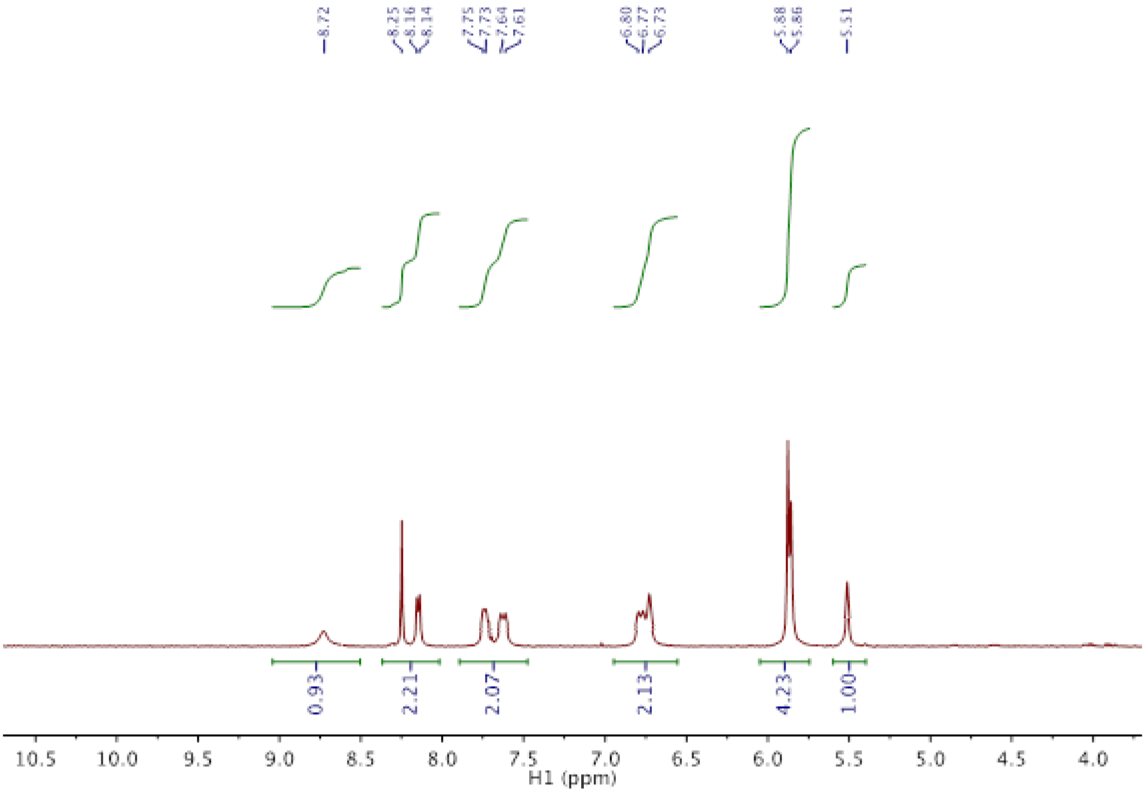

b)

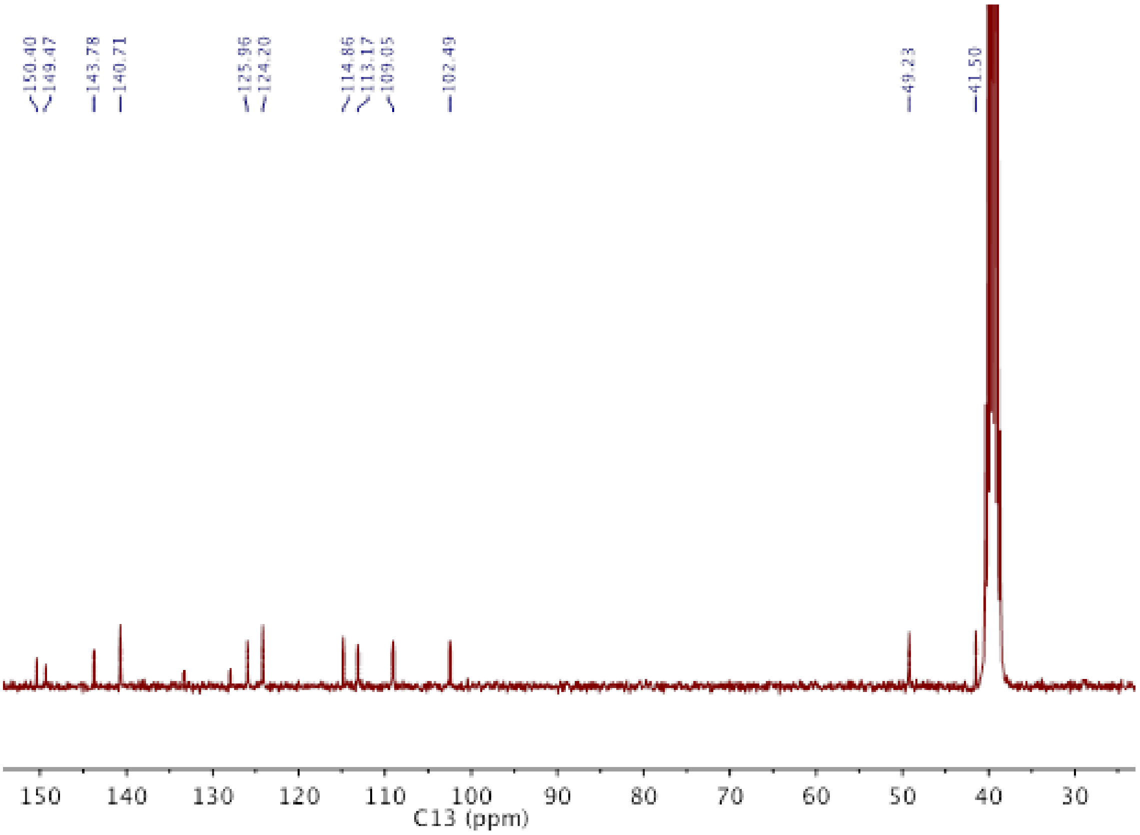

Fig. S15 a) ^{1}H NMR and b) ^{13}C NMR of compd. **8a**

a)

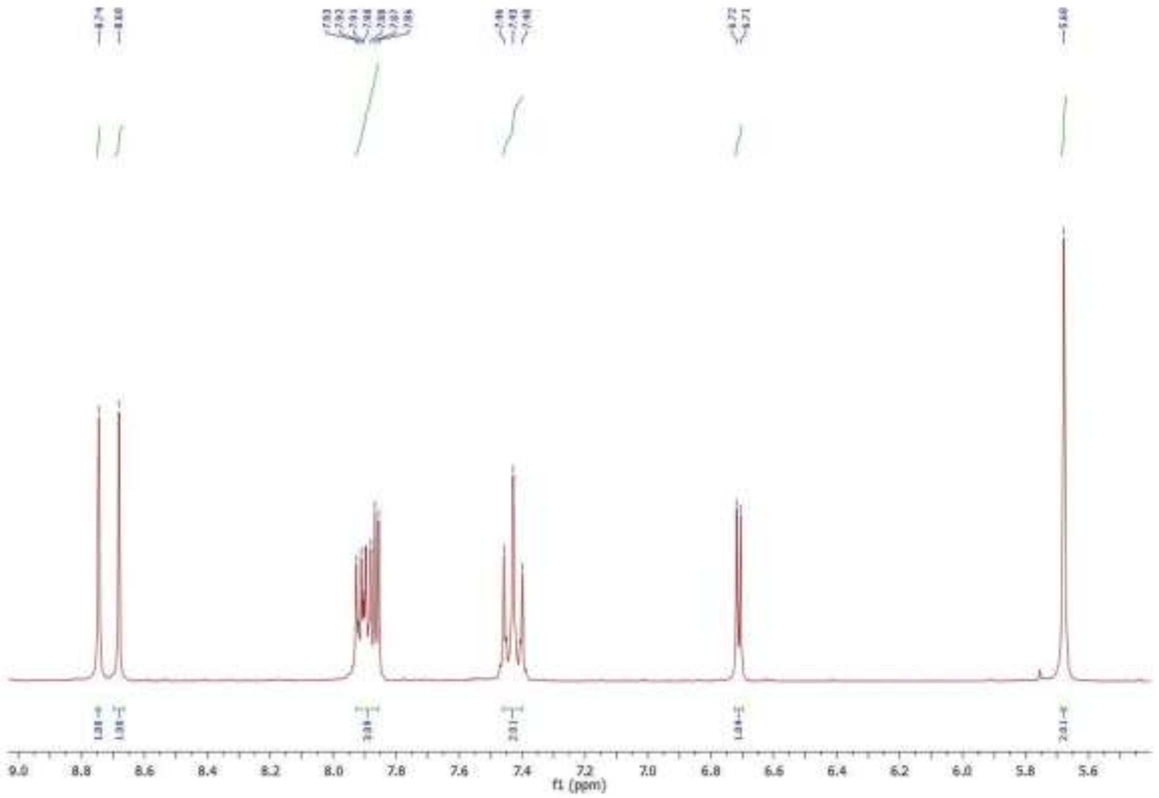

b)

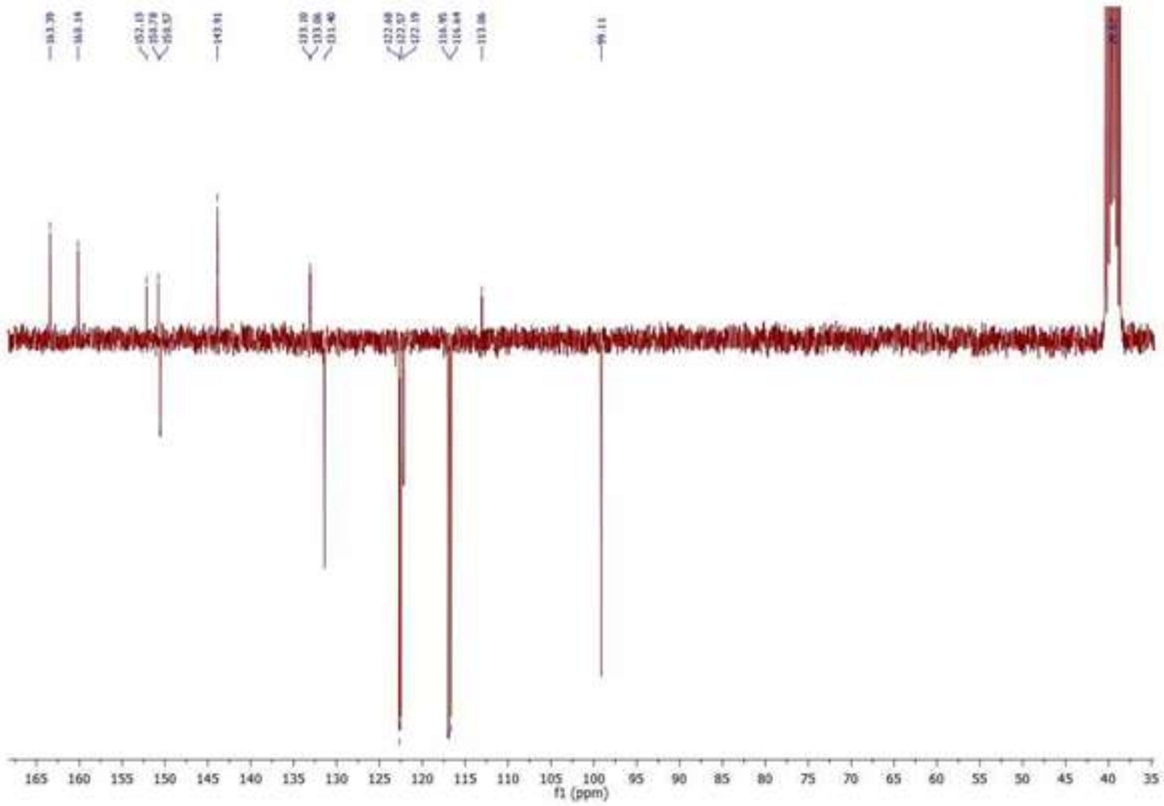

Fig. S16 a) ^{1}H NMR and b) ^{13}C NMR of compd. **8b**

a)

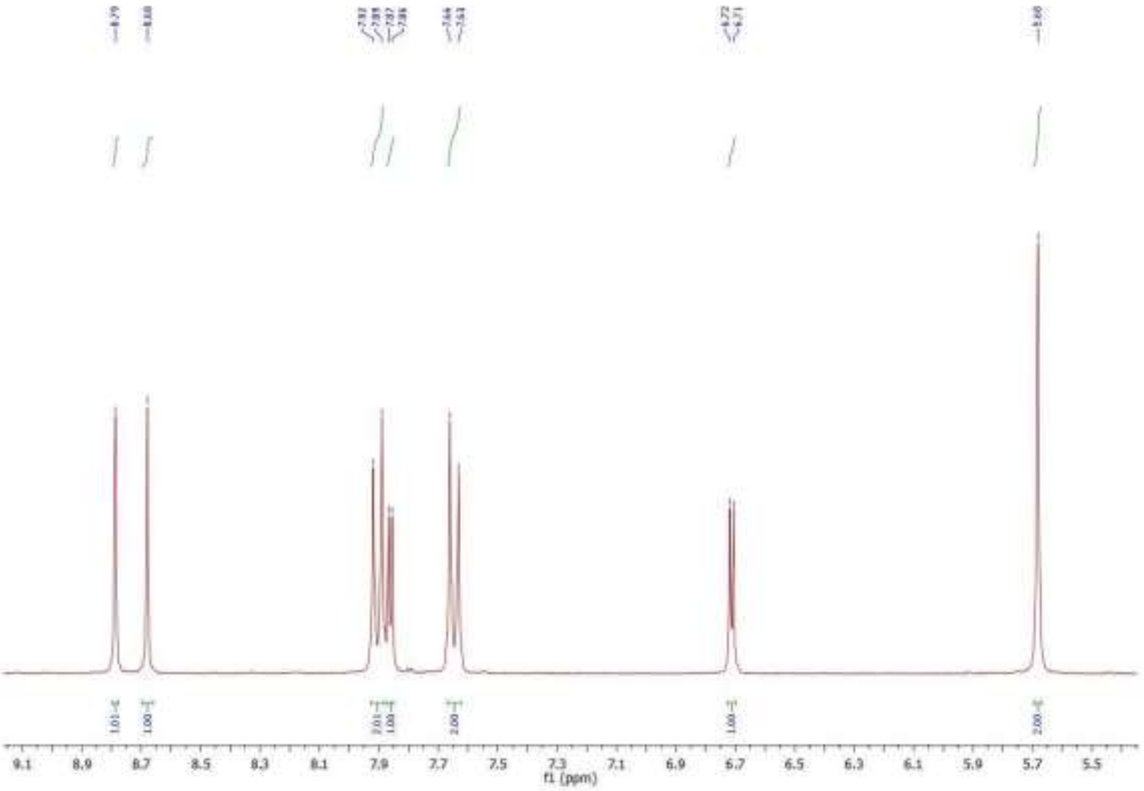

b)

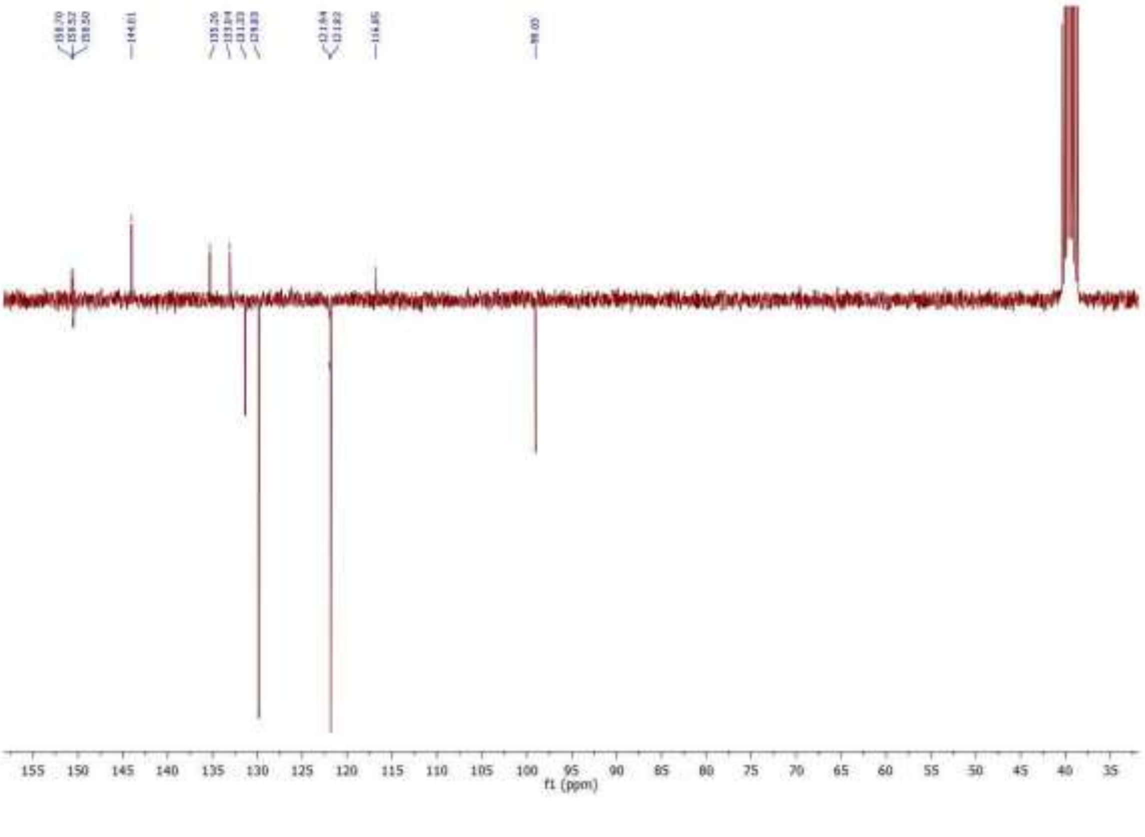

Fig. S17 a) ^{1}H NMR and b) ^{13}C NMR of compd. **8c**

a)

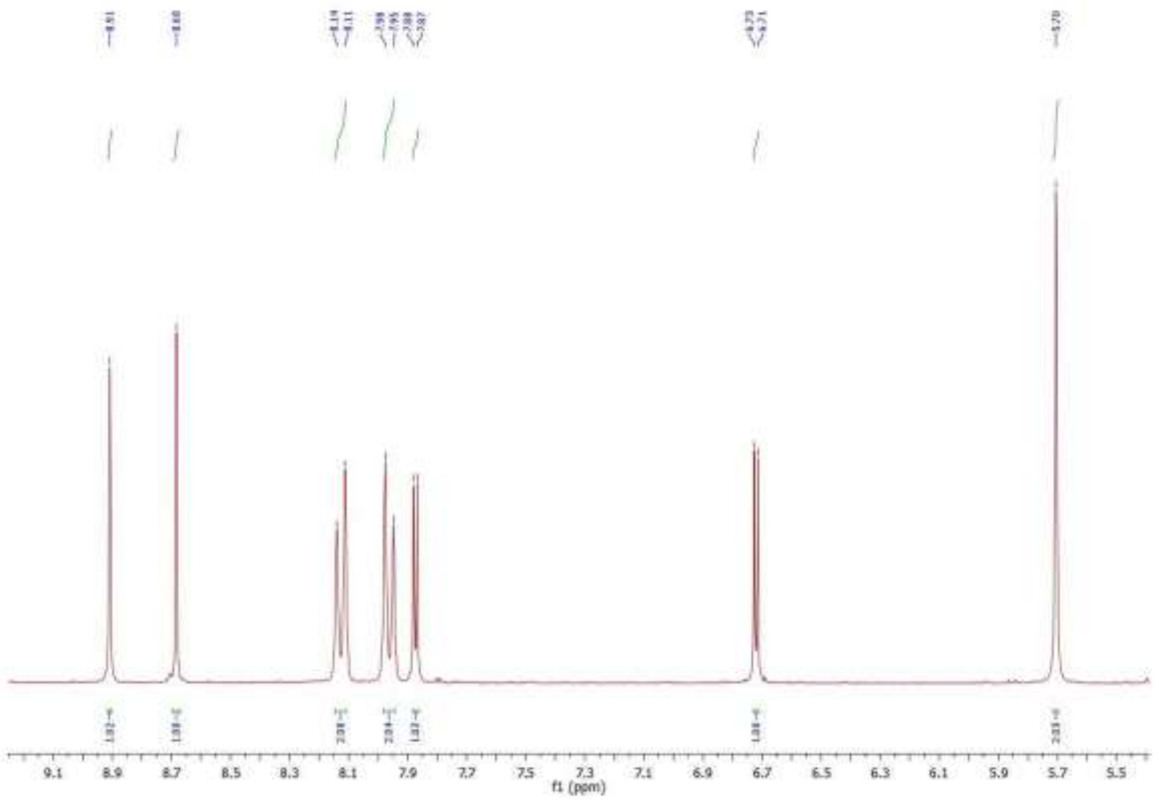

b)

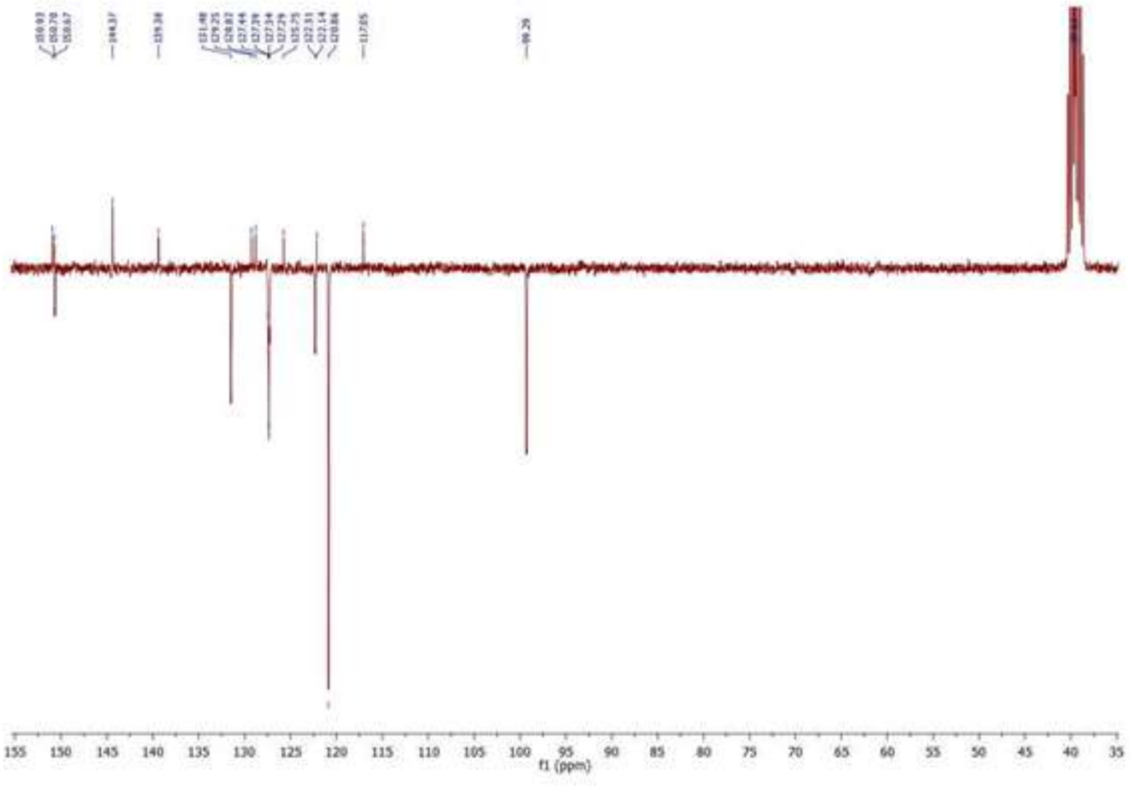

Fig. S18 a) ^{1}H NMR and b) ^{13}C NMR of compd. **8d**

a)

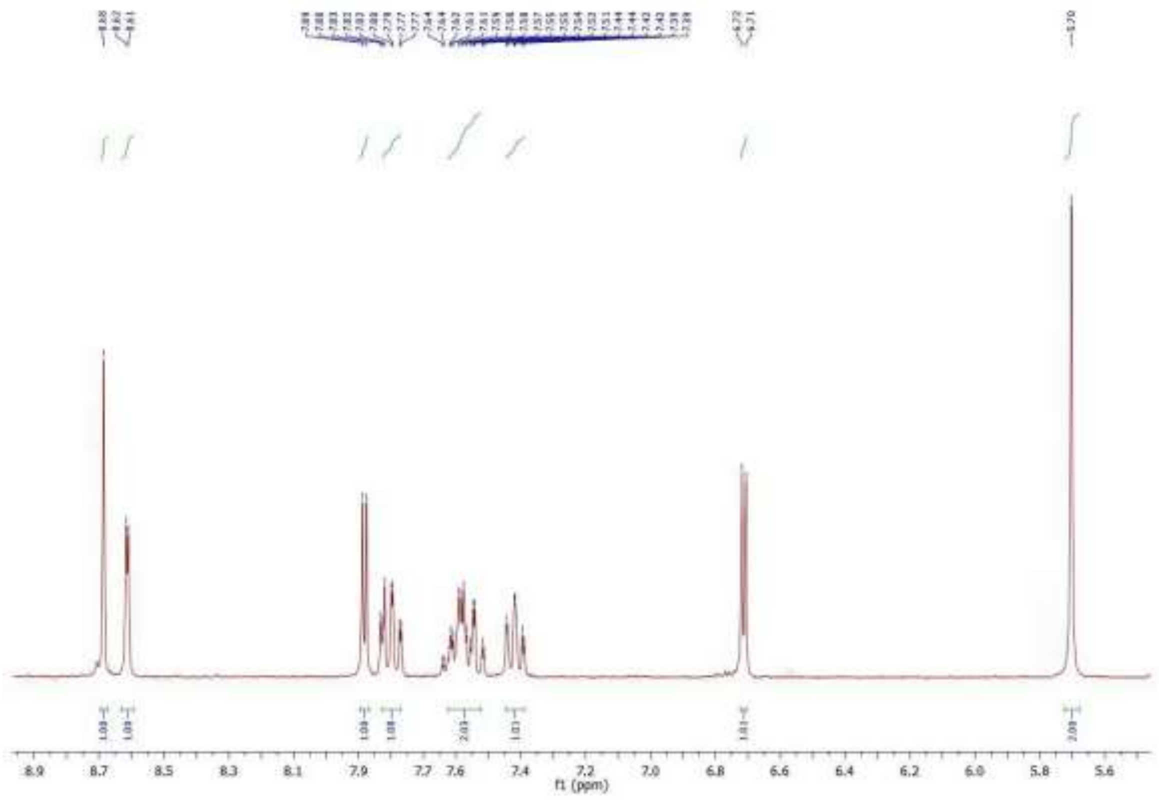

b)

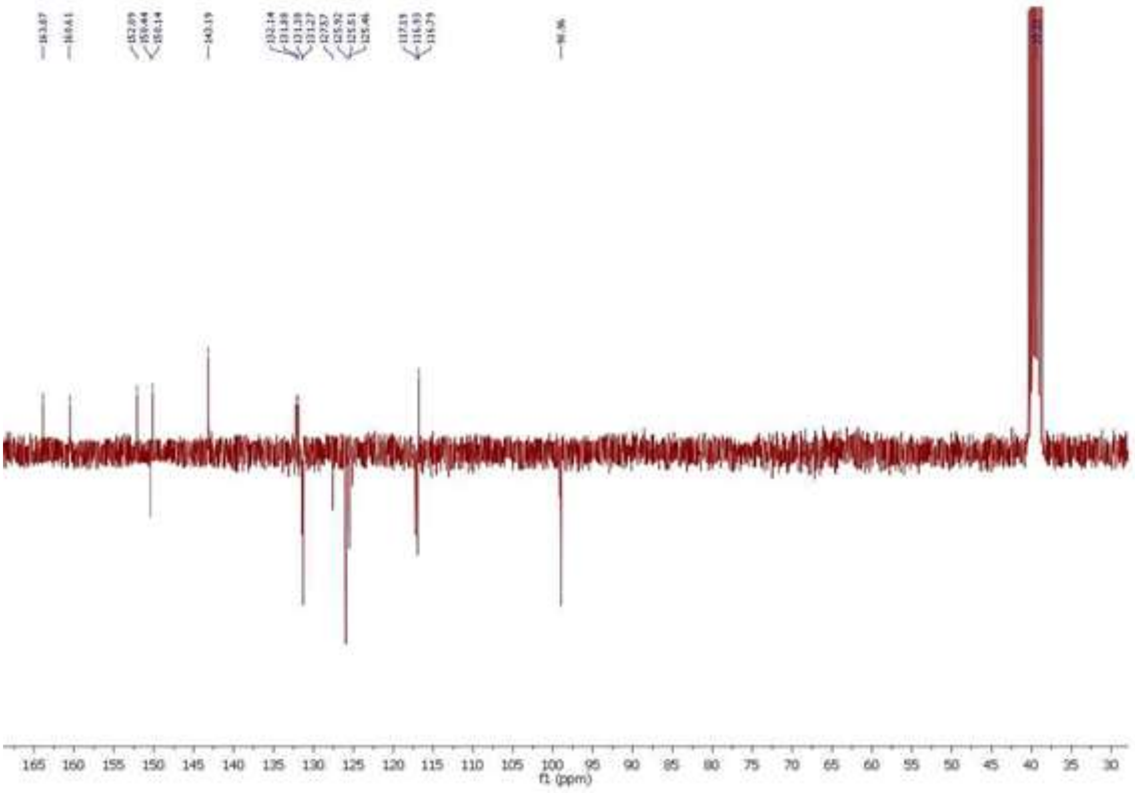

Fig. S19 a) ^{1}H NMR and b) ^{13}C NMR of compd. **8e**

a)

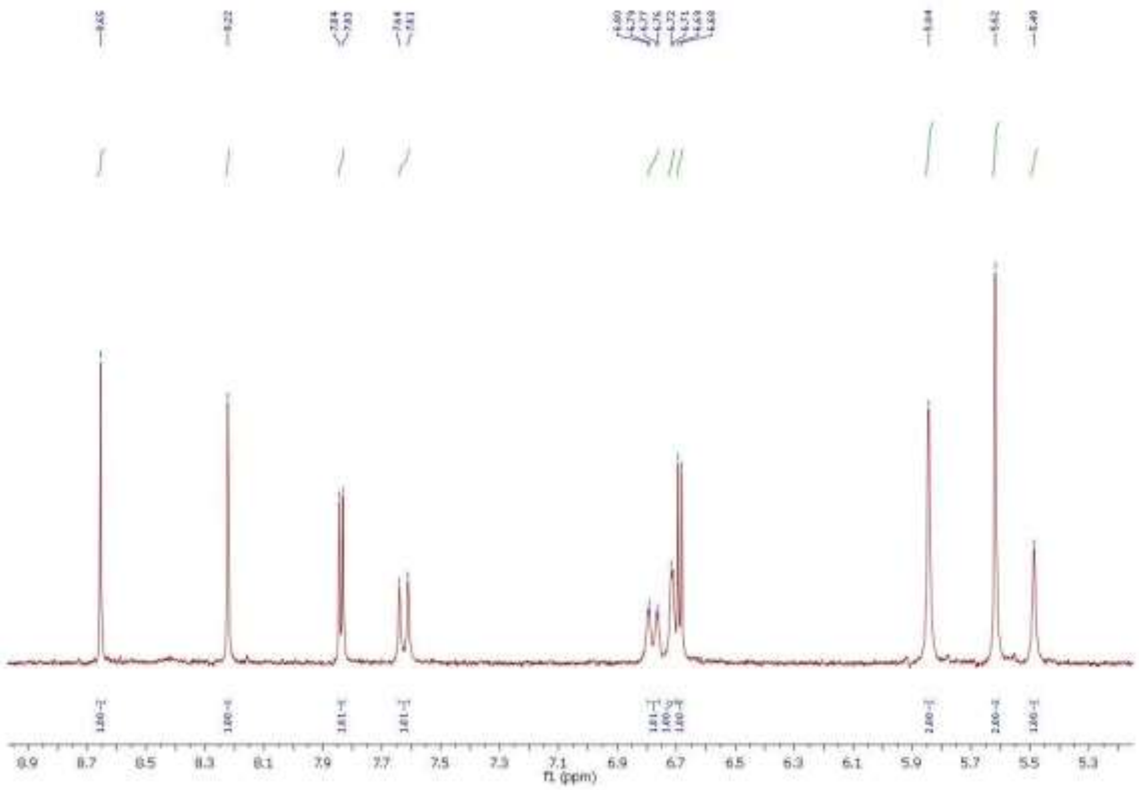

b)

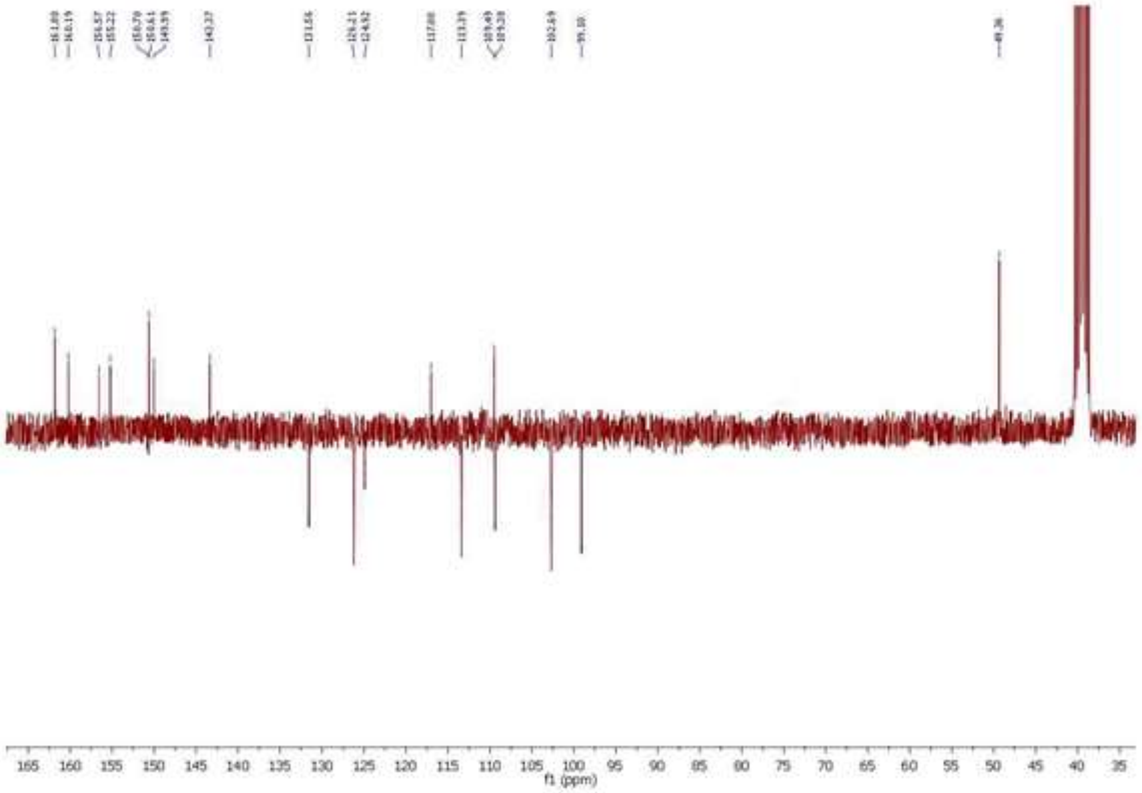

Fig. S20 a) ^{1}H NMR and b) ^{13}C NMR of compd. **9c**

a)

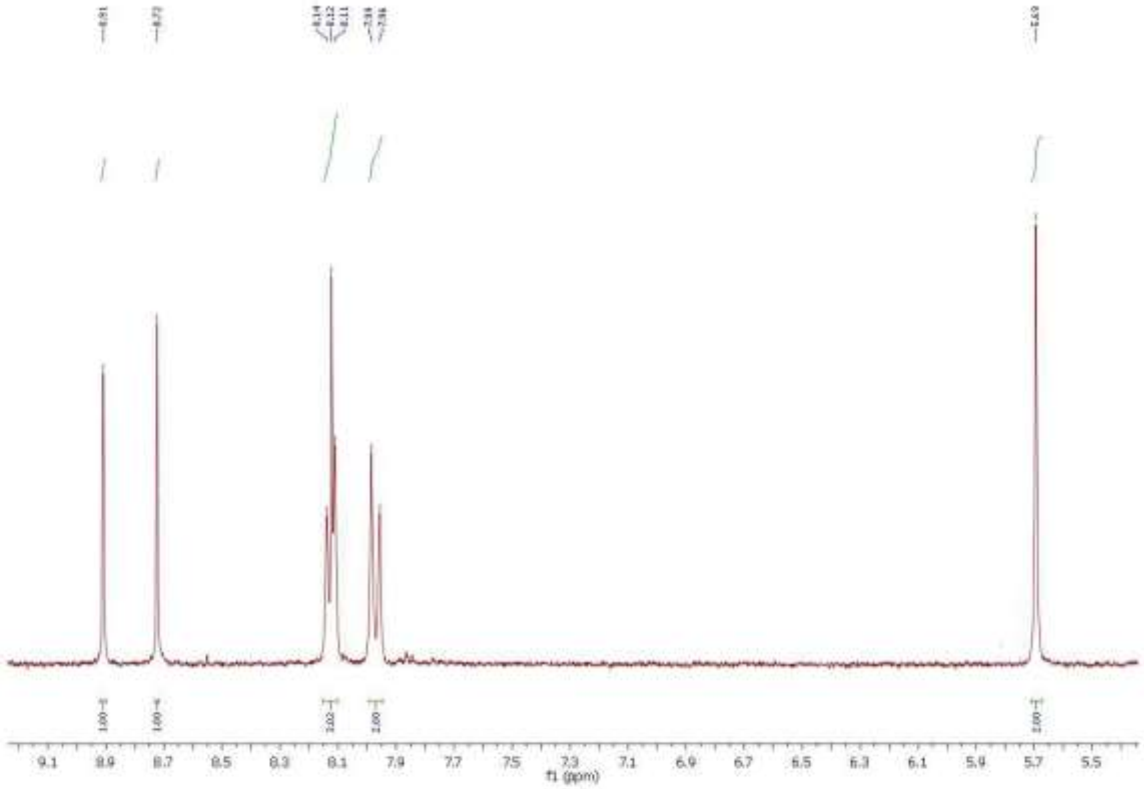

b)

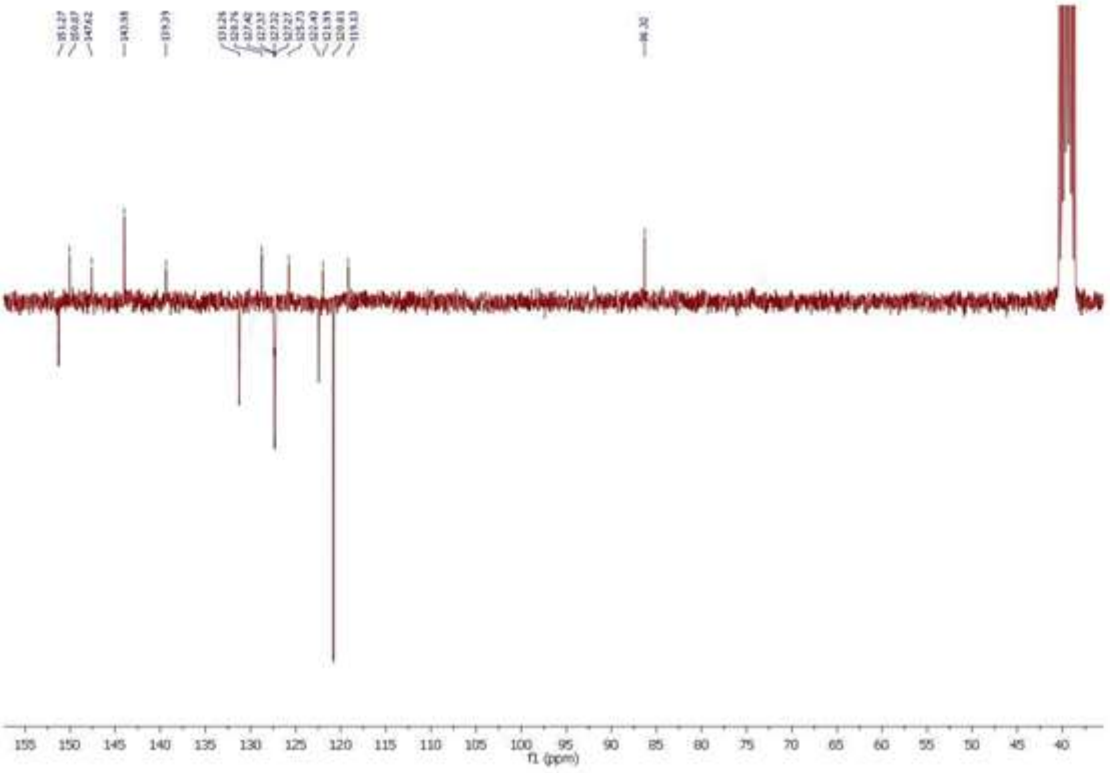

Fig. S21 a) ^{1}H NMR and b) ^{13}C NMR of compd. **9d**

a)

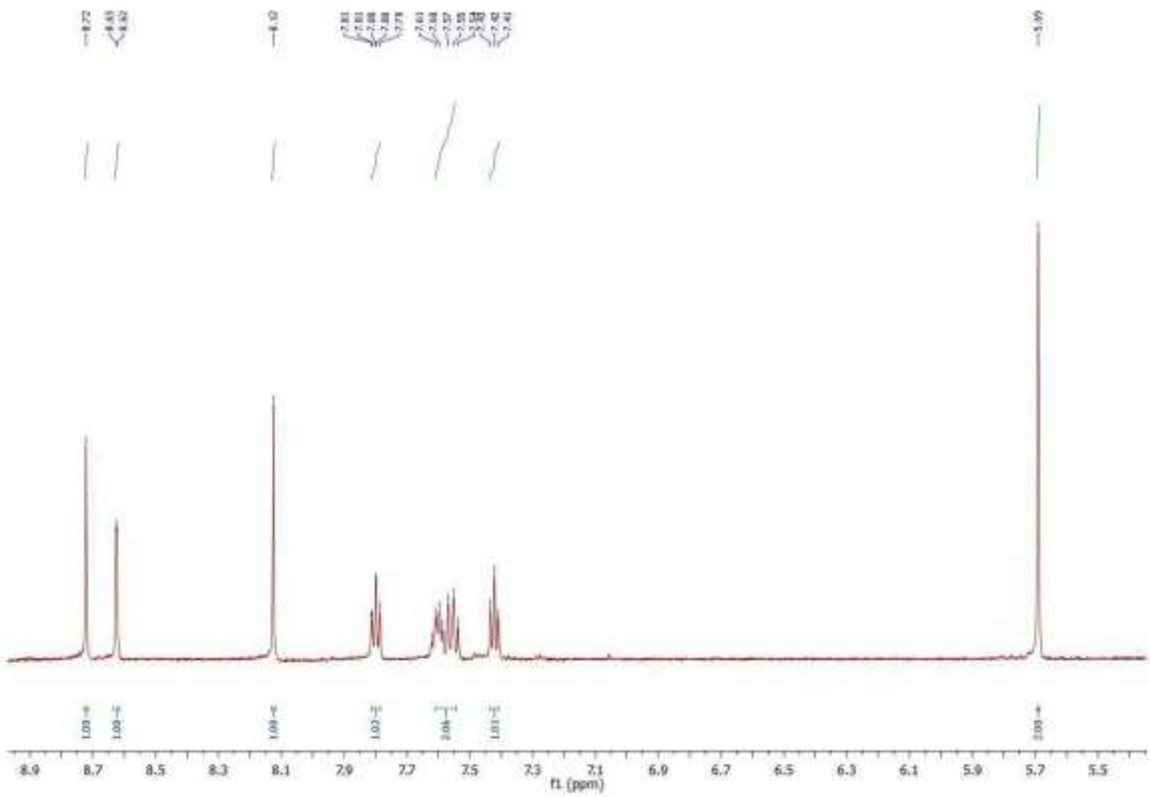

b)

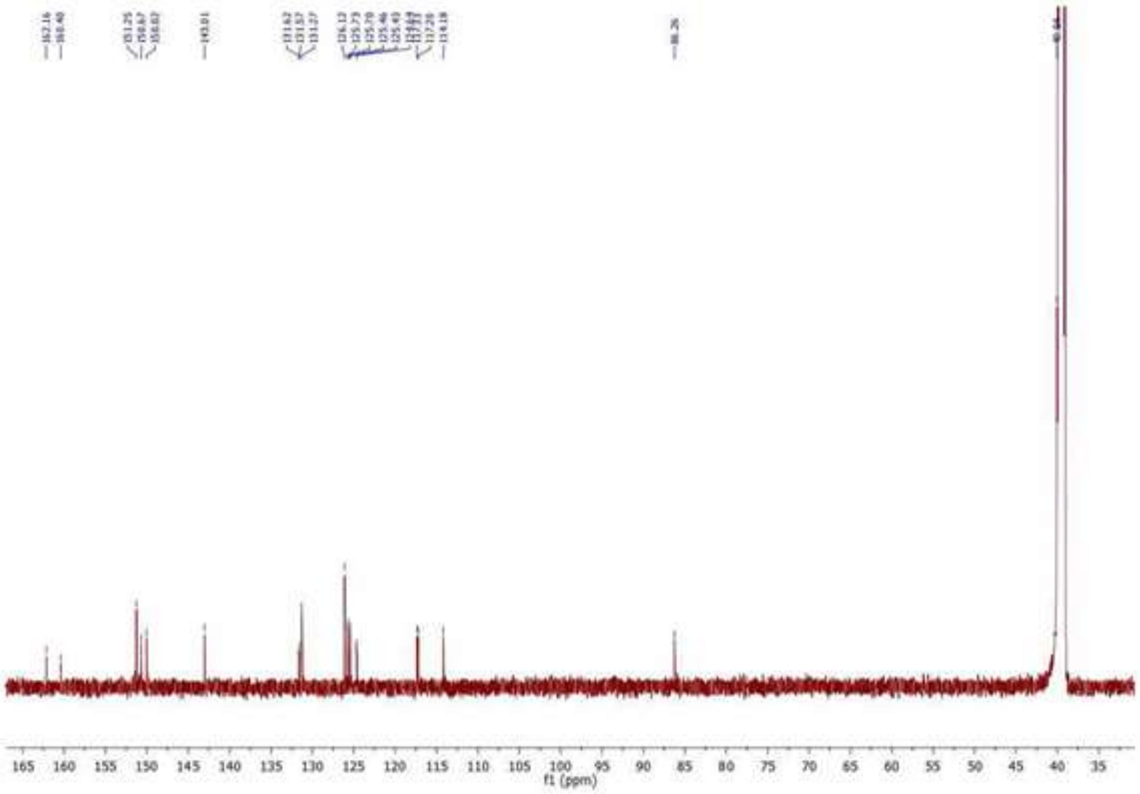

Fig. S22 a) ^{1}H NMR and b) ^{13}C NMR of compd. **9e**

a)

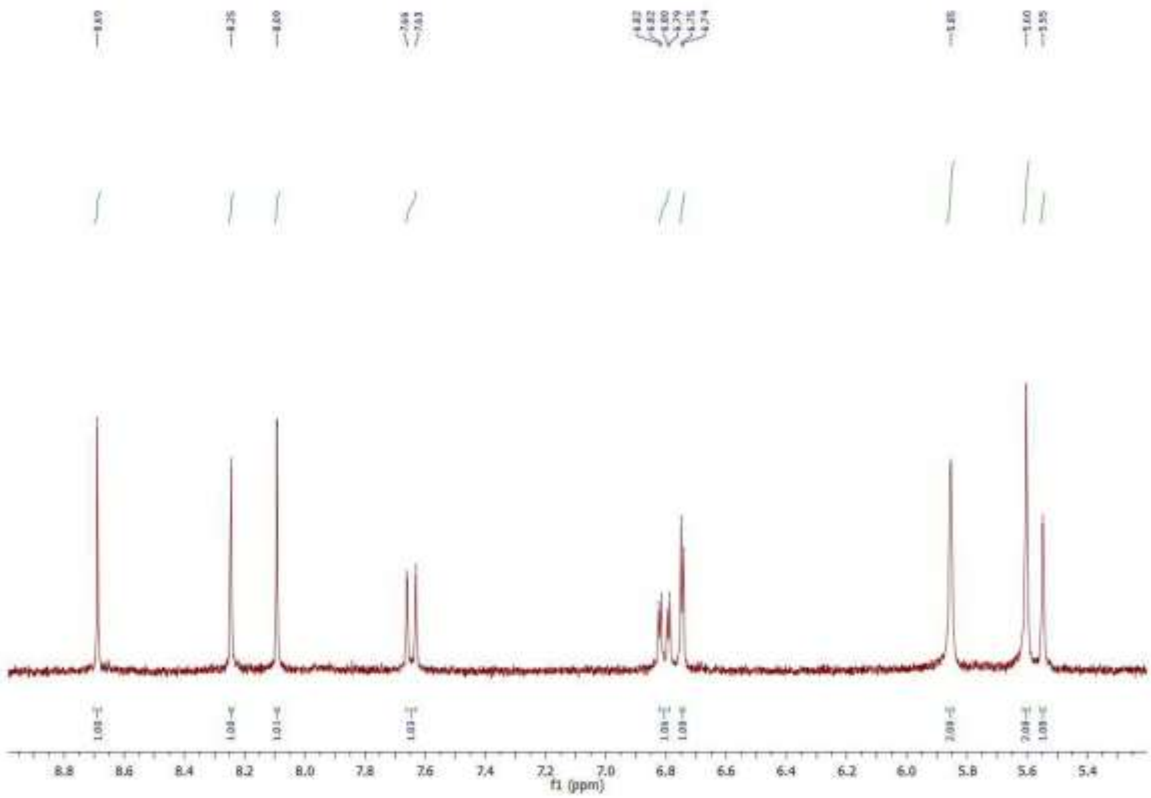

b)

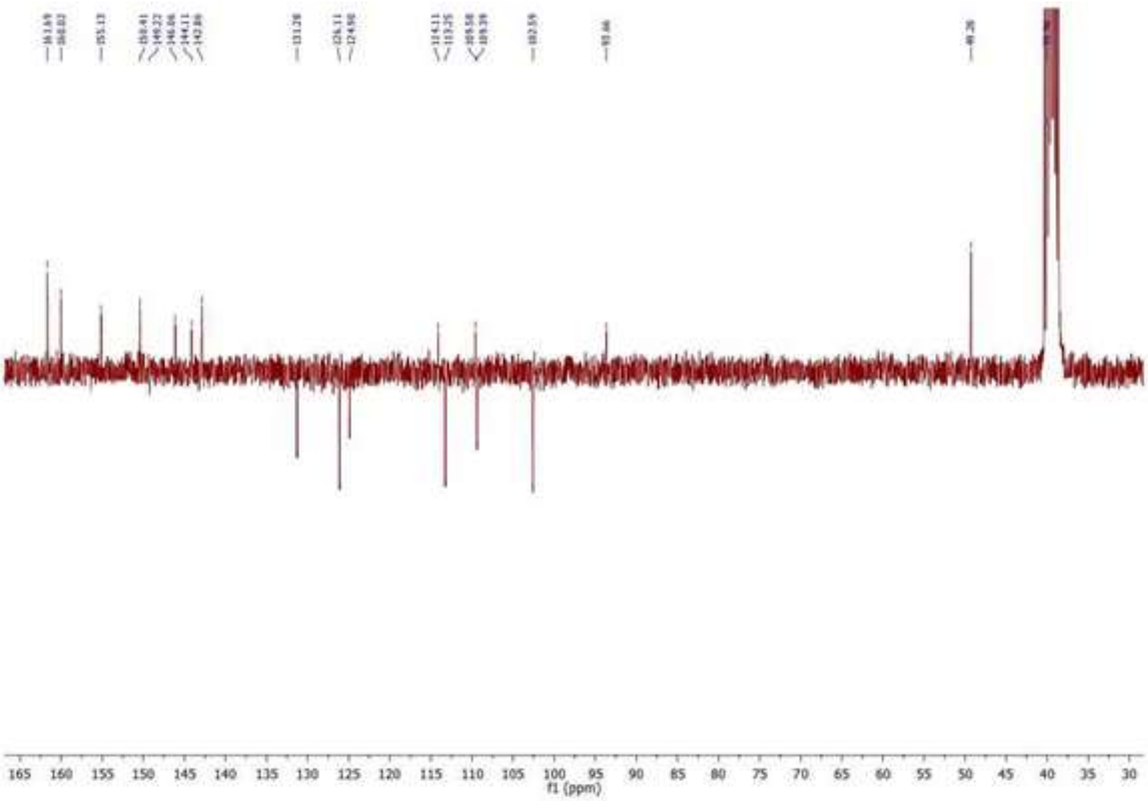

Fig. S23 a) ^{1}H NMR and b) ^{13}C NMR of compd. **10a**

a)

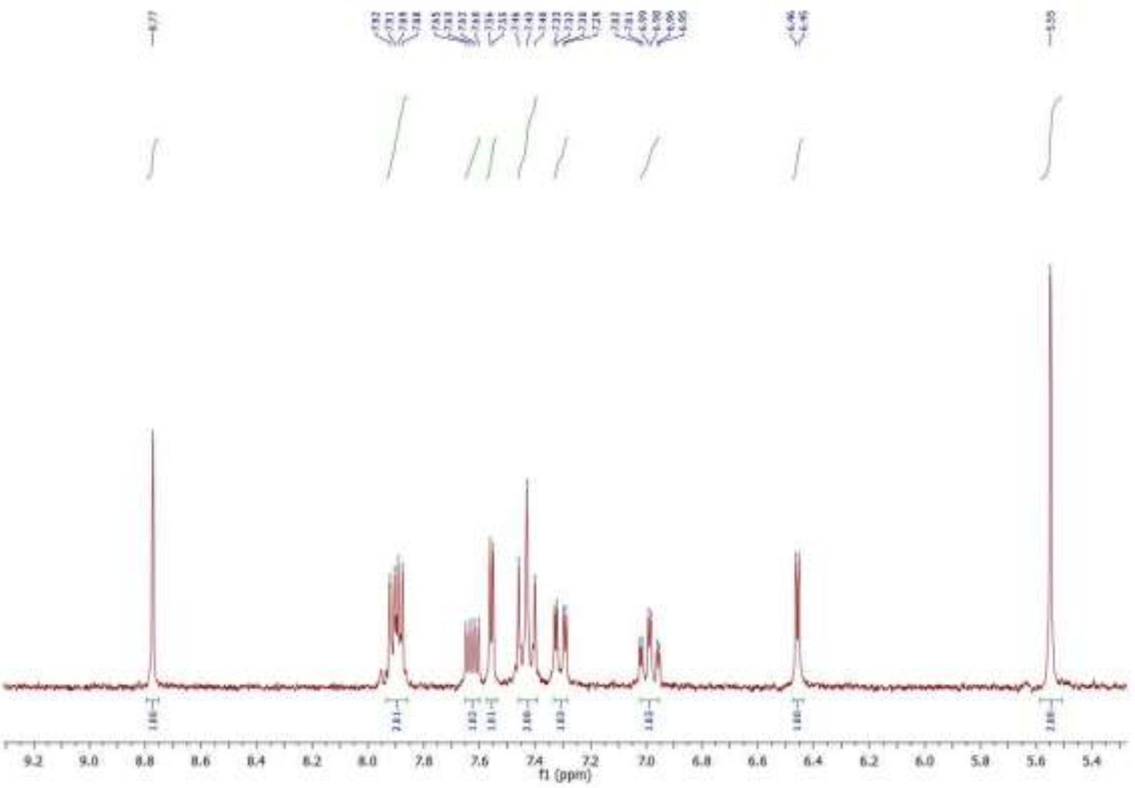

b)

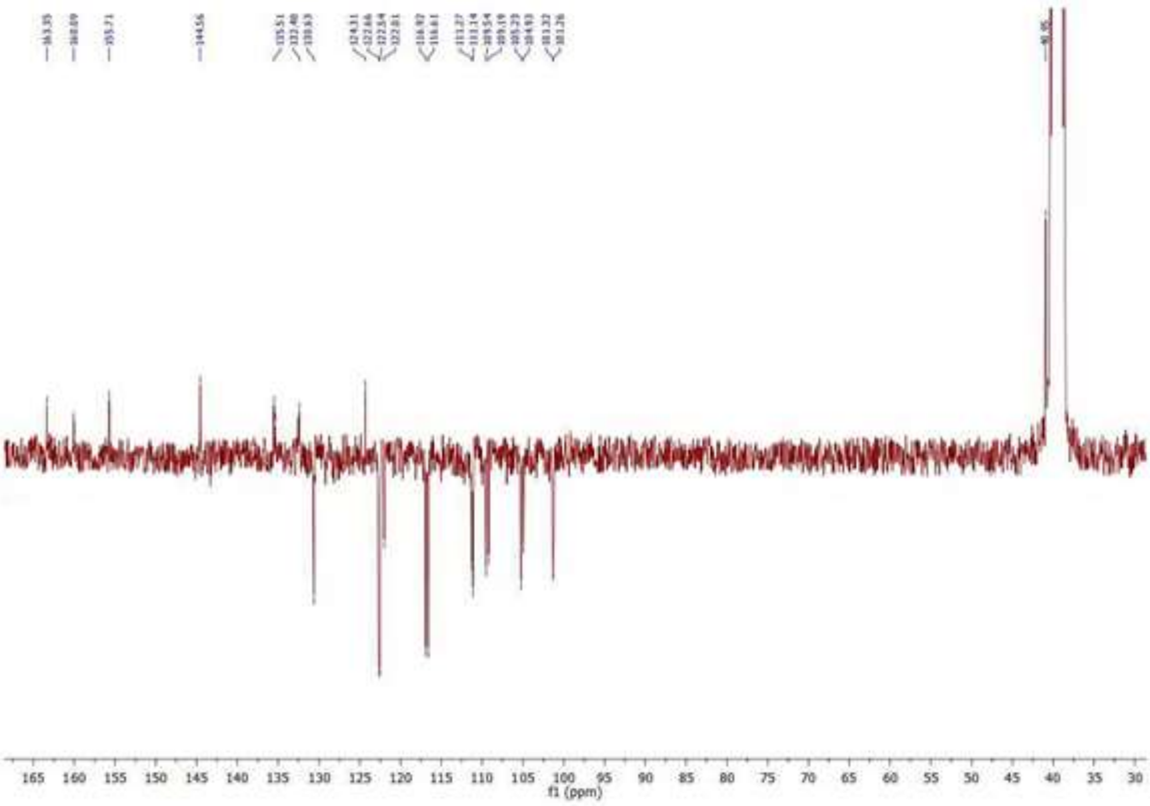

Fig. S24 a) ^{1}H NMR and b) ^{13}C NMR of compd. **10b**

a)

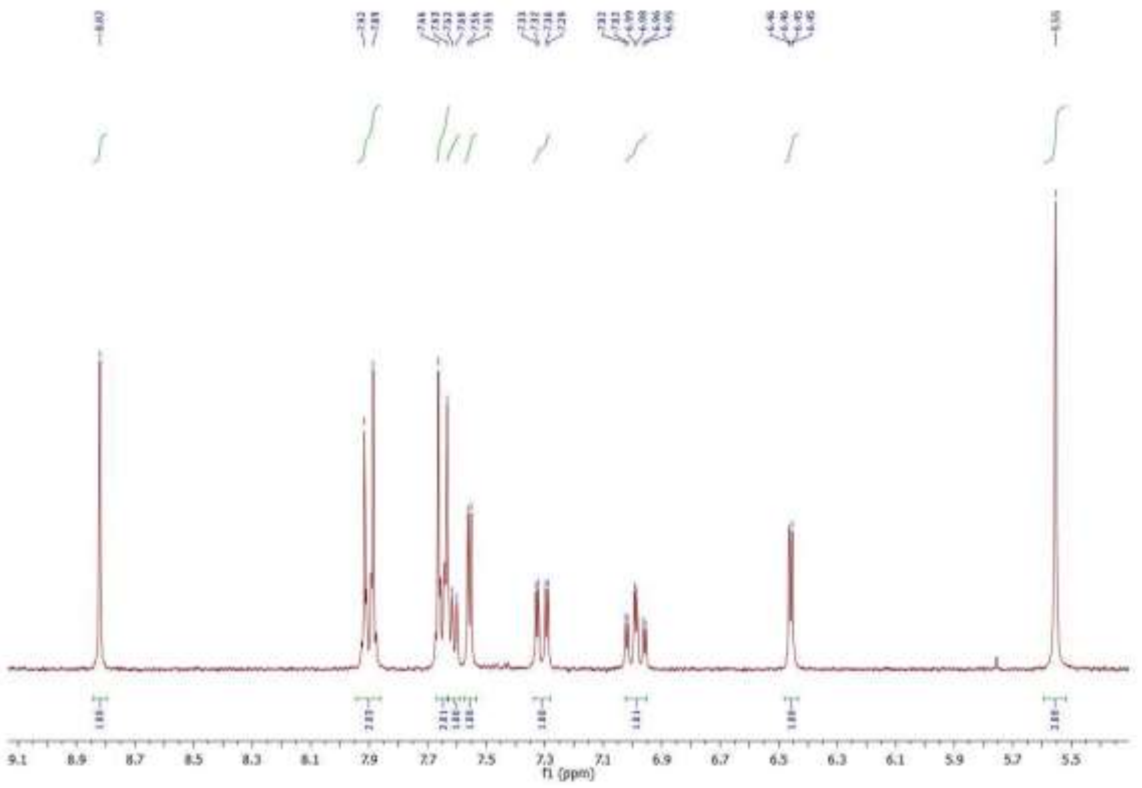

b)

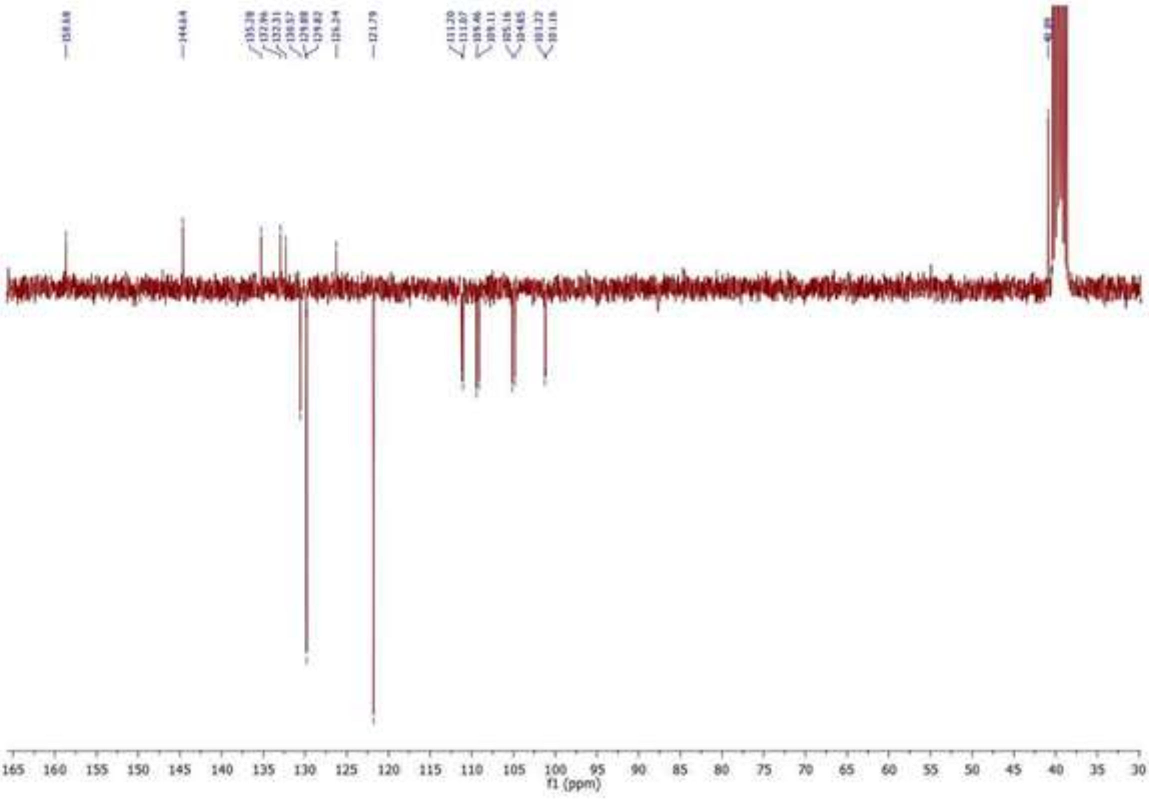

Fig. S25 a) ^{1}H NMR and b) ^{13}C NMR of compd. **10c**

a)

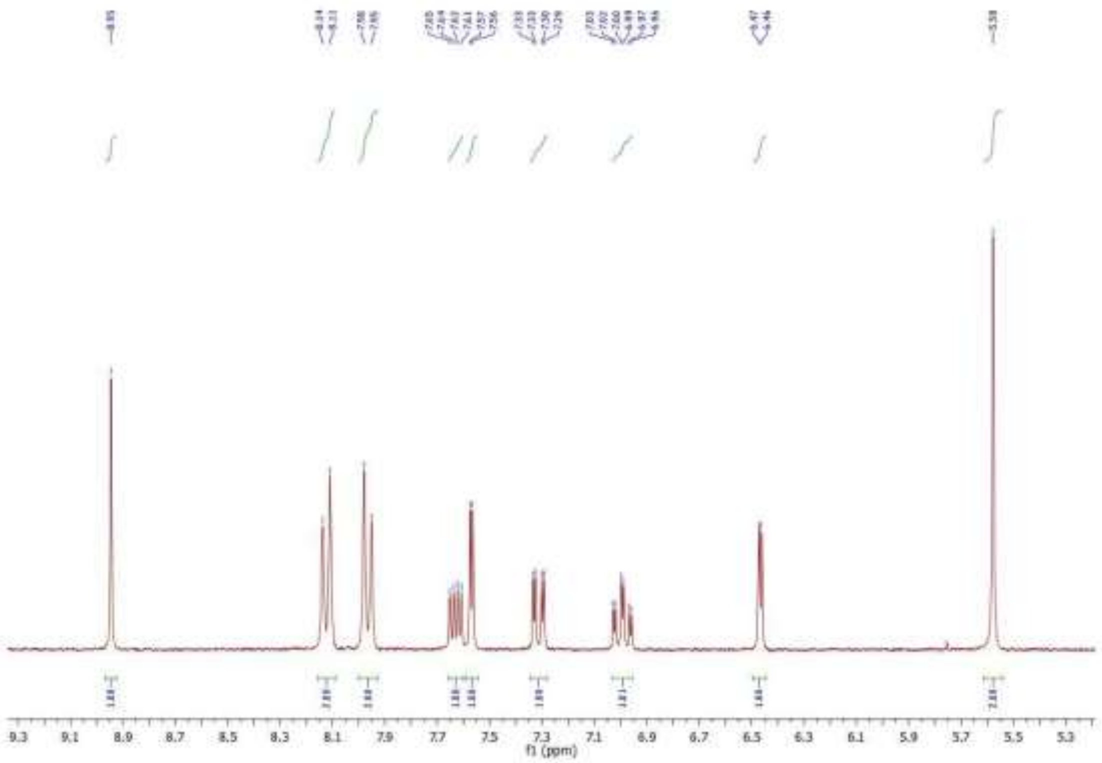

b)

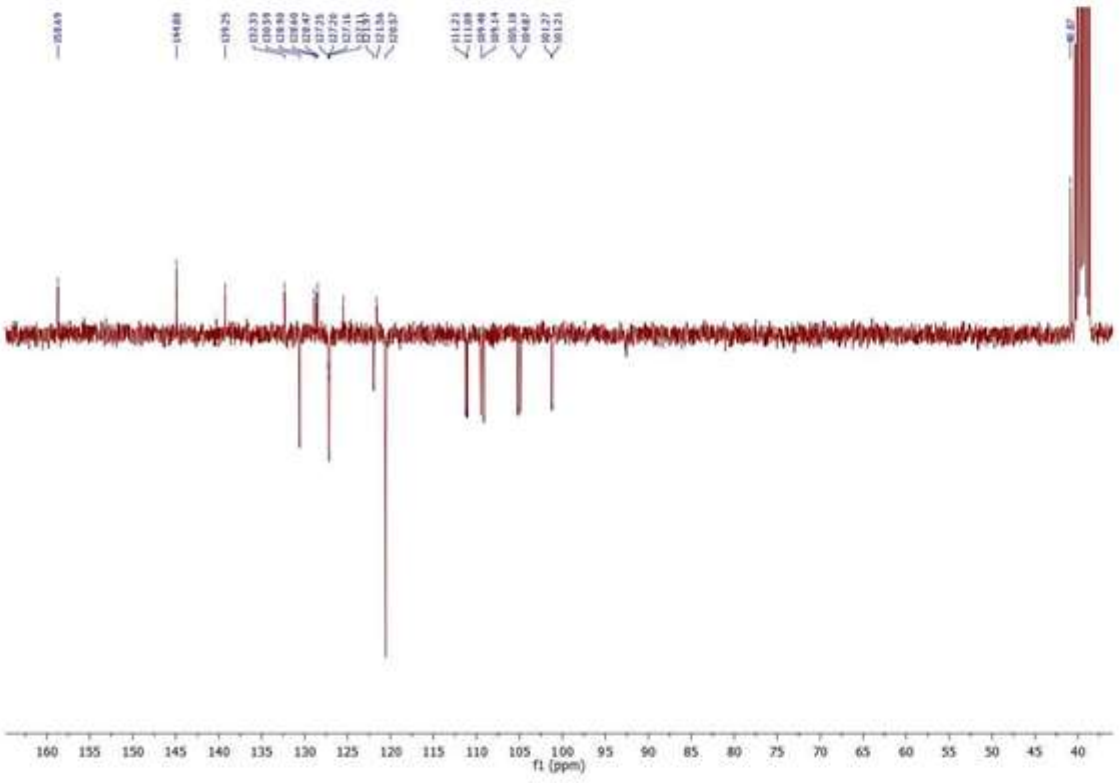

Fig. S26 a) ^{1}H NMR and b) ^{13}C NMR of compd. **10d**

a)

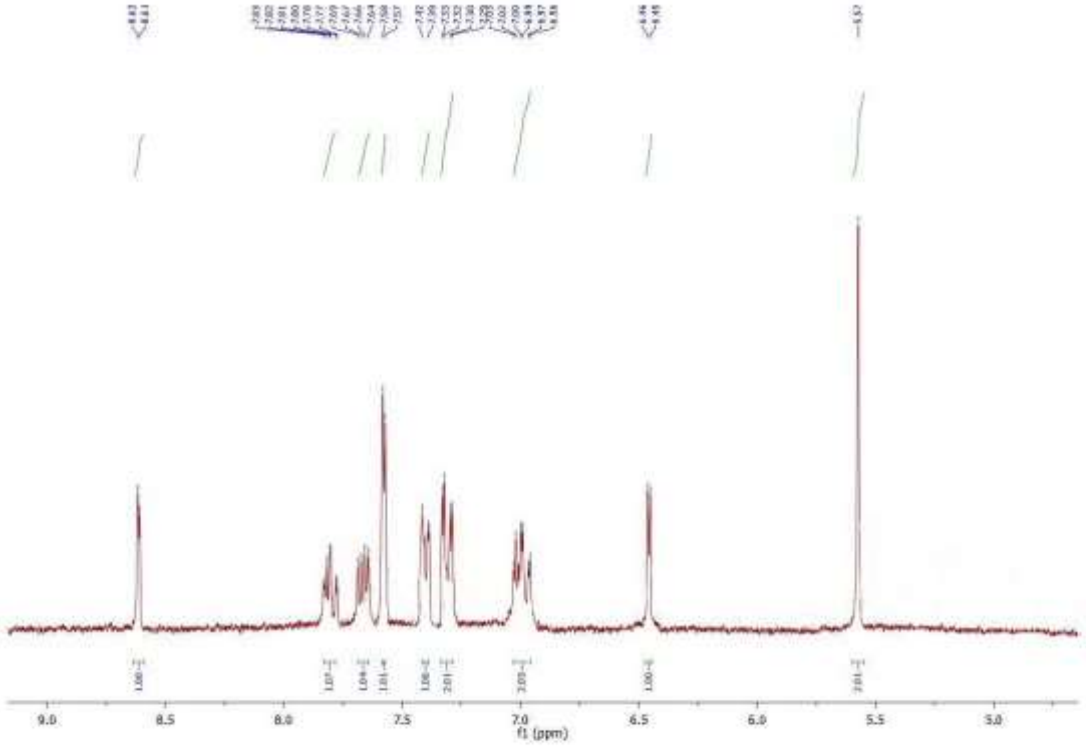

b)

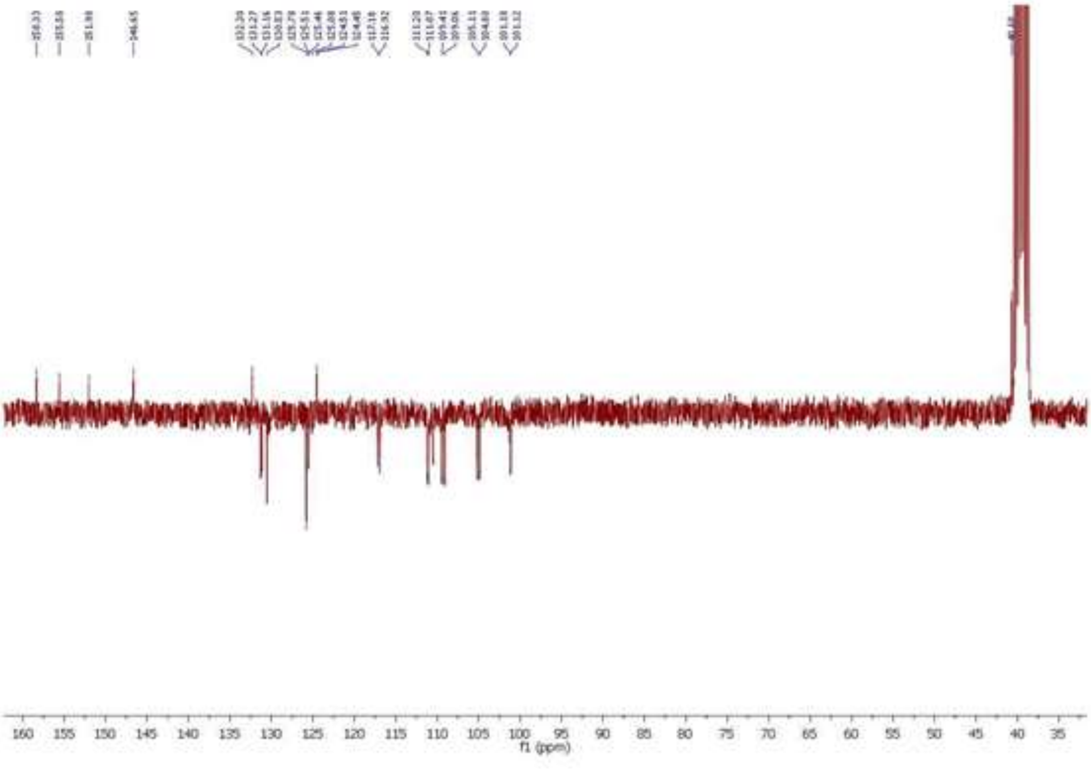

Fig. S27 a) ^{1}H NMR and b) ^{13}C NMR of compd. **10e**

a)

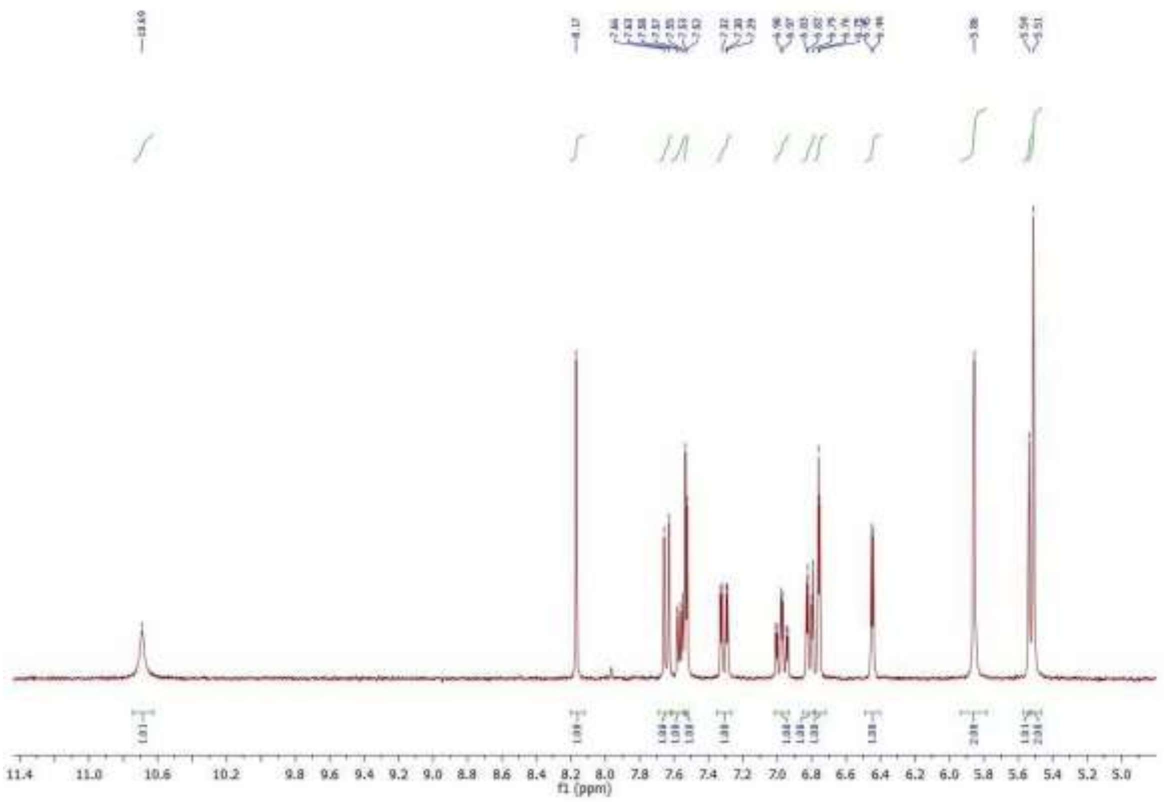

b)

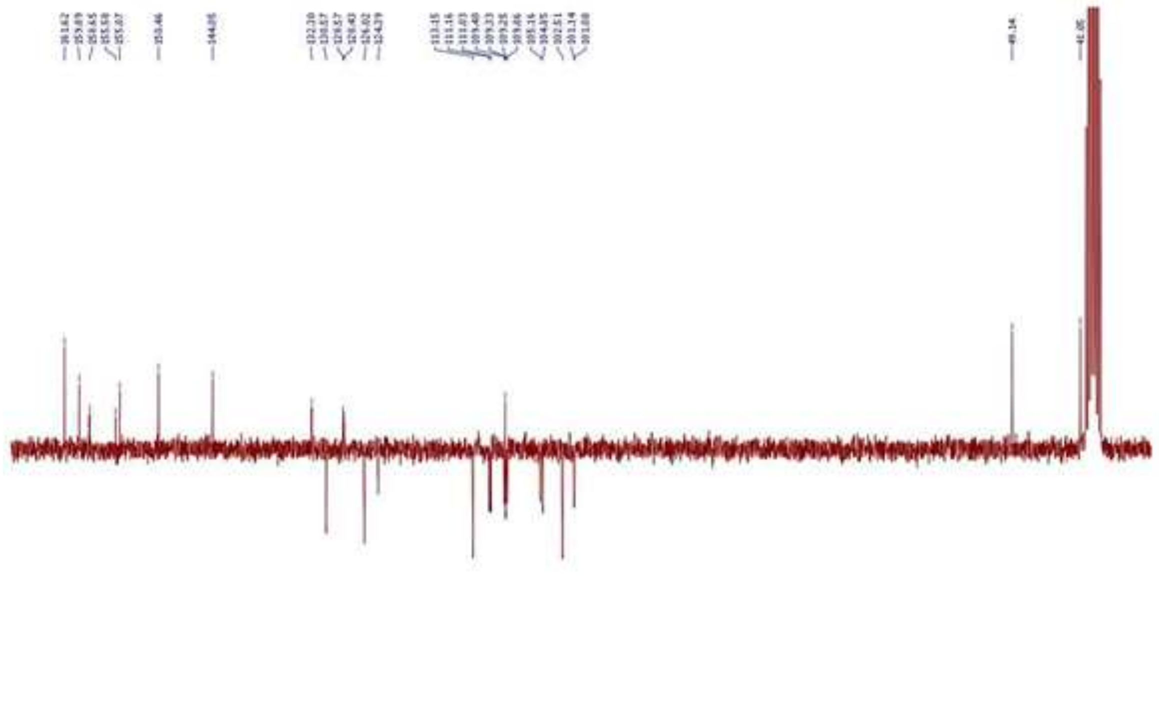

Fig. S28 a) ^{1}H NMR and b) ^{13}C NMR of compd. **11a**

a)

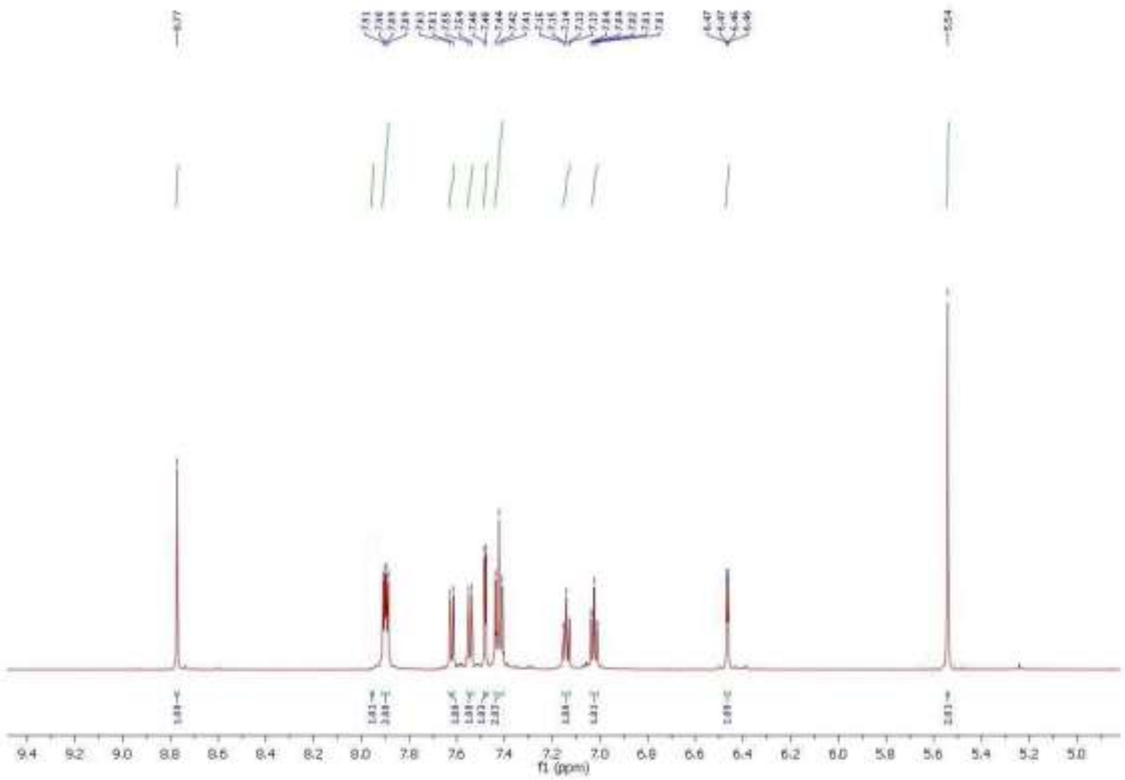

b)

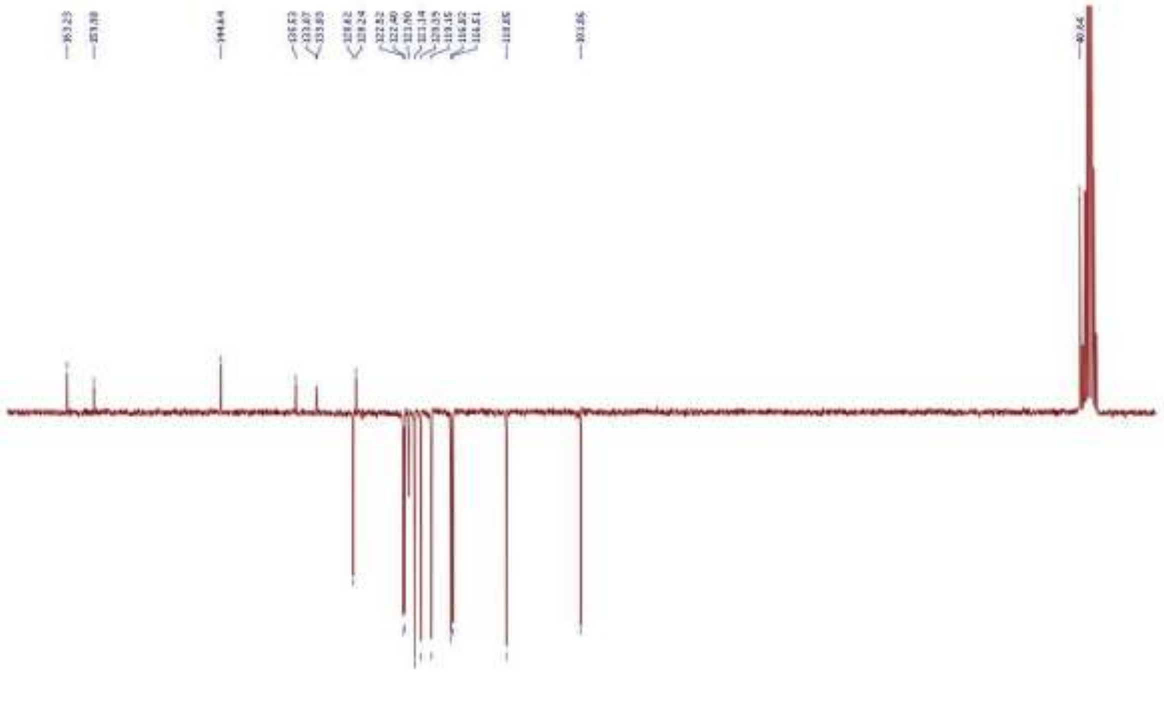

Fig. S29 a) ^{1}H NMR and b) ^{13}C NMR of compd. **11b**

a)

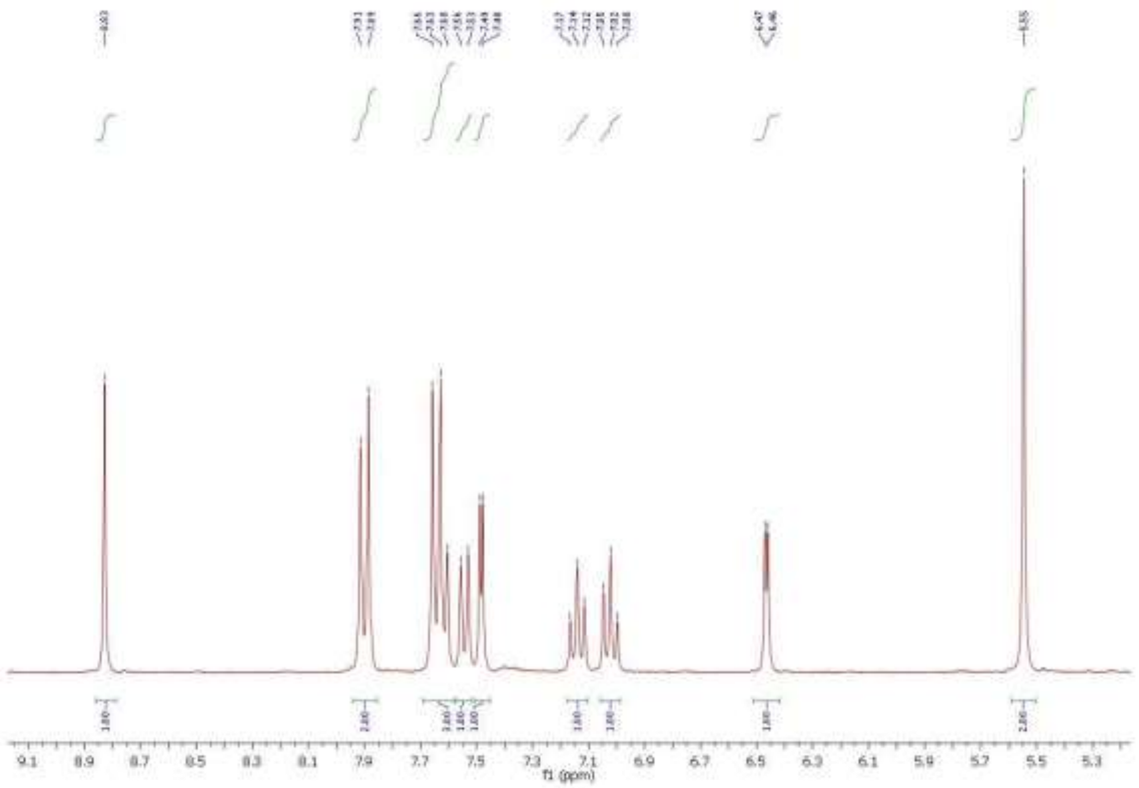

b)

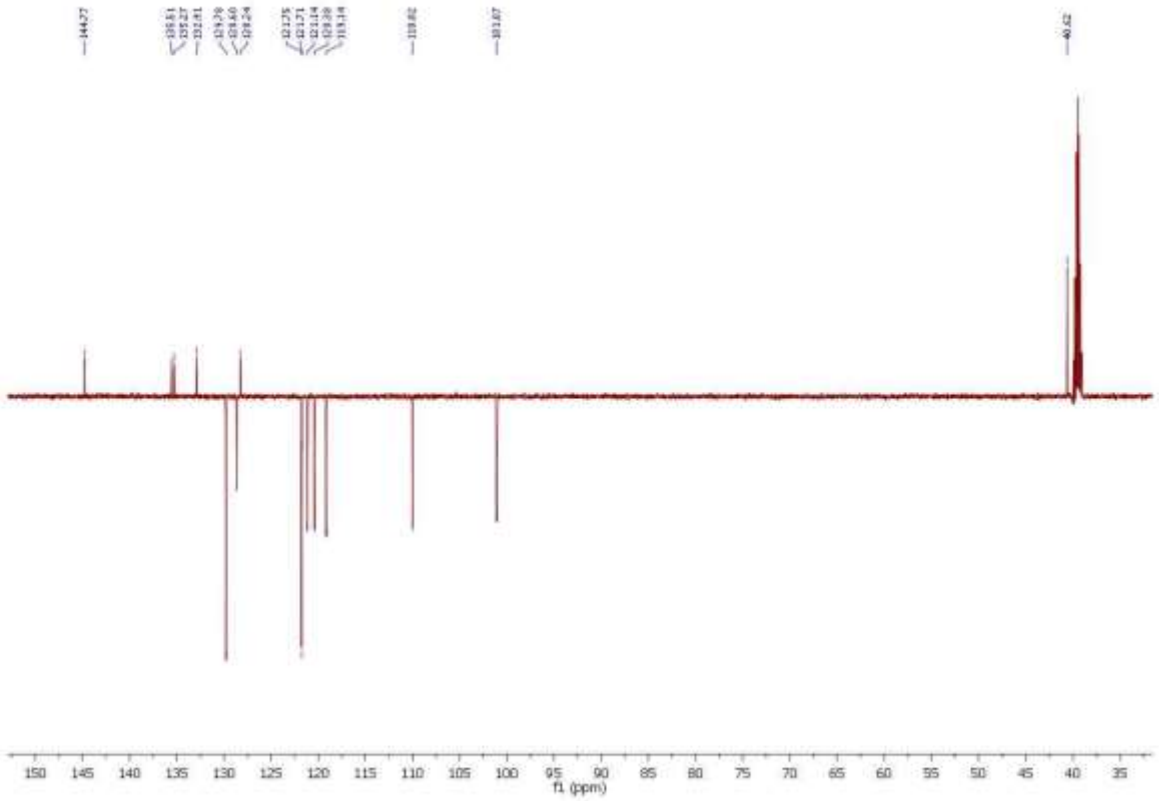

Fig. S30 a) ^{1}H NMR and b) ^{13}C NMR of compd. **11c**

a)

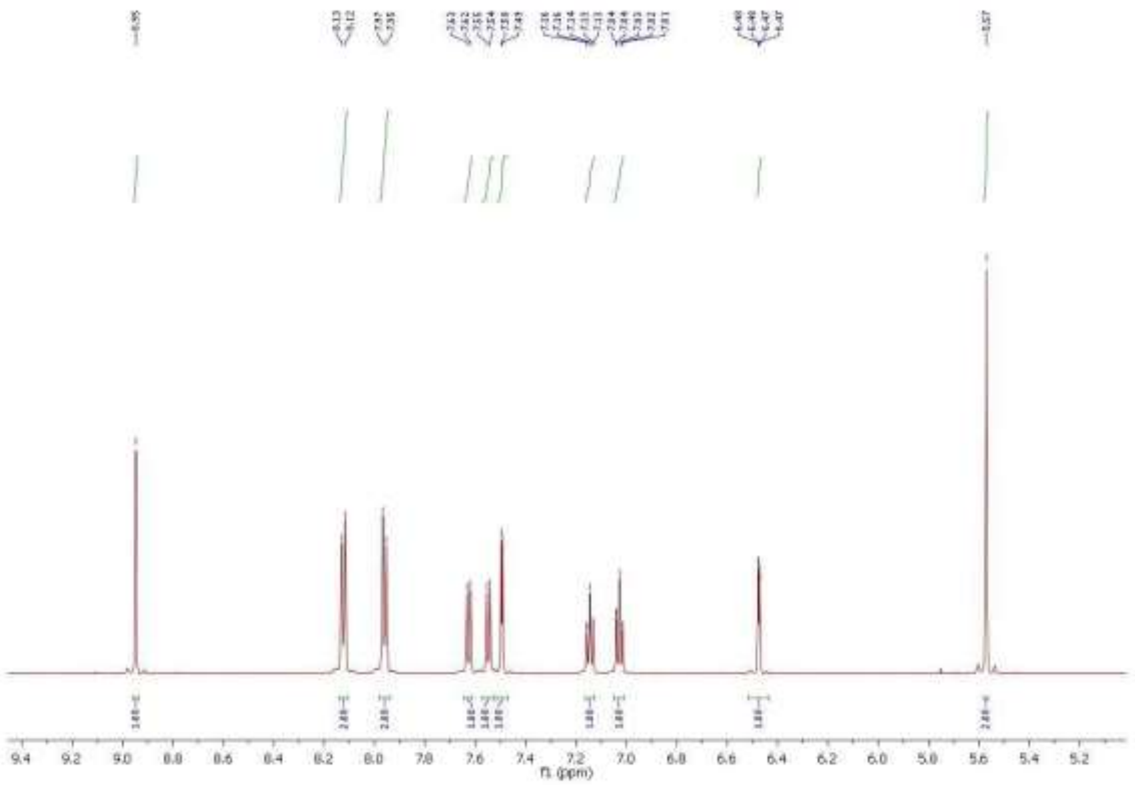

b)

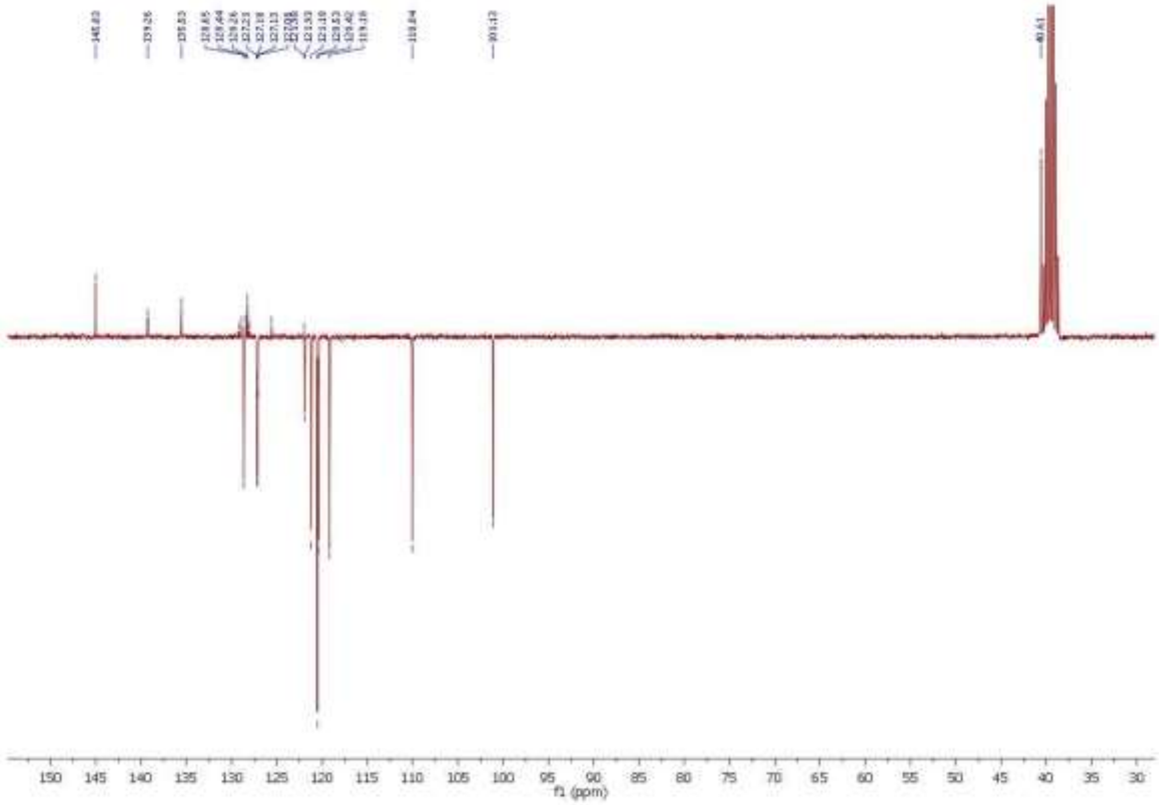

Fig. S31 a) ^{1}H NMR and b) ^{13}C NMR of compd. **11d**

a)

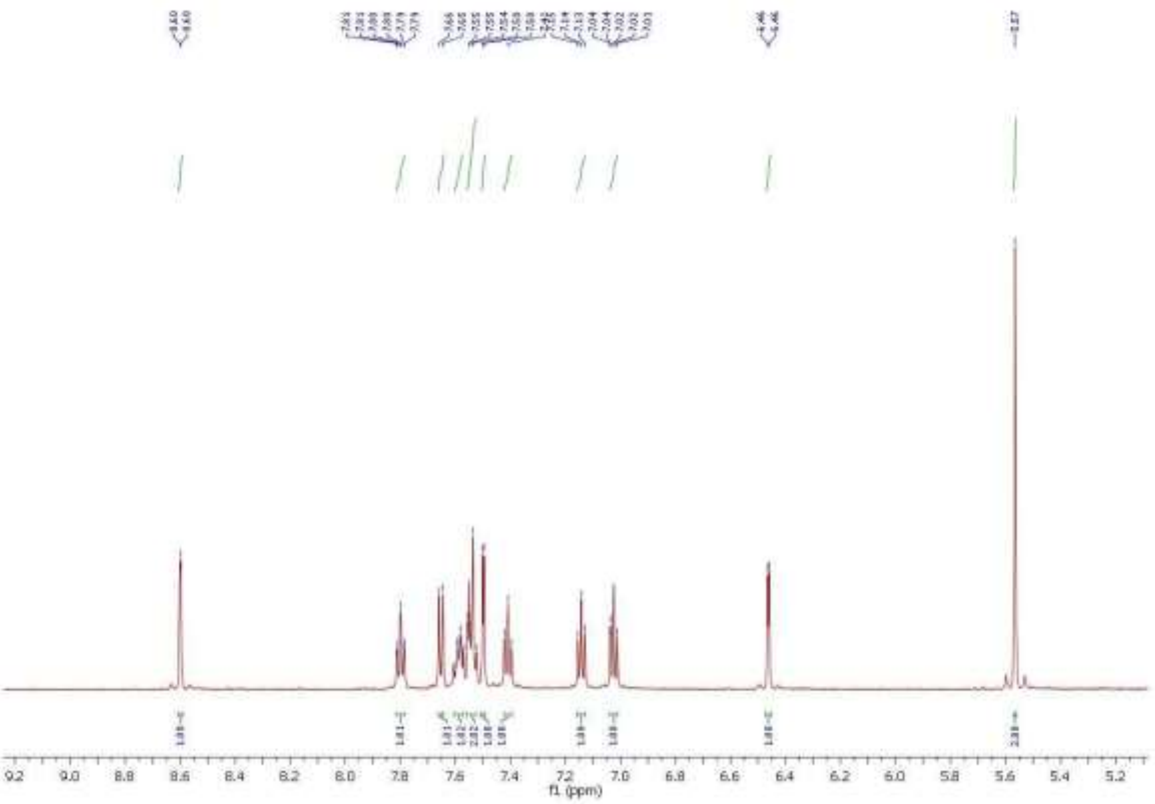

b)

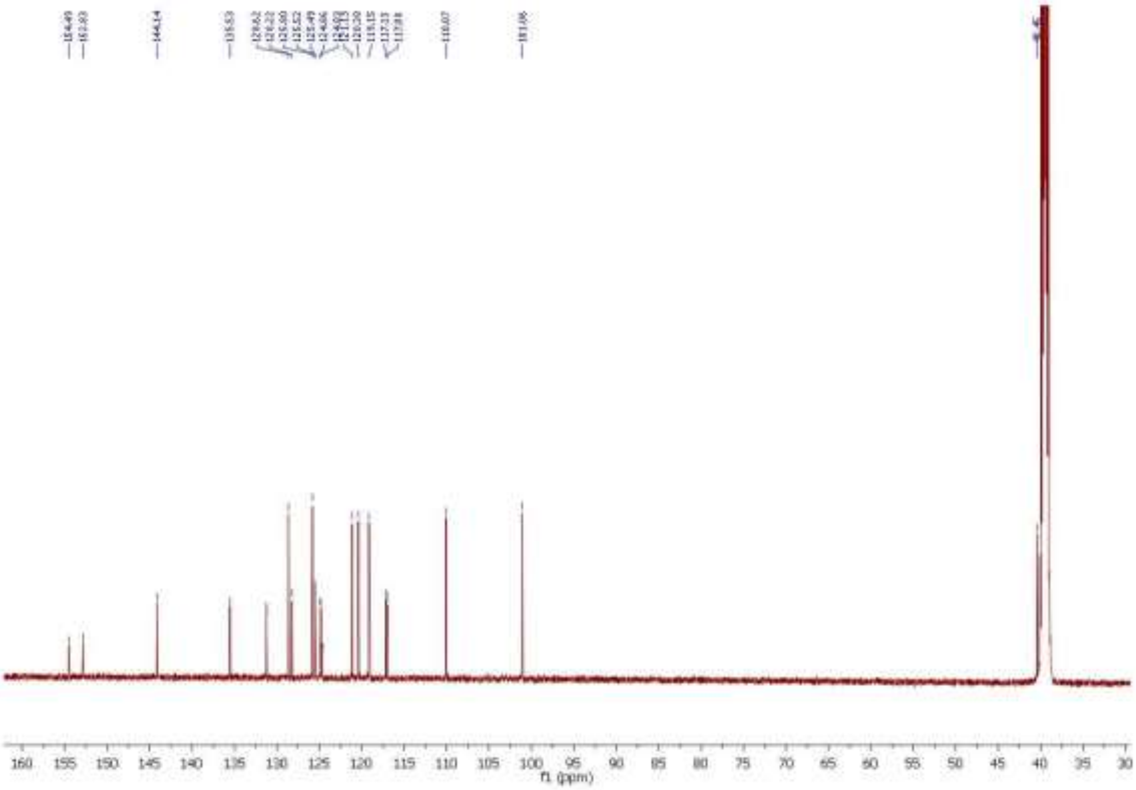

Fig. S32 a) ^{1}H NMR and b) ^{13}C NMR of compd. **11e**

a)

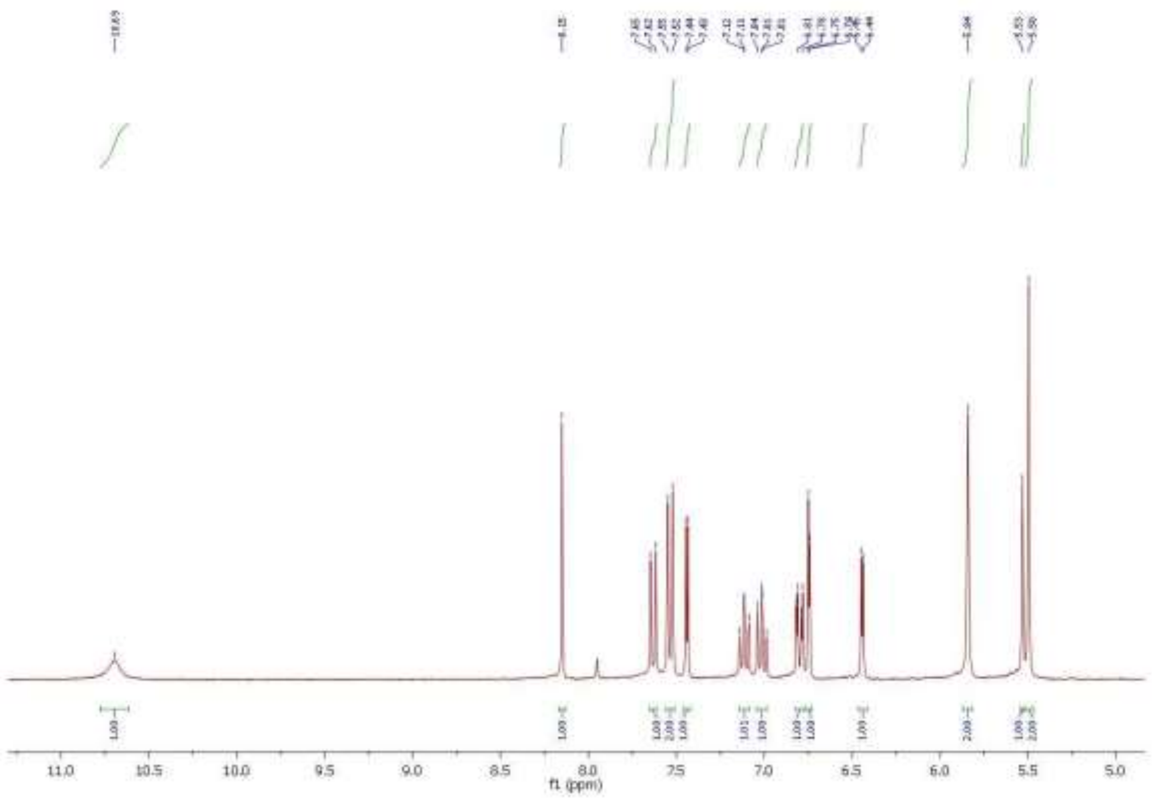

b)

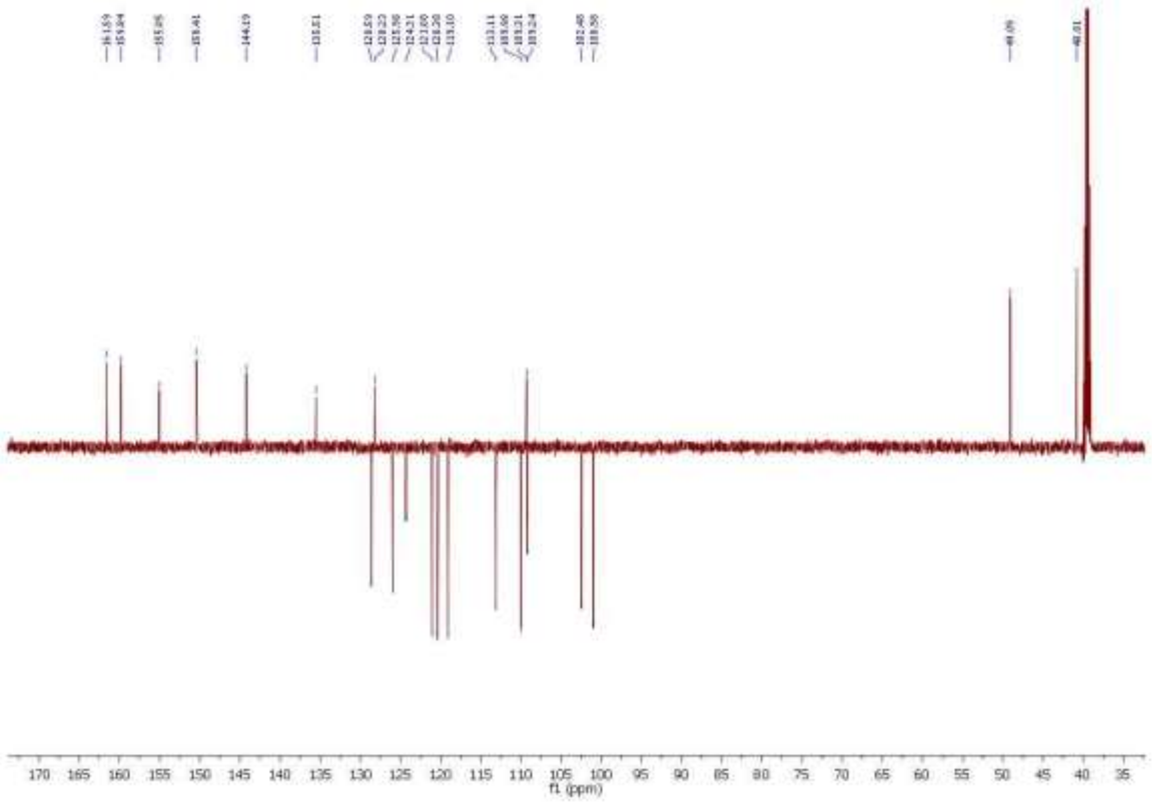

Supplement: IENZ_1414807_Supplementary_Material.pdf [file IENZ_A_1414807_SM2294.pdf]
